# In silico comparative analysis of GGDEF and EAL domain signaling proteins from the *Azospirillum* genomes

**DOI:** 10.1186/s12866-018-1157-0

**Published:** 2018-03-09

**Authors:** Alberto Ramírez Mata, César Millán Pacheco, José F. Cruz Pérez, Martha Minjárez Sáenz, Beatriz E. Baca

**Affiliations:** 1 Centro de Investigaciones en Ciencias Microbiológicas, Benemérita Universidad Autónoma de Puebla. Edif. IC11, Ciudad Universitaria, Col. San Manuel Puebla Pue, CP72570 Puebla, Mexico; 2 Facultad de Farmacia, Universidad Autónoma del Estado de Morelos, Av. Universidad #1001, Col. Chamilpa, C.P, 62209 Cuernavaca, Morelos Mexico

**Keywords:** *Azospirillum*, Diguanylate cyclases, Phosphodiesterases, Second messenger cyclic-di-GMP, Biofilm

## Abstract

**Background:**

The cyclic-di-GMP (c-di-GMP) second messenger exemplifies a signaling system that regulates many bacterial behaviors of key importance; among them, c-di-GMP controls the transition between motile and sessile life-styles in bacteria. Cellular c-di-GMP levels in bacteria are regulated by the opposite enzymatic activities of diguanylate cyclases and phosphodiesterases, which are proteins that have GGDEF and EAL domains, respectively. *Azospirillum* is a genus of plant-growth-promoting bacteria, and members of this genus have beneficial effects in many agronomically and ecologically essential plants. These bacteria also inhabit aquatic ecosystems, and have been isolated from humus-reducing habitats. Bioinformatic and structural approaches were used to identify genes predicted to encode GG[D/E]EF, EAL and GG[D/E]EF-EAL domain proteins from nine genome sequences.

**Results:**

The analyzed sequences revealed that the genomes of *A. humicireducens* SgZ-5^T^, *A. lipoferum* 4B, *Azospirillum* sp*.* B510, *A. thiophilum* BV-S^T^, *A. halopraeferens* DSM3675, *A. oryzae* A2P, and *A. brasilense* Sp7, Sp245 and Az39 encode for 29 to 41 of these predicted proteins. Notably, only 15 proteins were conserved in all nine genomes: eight GGDEF, three EAL and four GGDEF-EAL hybrid domain proteins, all of which corresponded to core genes in the genomes. The predicted proteins exhibited variable lengths, architectures and sensor domains. In addition, the predicted cellular localizations showed that some of the proteins to contain transmembrane domains, suggesting that these proteins are anchored to the membrane. Therefore, as reported in other soil bacteria, the *Azospirillum* genomes encode a large number of proteins that are likely involved in c-di-GMP metabolism. In addition, the data obtained here strongly suggest host specificity and environment specific adaptation.

**Conclusions:**

Bacteria of the *Azospirillum* genus cope with diverse environmental conditions to survive in soil and aquatic habitats and, in certain cases, to colonize and benefit their host plant. Gaining information on the structures of proteins involved in c-di-GMP metabolism in *Azospirillum* appears to be an important step in determining the c-di-GMP signaling pathways, involved in the transition of a motile cell towards a biofilm life-style, as an example of microbial genome plasticity under diverse in situ environments.

**Electronic supplementary material:**

The online version of this article (10.1186/s12866-018-1157-0) contains supplementary material, which is available to authorized users.

## Background

The *Azospirillum* genus, a member of the Alphaproteobacteria, is composed of nitrogen-fixing species that colonize the rhizosphere of plants; these species have been extensively studied due to their plant growth-promoting properties (PGPB) [1, 2], and recently *Azospirillum* strains were isolated from aquatic and humus-reducing ecosystems [3, 4]. To date, approximately 19 different species, isolated from wide range of geographical regions and from a large variety of soils, especially soils of tropical, subtropical and temperate regions, have been described [1, 2]. The best studied species, *Azospirillum lipoferum* and *Azospirillum brasilense*, were initially isolated from tropical forage grass in Brazil [5]. Nucleotide sequencing of the genomes of a number of *Azospirillum s*trains has been performed, enabling researchers to conduct in silico analyses of proteins that are potentially important in the interactions of these bacteria with the host plant and in their adaptability to either terrestrial or aquatic environments [6–14].

It is now well established that bacteria in natural environments persist by forming biofilms [15]. Bacteria are able to sense and respond to ecologically distinct abiotic and biotic conditions [15]. These systems are necessary of these bacteria to adapt to changing environmental conditions and to enable survival in highly competitive habitats, such as the plant rhizosphere, soil or aquatic environments. In addition, usually, bacteria must efficiently colonize the root surface and other diverse surfaces, to exert their beneficial effect, which implies that understanding the bacterial traits required for biofilm formation is crucial to understanding the mechanisms involved in colonization. In particular, motility, which involves the flagellar apparatus and the chemotatic response to root exudates, appears to be an important colonization attribute, as do the formation of cell aggregates and the production of capsular polysaccharides. Indeed, several chemotaxis and aerotaxis operons have been identified in *A. brasilense* [6, 16, 17], and different mutant strains are defective in biofilm formation and root surface colonization [18, 19].

Similarly, some of the genetic determinants involved in cell aggregation and flocculation in *A. brasilense* and *A. lipoferum* lead to the differentiation of cyst-like cells [20, 21] and appear to be important for root colonization [22, 23]. Indeed, cell morphology had an effect on the associated root colonization, and importantly, capsular polysaccharides production in the surrounding cells was observed [19, 21]. Moreover, recent data support the hypothesis that the second messenger c-di-GMP (bis-(3′5’)-cyclic-dimeric-guanosine monophosphate) plays a role in controlling the chemotactic response and, hence, biofilm formation in *Azospirillum* [24–26].

In several bacteria, c-di-GMP plays an important role in regulating the transition of the cells between a motile state and a sessile biofilm state. In addition, c-di-GMP is relevant in other bacterial functions, such as motility, chemotaxis, capsular polysaccharide formation, and cellulose synthesis [27, 28]. The synthesis and degradation of c-di-GMP are coordinated by the opposing activities of diguanylate cyclases (DGCs), which contain the GGDEF domain, and phosphodiesterases (PDEs), which harbor EAL or HD-GYP domains [28]. The GGDEF, EAL and HD-GYP domains were the first c-di-GMP modules to be identified using bioinformatics analyses, and these domains are widely distributed in bacterial genomes [28–30]. Hybrid proteins that contain both GGDEF and EAL domains have also been identified. In addition, other domains are often present, including sensory and regulatory modules, such as Per/Arnt/Sim (PAS), GAF, HAMP, REC, and MHTY, that modulate their enzymatic activities in response to external stimuli [28, 31]. The identification and functional characterization of c-di-GMP-associated proteins have indicated that the downstream signaling mechanisms of the c-di-GMP pathway might be versatile [32].

Various in silico analyses of proteins have demonstrated in that such studies can contribute to the identification to the function of a specific protein; these analyses have been performed by using a growing number of bioinformatics resources [33]. This work reports an analysis performed on the genomes of nine *Azospirillum* strains: *A. brasilense* Sp245, *A. brasilense* Sp7, *A. brasilense* Az39, *A. lipoferum* 4B, *Azospirillum* sp. B510, and the recently sequenced genomes of *A. thiophilum* BV-S^T^, *A. halopraeferens* DSM 3675, *A. oryzae* A2P, and *A. humicireducens* SgZ-5^T^. We focused on identifying the genes encoding proteins containing the GG[D/E]EF, EAL and GG[D/E]EF-EAL domains and on characterizing the associated sensing and signaling domains. This work will contribute to our understanding of this important family of proteins that regulate cellular levels of c-di-GMP in *Azospirillum.*

## Methods

We constructed the repertoire of genes coding for GG[D/E]EF, EAL and GG[D/E]EF-EAL domain proteins by analyzing nine *Azospirillum* genomes. The accession numbers of the chromosome and the other replicons in the genome of these strains are listed in Table 1.

**Table 1:** Accession numbers of the genomes of the *Azospirillum* sp., strains used in this study and the predicted modular signaling proteins involved in c-di-GMP metabolism

Strain	Chromosome Size (Mbs) ^a^CDS ^b^DGC/PDE/Hybrid	p1-Size (Mbs) CDS DGC/PDE/Hybrid	p2 Size (Mbs) CDS DGC/PDE/Hybrid	p3- Size (Mbs) CDS DGC/PDE/Hybrid	p4- Size (Mbs) CDS DGC/PDE/Hybrid	p5-Size (Mbs) CDS DGC/PDE/Hybrid	p6- Size (Mbs) CDS DGC/PDE/Hybrid	p7- Size (Mbs) CDS DGC/PDE/Hybrid	^c^Total Number	R
*A. bra*. Sp245	NC_016617.1 3.01 2, 690 10/3/6	NC_016594.1 1.76 1748 7/0/2	NC_016618.1 0.912 884 1/1/0	NC_016595.1 0.778 808 1/070	NC_016596.1 0.690 671 1/1/2	NC_016619 0.191 162 -	NC_016597.1 0.167 125 -	–	35	6
*A. bra.* Sp7	NZ_CP012914 3.01 2690 10/3/5	NZ_CP012915.1 1.75 629 7/0/2	NZ_CP012916.1 0.819 627 1/0/0	NZ_CP012917.1 0.645 555 1/1/2	NZ_CP012918.1 0.206 171 -	NZ_CP012919.1 0.159 110 -	_	_	34	7
*A. bra.* Az39	NZ_CP007793.1 3.06 2767 10/3/6	NZ_CP007794.1 1.9 1610 7/1/2	NZ_CP007795.1 0.933 754 1/0/0	NZ_CP007796.1 0.686 533 -	NZ_CP007797.1 0.641 558 1/1/2	NZ_CP007798.1 0.163 107 -	_	_	35	8
*A. lip* 4B	NC_016622.1 2.99 2693 11/4/10	NC_016585.1 1.04 867 1/0/0	NC_016586.1 0.750 619 2/0/2	NC_016623.1 0.648 515 3/1/2	NC_016587.1 0.645 555 1/0/1	NC_016624.1 0.478 384 1/0/1	NC_016588.1 0.295 220 -	–	40	6
*Azo* B510	NC_013854.1 3.3 2994 13/4/9	NC_013855.1 1.46 1127 2/0/4	NC_013856.1 0.723 613 1/0/0	NC_013857.1 0.681 532 3/1/2	NC_013858.1 0.628 536 0/0/1	NC_013859.1 0.537 422 0/0/1	NC_013860.1 0.261 181 -	–	41	9
*A. thio* BV-S	NZ_CP012401.1 3.04 17/4/9	NZ_CP012402.1 1.35 5/1/1	NZ_CP012403.1 0.879 1/0/0	NZ_CP012404.1 0.712 0/0/2	NZ_CP012405.1 0518 –	NZ_CP012406.1 0.694 0/0/1	NZ_CP012407.1 0.330 –	NZ_CP012408.1 0.083 –	35	10–11
*A. halo* DSM3675	NZ_AUCF00000000.1 5.26 4680	–	–	–	–	–	– –	–	38	12
*A. oryzae* A2P	NZ_FXAK00000000.1 7.67 6669	–	–	–	–	–	–	–	40	14
*A. humi* SgZ-5	NZ_CP015285.1 3.18 2846	–	–	–	–	–	–	–	29	13

*A.bra* = *A. brasilense, A. lip* = *A lipoferum*, *Azo* = *Azospirillum* B510, *A*. *thiophilum* BV-S, *A. halopraeferens* DMS3675, *A. oryzae* A2P, and *A. humicireducens* SgZ.5

*Chr* Chromosome, *p1* plasmid 1, *p2* plasmid 2, *p3* plasmid 3, *p4* plasmid 4, *p5* plasmid 5, *p6* plasmid 6. – no plasmid. *R* references

^a^Total number of protein-coding genes

^b^Number of GGDEF, EAL and hybrids proteins per replicon

^c^Total number of GGDEF, EAL and hybrids proteins per genome

The genomes were analyzed by performing BLAST searches of the GenBank database of the National Center for Biotechnology Information, with the Rapid annotation using subsystems technology (RAST) server [34], PFAM [35], SMART [36], PROSITE [37], conserved domain database [38], and MiST2.2 [39], websites to search for protein sequences from the Sp245, Sp7, Az39, 4B, B510, BV-S^T^, DMS3675, A2P and SgZ-5^T^ genome sequences. The amino acids of the motifs present in the various domains were identified using conserved domain database [38]. Clustal Omega was used to generate multiple protein sequence alignments [40, 41]. The localization of the signaling motifs was identified using the transmembrane helices in proteins (TMHMM) server to predict transmembrane helices [42]. All programs were used by following the specified parameters for successful analysis. The platform I-Tasser web server was used for automated protein structure and function predictions [43, 44]. Comparisons of the protein sequence domains were performed using the well-characterized homologous protein PleD of *Caulobacter crescentus* as a reference for the amino acid motifs of the GGDEF protein domain [45] and RocR from *Pseudomonas aeruginosa* as a reference for the EAL containing domain [46]. The crystal structures of a DGC (WspR) from *P. aeruginosa* [47] and the EAL domain of *C. crescentus* were used for the structural analyses [48] as suggested by the server and the I-Tasser parameters were run [49, 50]. In this study, models exhibited a higher C-scores (better model) were used, and models analyzed against the structure with higher resolution were used to create the corresponding model. The analysis was conducted and figures were made using the Chimera UCSF program [49] and VDM [50].

## Results

### Number and features of domains identified in the translated products from genes encoding predicted DGC and PDE proteins

A search for genes encoding enzymes involved in c-di-GMP metabolism was performed in the genomes of three strains of *A. brasilense* (Sp245, Sp7 and Az39) and in the genomes of *A. lipoferum* 4B, *Azospirillum* ssp. B510, *A. thiophilum* BV-S^T^, *A. halopraeferens* DMS3675, *A. oryzae* A2P, and *A. humicireducens* Sg-Z-5^T^ (Table 1). Some of these strains have composite genomes, containing several large plasmid-type replicons designated chromids [51] (because they contain essential genes), in addition to the chromosome (the largest replicon). A systematic analysis and comparison of the 9 genomes (Table 1) was performed as described in the methods section to identify the putative translated products that have diguanylate cyclase (DCG) and phosphodiesterase (PDE) activities and to define the amino acid motifs or signatures involved in catalytic activity, allosteric inhibition and interaction with metals (magnesium or manganese). This survey led to identification of three enzymatic classes of predicted proteins: DGCs, PDEs and hybrid DGC-PDEs. Indeed, even though the GGDEF and EAL domain-containing proteins have opposing activities, these two domains are often found coupled in the same proteins, which are referred to as hybrid proteins because they carry both domains. This nomenclature agrees with the presence of conserved amino acid motifs and with the distinctive secondary structure topology of these proteins [52]. The survey results led to the construction of a catalog of proteins predicted to be involved in c-di-GMP metabolism, as shown in Table 1. The number of genes encoding GGDEF, EAL, and hybrid domain proteins in each genome are as follows: *A. humicireducens*, 29 genes; *A. brasilense* Sp7, 34 genes; *A. brasilense* Sp245, Az39, and *A. thiophilum*, 35 genes each; *A. halopraeferens* 38 genes, *A. lipoferum* 4B and *A. thiophilum* 40 genes each; and *Azospirillum* ssp. B510 41 genes (Tables 1 and 2).

**Table 2:** Frequency of occurrence of genes encoding GGDEF, EAL, and hybrid domain proteins in the genomes of *Azospirillum* strains

DOMAIN #	*A.brasilense* 245	*A.brasilense* Sp7	*A.brasilense* Az39	*A.lipoferum* 4B	*Azospirillum* B510	*A.thiophilum* BV-S^T^	*A.halopraeferens* DMS3675	*A.oryzae* A2P	*A.humicireducens* Sg-Z-5
GGDEF	20	20	20	19	19	17	20	19	14
EAL	5	5	5	5	5	4	4	4	4
Hybrid	10	9	10	16	17	13	14	17	11
Total	35	34	35	40	41	34	38	40	29

Data extracted from http://blast.ncbi.nlm.nih.gov/Blast.cgi?PAGE=Proteins http://, SMART data base http://smart.embl-heidelberg.de/

The relative proportions of enzymes from each of the three classes, is shown in Table 2. *Azospirillum* genomes encode for 14 to 20 DGCs enzymes, which contain the GGDEF domain (Table 2 and Additional file 1: Table S1). Four PDE enzymes, which contain the EAL domains, are present in *A. brasilense* Sp7, *A. halopraeferens*, *A. oryzae*, and *A. humicireducens*, whereas five PDE enzymes are found in the other strains (Table 2 and Additional files 1, 2 and 3: Tables S1, S2, S3, and S4). From the third class of enzymes, representing the hybrid proteins, 10 of these hybrid proteins are present in *A. brasilense* Sp245 and Az39 and nine in Sp7. The number of hybrid domain proteins is larger in the other six genomes, which contain 11 to 17 (Table 2 and Additional files 1 and 2: Tables: S2, S3, and S4). There is a core set of fifteen genes that are completely conserved among all nine *Azospirillum* strains; these genes encode for the following proteins: (i) eight DGCs, which belong to the PleD, WspR, or CdgA families [26] and seem to be functionally important independent of host-related or environmental specializations, in the *Azospirillum* strains, (ii) three PDEs, which include ChsA, a protein that was functionally characterized as being involved in chemotaxis and aerotaxis [25, 53]; and (iii) four highly conserved GGDEF-EAL hybrid proteins (Table 3 and Additional file 1: Table S1). The proteins of each class differ in their lengths, architectures, sensor domains and cellular localization, which were predicted by structural analysis (Table 3 and Additional file 3: Table S3). Almost all of the proteins harbor accessory domains at their N-termini, with a few exceptions that will be described later in the manuscript. A similar diversity in the number of proteins and architecture has also been shown in other bacterial genomes [54, 55]. In particular, as observed in the case of *Azospirillum*, proteins encoded by the genomes as containing EAL domains were less frequent than proteins encoded as containing GGDEF domains [54–56].

**Table 3:**
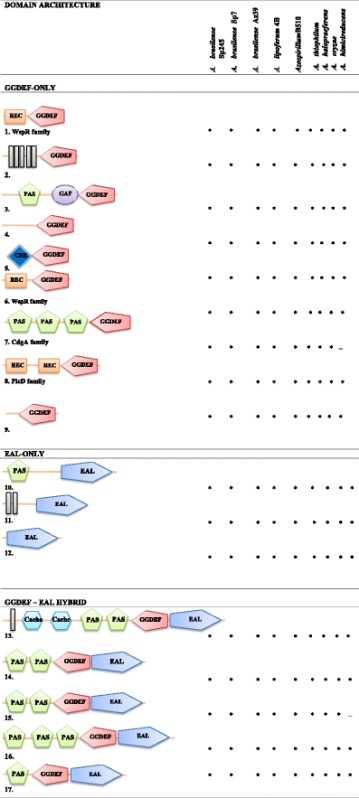
List of the highly conserved GGDEF, EAL and GGDEF-EAL predicted proteins. The organization and domain architectures found in the sequenced *Azospirillum* genomes

Conserved predicted proteins containing GGDEF, EAL and GGDEF-EAL domains from all analyzed the genomes. Schematic representation of domain organization of proteins found in the genomes. The domain prediction was performed based on protein sequences derived from the genome sequences of CdgA (diguanylate cyclase A) and ChsA (phosphodiesterase) from the *A. brasilense* Sp7 strain using the simple modular architecture research tool (SMART) program. The GGDEF domains are shown in rose, and the EAL domains are shown in blue. The sensor domains predicted by SMART are shown as follows: PAS/PAC, represented the PAS-fold family (green); transmembrane domains, TMD (gray); CACHE 2, calcium channels and chemotaxis receptor family (pale blue); REC, response regulator receiver (blue). Data were extracted from http://blast.ncbi.nlm.nih.gov/Blast.cgi?PAGE=Proteins: The website is indicated for each protein with its accession number according to BLASTP shown in Additional file 1: Table S1.

The subgroup of hybrid proteins containing tandem GGDEF/EAL or EAL/GGDEF domains are classified as DGCs, PDEs or DGC/PDEs (hybrid proteins) depending on the degree of conservation of the critical amino acid residues in their signature domains [28, 45, 46]. It was observed that all *Azospirillum* genomes have predominantly two types of hybrid proteins (Table 3 and Additional files 3 and 4: Tables: S3 and S5). In the first type of hybrid protein, the domains comprise highly conserved amino acid sequences suggesting that these hybrid proteins may exhibit both DGC and PDE activities. Because hybrid proteins also contain a regulatory partner or a sensor domain, these proteins require a mechanism by which to modulate their opposing activities. The associated sensor domain might determine the balance between the dual enzymatic activities via internal or extracellular signaling, as previously described for DcpA, a DGC/PDE protein from *Agrobacterium tumefaciens* that regulates attachment and biofilm formation. BphGL is a photoreceptor from *Rhodobacter sphaeroides* that is capable of both c-di-GMP synthesis and hydrolysis, and MucR is a protein from *P. aeruginosa* that has dual activity and is involved in alginate biosynthesis via c-di-GMP signaling [57–59].

In contrast, there were hybrid proteins in each *Azospirillum* genome that belonged to the second type of hybrid protein and were predicted to be enzymatically inactive with a “highly degenerate” GGDEF domain (Additional file 2: Table S2). However, the EAL domains of these proteins contain all of the amino acid motifs for PDE activity and are highly conserved [28, 45, 46], which suggests that these proteins correspond to PDEs that are catalytically active. This second type of hybrid proteins, with only a conserved EAL catalytic site, usually also has a signal-sensing partner domain, suggesting distinct modes for the regulation of PDE activity under different contexts, as shown in Table 3 (Additional files 2, 3 and 4: Tables: S2, S3, and S5) and as previously reported [60, 61].

### Genomic relatedness between *Azospirillum* strains

**Fig. 1:**
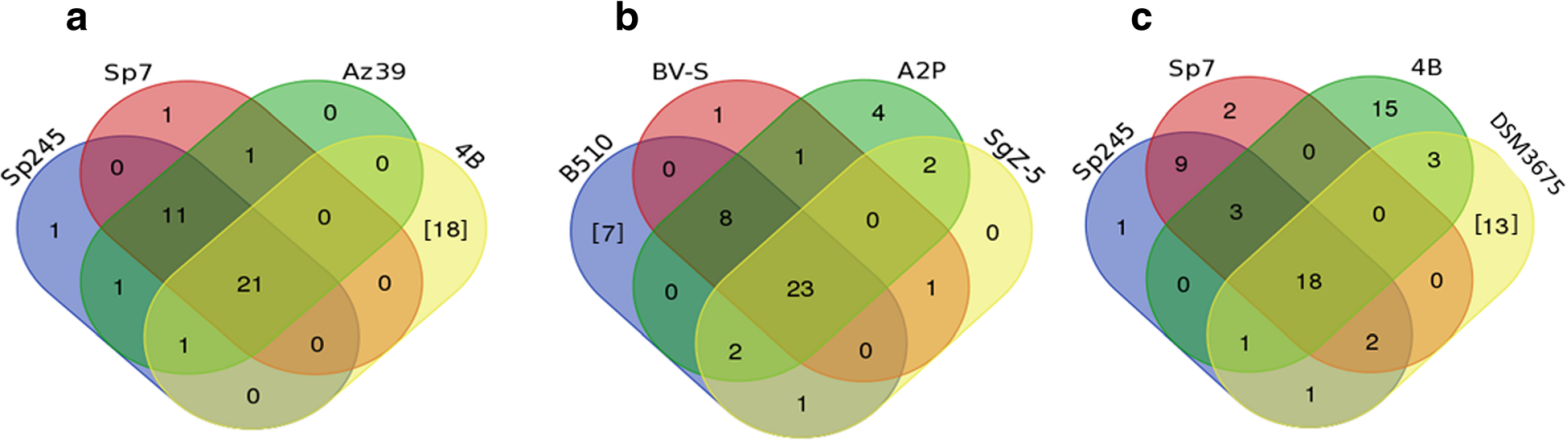
Venn diagrams showing the distribution of families predicted to be involved in c-di-GMP metabolism from the genomes of *Azospirillum* strains. **a** *A. brasilense* Sp245, Sp7, and Az39 and *A. lipoferum* 4B. **b**. *Azospirillum* sp. B510, *A. thiophilum* BVS-^T^, *A. oryzae* A2P, and *A. humicireducens* SgZ-5^T^. **c**. *A. brasilense* Sp245, Sp7, *A. lipoferum* 4B, and *A. halopraeferens* DSM3675. Numbers in black indicate the number of protein families; numbers in parentheses refer to the number of unique proteins in each genome that not found in the other genomes

Next, we performed a Venn diagram analysis in which each circle contained the memberships of the compared genomes. The relationships between all the putative proteins involved in c-di-GMP metabolism were assessed by considering whether they were conserved or were restricted to only one genome. The *A. brasilense* Sp245, Sp7 and Az39 genomes were compared with the genome of *A. lipoferum* 4B; 21 proteins were shared, but 18 proteins were indicated to be unique to the 4B genome (Fig. 1a). The B510 genome was compared with the genomes of the *A. humicireducens*, *A. thiophilum* and *A. oryzae* strains, and 23 proteins were conserved in all four genomes (Fig. 1b); only seven proteins were unique to the B510 genome, indicating that these genomes were the most closely related. Only 18 proteins were conserved in among the genomes of *A. brasilense* Sp245, Sp7, *A. lipoferum* 4B, and *A. halopraeferens*, and 13 proteins were found to be unique to the genome of *A. halopraeferens* (Fig. 1c). As these proteins are involved in signaling, these finding suggests that *Azospirillum* have evolved diverse transduction pathways, allowing better adaptation to a given niche.

### Sensory and regulatory module domains identified in GGDEF, EAL, and hybrid proteins

We further described the domain architectures found in each of the aforementioned sensory signaling domains identified in the GGDEF, EAL, and hybrid proteins; most of the proteins contained at least one predicted sensory domain, as shown in Table 3 (Additional files 2, 3 and 4: Tables: S2, S3 and S5). However, we found some exceptions, i.e., one to three putative GGDEF proteins, two EAL proteins, and one to two predicted GGDEF-EAL proteins were identified in all of the analyzed genomes. Other predicted proteins were seen to have at least one sensory domain. These regulatory or sensory domains detect small molecules, such as redox potential molecules, oxygen, nitric oxide (NO), light, voltage, osmolarity, and nutrients, and are also involved in protein-protein interactions. These domains enable the bacterium to integrate various types of input signals to establish a coordinated cellular output. In addition, all of these domains have regulatory functions that modulate the enzymatic activities of DGCs and PDEs in response to diverse environmental stimuli [28–32, 54, 55, 62, 63]. Indeed, the REC domain, which is a regulatory domain belonging to the CheY-like superfamily, has been identified as a receiver (phosphor-acceptor) domain or module that regulates the output of DGCs that responds to extracellular or intracellular signals transduced by their cognate sensor histidine kinases. The REC domain can be used to determine activity because its drives GGDEF dimerization, a process that is essential for activity and for cellular localization of the protein [28, 45, 64–67]. In addition, a careful examination of at least three databases revealed that some proteins were predicted to have transmembrane domains (TMD) and a peptide signal (signal P). These protein topologies suggested that the putative proteins are anchored to a membrane (Table 3 and Additional files 3 and 4: Tables: S3 and S5).

The PAS domain appears to be prevalent in proteins involved in c-di-GMP synthesis and degradation. In effect, as many as 38 predicted proteins contain one PAS/PAC domain or two or three domains in tandem, as is shown in Tables 3, 3S, and 5S. This domain is the most abundant sensory module found in signal transduction proteins throughout the bacterial kingdom; this domain generally binds small molecules and is the largest superfamily among domains solely dedicated to signal transduction [68]. Moreover, PAS/PAC domains are also involved in the protein-protein interactions that lead to dimerization, which is usually essential for DGC activity [26, 28, 54, 55, 64–66]. In addition, PDE enzymes also often form dimers and tetramers, which are required for activity [28, 60, 69]. Thus, if the *Azospirillum* genes encoding DGCs or PDEs are expressed, they may be subjected to different environmental or intracellular signals associated with PAS/PAC domains.

Bioinformatic analysis also identified HAMP domains in some of the DGCs and DGC/PDEs in all *Azospirillum* species (Table 3, Additional files 3 and 4: Tables: S3 and S5). These domains are defined as connectors between the periplasmic and cytoplasmic spaces. In addition, these domains transmit environmental stimuli across cytoplasmic membranes, and the conversion of that information to a signal triggers a change in function [70]. HAMP domains can be found in DGCs and DGC-PDEs that also have a TMD domain, suggesting that they are anchored to a membrane (Additional files 3 and 4: Tables: S3 and S5).

### Analysis of features found in selected predicted proteins that are potentially involved in c-di-GMP signaling

In our previous work, we identified the *chsA* gene, which encodes a PDE protein named ChsA that is involved in aerotaxis and chemotaxis [25, 53]. Herein, we found that the *chsA* gene is highly conserved in the genomes of all *Azospirillum* species (Table 3, Additional file 2: Table: S2), showing a 99% to 51% similarity in amino acid residues in relation to *A. brasilense* Sp245. In addition, the DGC named CdgA, which is encoded by the ID: WP_035674663 gene in *A. brasilense* Sp7, was demonstrated to be involved in biofilm formation [26]. In this study, we found that *cgdA* is well conserved in the analyzed genomes (Table 3) and shares considerable identity (from 98 to 60%) with proteins from *A. brasilense* Sp245.

An interesting feature that was observed in the predicted DGCs and hybrid proteins from all nine genome sequences of the *Azospirillum* species is that they include both CACHE and TMDs in tandem domains (Table 3, and Additional files 3 and 4: Tables S3 and S4). The CACHE (calcium channels and chemotaxis receptors) domain is an extracellular sensor domain that is present in bacteria and detects extracellular signals, such as small molecules and nutrients, and this domain is a ligand-binding domain commonly found in bacterial chemoreceptors [71]. These domains have been identified exclusively in proteins that contain output signaling domains, such as the DGC and PDE signal transduction proteins [71]. The majority of known ligands for the dCache_1 domain are amino acid sensors [72], whereas many of the single CACHE domains bind organic acids [73].

Only one highly conserved putative EAL-GGDEF hybrid protein was found in the studied genomes, and this protein had an EAL domain at the N-terminus (Additional files 3 and 4: Tables: S3 and S5). Both the EAL and the GGDEF domains are highly conserved, suggesting that these proteins might exhibit both activities depending on internal cellular signaling.

Several predicted DGCs and hybrid proteins contained both CHASE and TMDs domains at the N-termini (Additional files 3 and 4: Tables S3: and S5). The CHASE domain is a sensory domain named so because it is found in cyclases/histidine kinases with sensory functions. The CHASE domain is predicted to be a periplasmic domain consisting of 362 amino acid residues that serves as a transmembrane receptor and is often found in bacteria and plants. CHASE domains bind small molecules such as peptides and the phytohormone cytokinin [74]. As a bacterium associated with plants, *Azospirillum* might use this protein as a chemotaxis receptor.

The predicted hybrid protein (GenBank: Sp245, WP_014199675; Sp7, WP_059399655, and Az39, WP_040138308) found in the *A. brasilense* genomes but not in other genomes showed an interesting structural architecture (Additional file 3: Table S3). This protein possesses an MHYT domain encompassing seven TMDs that are all localized at the N-terminus and has an MHYT motif consisting of four conserved amino acid residues (methionine, histidine, tyrosine, and threonine) that are predicted to be located near the outer face of the inner membrane. It has been suggested that the MHYT domain serves as a sensing domain [31]. The membrane topology of the MHYT domain indicates that the conserved residues of this domain can coordinate one or two copper ions, suggesting that this domain plays a role in sensing oxygen, CO or NO. In addition, the C- terminus includes PAS-GGDEF-EAL domains. The gene encoding this protein is often fused to a LysR-type DNA-binding helix-turn-helix protein, and an investigation of the genome organization showed that the genomic position of this protein was conserved in the genomes of all the *A. brasilense* strains [31, 39]. In *P. aeruginosa,* alginate biosynthesis, formation of highly structured biofilms, and inhibition of swarming motility are regulated by MucR, which is a hybrid MHYT-DGC-PDE protein [75].

### Structural and tridimensional topographies

#### Structural features of the GGDEF and EAL domains of the DGCs, PDEs and hybrids proteins from the *A. brasilense* Sp7 genome

Next, we determined that the structural features of the predicted proteins found in the *A. brasilense* Sp7 genome confirm previous predictions. All models of the GGDEF, EAL or GGEDF/EAL proteins had C-scores higher than 0.49 (better models had values close to 2) [43, 44]. The C-values of all models are reported in Table 4.

**Table 4:** C-score of the predicted structures of EAL and GGDEF proteins

GGDEF only	C score	EAL only	C score	GGDEF hybrid proteins	C score	EAL hybrid proteins	C score
WP_059399097	0.79	WP_059398606	1.53	WP_079285130	1.25	WP_059399067	0.61
WP_051140034	1.53	WP_051140161	1.61	WP_051140186	1.33	WP_079285130	1.83
WP_035675850	0.58	WP_035672792	1.08	WP_051140104	1.28	WP_051140186	1.80
WP_035672942	0.60	WP_035682417	1.83	WP_059398931	1.18	WP_051140104	1.78
WP_035674663	0.58	CAJ18244	1.60	WP_059399067	0.68	WP_059398931	1.73
WP_035674304	0.59			WP_051140628	1.24	WP_051140628	1.82
WP_035671267	0.59			WP_035678503	1.27	WP_035678503	1.65
WP_035671094	0.49			WP_059399655	1.27	WP_059399655	1.72
WP_035671042	1.08			WP_059399677	1.29	WP_059399677	1.71
WP_035670844	0.59						
WP_035670654	0.79						
WP_035676633	0.61						
WP_035671246	0.73						
WP_051140383	0.81						
WP_035678542	0.88						
WP_059399331	0.52						
WP_059399449	1.15						
WP_035682812	0.96						
WP_079285367	0.54						
WP_051140397	0.77						

The data were extracted from the I-TASSER server for protein structure and function. The website is indicated for each gene with is accession number according to BLASTP

#### GGDEF-only DGCs and hybrid (GGDEF domain) proteins

**Fig. 2:**
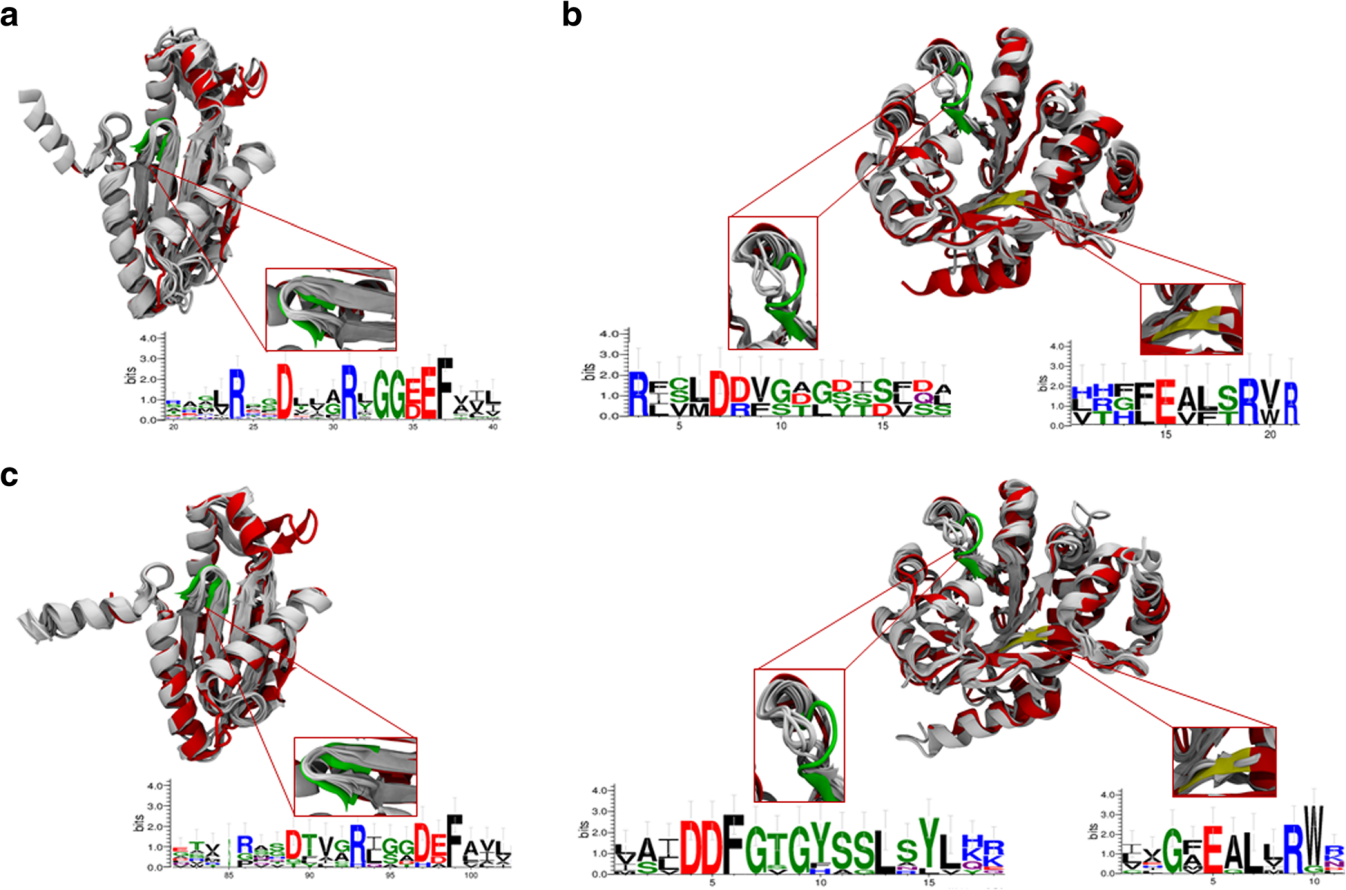
Models and structural features of GGDEF, EAL and hybrid proteins from the *A. brasilense* Sp7 genome. **a** Diguanylate cyclase domain structures from the *A. brasilense* Sp7 genome. The structures shown correspond to the 20 conserved DGCs sequences indicated in Table 4 in comparison to the crystal structure of WspR (PDB id: 3BRE; red) from *P. aeruginosa* [47]. The domain surface representations are labeled in white, and the bound ligand is labeled in green. The WebLogo sequence [78] is based on the PFAM alignments, which show conserved GG[D/E]F motifs and are colored green-red. **b**. Domain structures of phosphodiesterase from the *A. brasilense* Sp7 genome. The structures shown correspond to the five conserved PDE-EALs indicated in Table 4 in comparison to the crystal structure of PdeA (PDB ID: 3U2E; red) from *C. crescentus* [48]. The domain surface representations are labeled in white, the bound ligands (EAL domain) are shown in yellow, and loop 6 is colored green. The WebLogo sequence [78] is based on the PFAM alignments, which show a conserved motif. The red arrow indicates the visible discrepancies in the 3U2E crystallographic structure. **c**. Structures of the hybrid proteins from the *A. brasilense* Sp7 genome. The structures shown correspond to the nine hybrid sequences indicated in Table 4 in comparison to the crystal structure of WspR (PDB id: 3BRE; red) from *P. aeruginosa* [47]. The domain surface representations are labeled in white, and the bound ligand is labeled in green. The WebLogo sequence [78] is based on the PFAM alignments, which show a conserved GG[D/E]F motifs and are colored green-red. The structures shown correspond to the nine conserved PDE-EALs indicated in Table 4 in comparison to the crystal structure of PdeA (PDB ID: 3U2E; red) from *C. crescentus* [48]. The domain surface representations are labeled in white, the bound ligands (EAL domain) are shown in yellow, and loop 6 is colored green. The WebLogo sequence [78] is based on the PFAM alignments, which show a conserved EAL motif. The red arrow indicates the visible discrepancies in the 3U2E crystallographic structure

The GGDEF protein models were constructed based on the crystal structure of the GGDEF conserved domain of WspR (PDB id: 3BRE), which has a crystallographic resolution of 2.4 Å [47]. Structural alignments, shown in Fig. 2a, were performed using the GGDEF domain of 3BRE from amino acids L170 to Q339. Even though the putative DGC protein (GG[DE]EF-only domain) sequences analyzed did not possess a highly identity percentages (ranging from 30.41 to 48.02%) (Additional file 5: Table: S7a), the proteins contained all the essential conserved amino acid residues that bind the substrate, GTP, to have enzymatic activity [76, 77]. The Web Logo [78] alignment in Fig. 2a shows the characteristic secondary structure elements of the DGCs, such as five α helices and seven short β strands (α_1_β_1_α_2_α_3_β_2_β_3_α_4_β_4_β_5_β_6_α_5_β_7_) [52]. In addition, regions of the sequence were identified with high root main square deviation (RMSD) values, namely, R195 to L203, C240 to L246, P260 to P264, and T275 to F312, as shown in Additional file 5: Table: S7a. High RMSD values were found for most of these regions except for T275 to F312, which corresponded to the loop regions shown in Fig. 2a. The region from T275 to F312 corresponded to a loop region and a β strand that crossed from one side to the other in each of the protein models. In this region, the models exhibited a secondary structure. We suggested that the absence of a secondary structure may be the result of protein model construction and that these structures must be studied by circular dichroism spectroscopy or dynamic simulations in further studies.

**Fig. 3:**
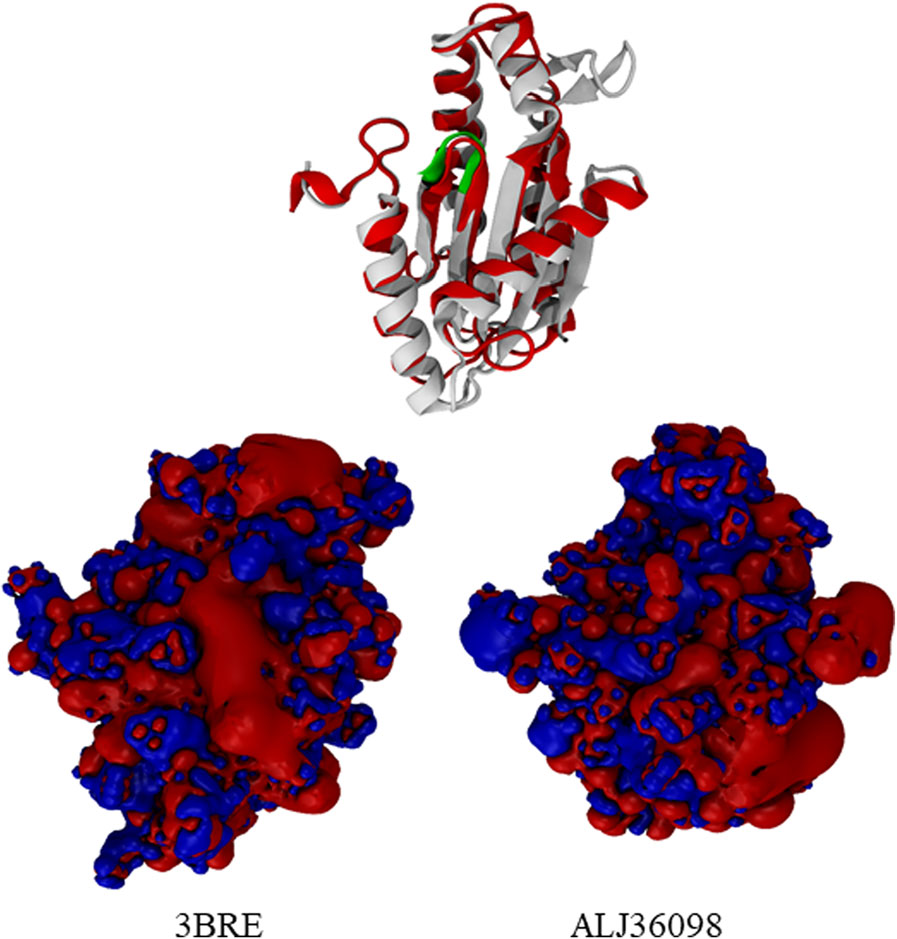
Structure of ALJ36098 from the *A. brasilense* Sp7 genome. **a** The model superimposed with WspR (PDB id: 3BRE) from *P. aeruginosa* [47]. The 3BRE structure is colored red. The GGDEF motif is colored green, and the SDHAF motif is shown superimposed. **b** The electrostatic potential (+/˗ 4kT/e) calculated with the APBS of both proteins in the same orientation, as indicated above (**a**). The positive amino acid residues are colored blue, and the negative amino acid residues are colored red. The potential difference in the binding site changes in ALJ36098

The GGDEF hybrid proteins had a sequence conservation percentages ranging from 24.11 to 33.14% (Additional file 5: Table: S7b). As indicated by the high RMSD values, these regions corresponded to sequence gaps or insertions, primarily from R195 to Q202 and S280 to L294. The ALJ36098 protein (GenBank ID: WP_059398931) was found to have the most different sequence in the characteristic of GGDEF motif (Additional file 2: Table: S2 b3). Indeed, ALJ36098 had an SDHAF motif; this variation is divergent in the sizes and charges of the amino acid residues involved in enzymatic activity, suggesting that this predicted protein lacks catalytic activity [64–66, 76, 77]. Additionally, an electrostatic potential analysis mapped on the protein surfaces showed that the SDHAF motif changed the charge distribution. These changes may confer on the protein a different affinity for its ligand compared to the affinity of GGDEF proteins (Fig. 3) [76, 77].

### EAL-only PDEs and hybrid (EAL domain) proteins

#### EAL-only proteins

EAL proteins from the *A. brasilense* Sp7 genome were compared to the crystal structure of the EAL domain of PdeA from *C. crescentus* (PDB ID: 3U2E), which has a crystallographic resolution of 2.32 Å [48]. When compared to 3U2E, the EAL-only proteins had lower sequence conservation, ranging from 19.50 to 36.44%, than the EAL hybrid proteins. (Additional file 5: Table: S7c). As indicated in Fig. 1b, the regions with high RMSD-backbone values are located in different sections across the structures, e. g, the first ten amino acids at the amino terminus; these regions are A327 to G350, W369 to P393 and R464 to V501. As indicated in Fig. 1b, some loops from the EAL-only proteins with visible discrepancies were included in the comparison to the crystallographic structure. However, as shown in Fig. 2b, the crystal structures of the EAL domains show that the proteins possess the conserved signature motif (EAL) in addition to the flexible loop (“loop 6”), which has been extensively characterized in (β/α) barrel proteins, and that both of these features are required for catalytic activity in a functional protein [60, 69, 76, 79, 80].

#### EAL-hybrid proteins

The sequence conservation percentages for these proteins ranged from 29.02 to 43.20%. The lowest conservation percentage corresponded to ALJ36617 (GenBank ID: WP_059399067) and the highest to ALJ36098 (GenBank ID: WP_059398931). Moreover, the low sequence conservation appeared not to be a factor for model prediction by I-Tasser. As shown in Fig. 2c, the structural alignment of 3U2E against all of the EAL hybrid proteins gave a low RMSD value for the backbone atoms. The amino acid residues, when present, had high RMSD values for residues from T368 to T380 (3U2E sequence numbering) (Additional file 5: Table: S7d). These regions presented greater sequence fluctuation, including gaps in some of the sequences; however, the amino acid residues involved in the ligand-metal binding of c-di-GMP and enzymatic activity are highly conserved in all sequences [48, 60, 76, 79–81] (Fig. 2c). In addition, residue M358 exhibited some changes, including-change from a methionine in 3U2E to hydrophobic residues (such as valine, leucine or alanine) in a majority of the analyzed sequences and to threonine in ALJ39102 (GenBank ID: WP_059399677).

## Discussion

It is well documented that in the bacterial kingdom c-di-GMP signaling is linked to biofilm formation and several other phenotypes that are important to the lifestyle of bacteria. We advanced our understanding of c-di-GMP signaling in the most important species of *Azospirillum*, e.g., those that are used as inoculants to promote plant growth or in soil bioremediation, by studying how many domain architectures and tridimensional structures were contained in the predicted proteins of genes encoding DCGs, PDEs and DGC-PDEs; these genes are widespread and are found in other environmental and soil bacteria. Indeed, approximately 29 to 41 genes encoding these modular signaling proteins were identified in the *Azospirillum* genomes, establishing that the distribution of this genes in *Azospirillum* is comparable to that in other bacteria from soil or marine environmental bacteria, such as *Sinorhizobium meliloti* [82], other species of *Rhizobium* [83], *Pseudomonas putida* [62], *Shewanella oneidensis* [63, 84], and *Burkholderia lata* SK875 [85], which reportedly encode a considerable number of proteins predicted to be involved in c-di-GMP metabolism.

The comparisons of the genomes showed that 15 proteins shared a significant percentage of identity at the amino acid residue level (the genes comprising the core), indicating the genetic relatedness among *Azospirillum* strains as previously described [6, 67, 86]. In addition, some of the genes were duplicated and identified at different genetic locations (chromosome or chromids) in the same genome, indicating that they might be derived from the duplication of a common ancestral gene that then diverged from the parent copy by mutation and selection, as proposed by the phylogenetic analysis. This evolution suggests that these genes were paralogs and that they were likely acquired by horizontal transfer (HGT), as defined for the *A. brasilense* and *A. lipoferum* genomes [6, 67, 86, 87]. Notably, it was observed these genomes possessed genes that encoded for ChsA, a PDE involved in chemotaxis and aerotaxis, and it has been well established by Russell et al. [25] that *Azospirillum* uses chemotaxis to navigate through the soil to find optimal surroundings for survival. Thus, the control of cellular motility by c-di-GMP signaling is the best illustration, to date, of the importance of a c-di-GMP-controlled rapid response to changing environmental conditions. Based on with phylogenic analysis, the genomes were clustered in three groups: strains of *A. brasilense* (Sp245, Sp7 and Az39) included in the same clade; strain *A. lipoferum* 4B, which clustered with *Azospirillum* B510, *A. humicireducens* SgZ-5^T^, *A. thiophilum* BV-S^T^, and *A. oryzae* A2P; and *A. halopraeferens* DSM3675, the genes of which were the most divergent. This is in agreement with previous studies on the whole-genomes of *Azospirillum* strains [6–14]. In addition, it was interesting to note that proteins encoded by the *A. halopraeferens* genome showed very complex structural features, as indicated in Table 5S (Additional file 4). *A. halopraeferens*, isolated from the rhizoplane of Kallar grass (*Leptochl oa fusca* L. Kunth), is a salt tolerant bacterium [88]. The bacterium was inoculated to an oilseed halophyte *Salicornia bigelovii* Torr plant in salt-contaminated, infertile areas, and under these detrimental stress conditions, the plant-growth-promotion was significantly improved [89]. Therefore, the data obtained here strongly suggest host specificity and environment-specific adaptation.

The domain architectures of the deduced amino acid sequences of DGC and PDE proteins from *Azospirillum* genomes were also predicted that to included diverse sensor domains, such as REC, PAS/PAC, CHASE, GAF, MHYT and CACHE, that are involved in activity regulation by driving the protein dimerization process, which is essential for activity, or by sensing small molecules commonly found in rhizospheric or aquatic habitats [28–32, 69, 90]. These predictions are useful to predict how bacteria are able to monitor the internal metabolic status of a cell as well sense environmental cues, such as those from root exudates or the rhizosphere, or signals associated with a particular environment [57–59, 62, 63, 83, 91].

Furthermore, the cellular localization of some proteins was assessed by the presence or absence of transmembrane helices; the cellular localization of these proteins might indicate that the cellular c-di-GMP pool is localized to support functional micro-compartmentalization, which may be participate in the response to different environmental signals or may allow membrane localization of the protein after a spatial signal is sensed, thereby regulating its enzymatic activity with the corresponding co-localization of DGC, as previously described in several studies [28, 57, 59, 65, 77].

The analysis of structural architecture has proven to be very informative with regard to putative functions of signalling proteins. It was mentioned that a majority of hybrid proteins have conserved EAL catalytic sites, and these predicted proteins contribute to the total PDE cellular activity. The inactive GGDEF domains of these proteins function by regulating hydrolytic activity, as previously described in *Xanthomonas oryzae* pv. *oryzicola* [91], or by acting as “trigger enzymes” with a dual function of either hydrolyzing c-di-GMP, or acting as an effector that binds to a transcriptional regulator that acts on a promotor involved in a signaling cascade. This signaling cascade controls matrix production in the biofilm; or can also control its own transcription, as described for PdeR and PdeL from *Escherichia coli* [92, 93]. This is the case for the ALJ36098 protein from *A. brasilense* Sp7 which is predicted to lack catalytic activity [76, 77]; however, this protein might exhibit regulatory or effector functions by binding c-di-GMP or GTP, as previously described for some hybrid proteins [70, 89–92], or may act as an effector in the c-di-GMP signaling cascade as previously described [92–94]. Considering the data presented here, we suggest that this structural analysis provides important information to predict the function of these proteins containing GGDEF, EAL, and hybrid domains, and creates a paradigm for future studies on the evolution of enzymes involved in c-di-GMP metabolism.

## Conclusions

In summary, compared to other plant-associated bacteria *Azospirillum* were found to contain a number of similar genes (29 to 41) encoding DGCs and PDEs in their genomes. Our findings help elucidate the functions of the predicted hybrid multi- domain proteins, which allow the bacteria to integrate different signals via significant signaling plasticity. Indeed, this significant flexibility might reflect differentially regulated c-di-GMP signaling mechanisms in *Azospirillum* that enable responses to distinct environmental and cellular signals. Therefore, in silico analysis of the ligand binding domains in the genomic sequences is a pre-requisite for further experimental characterization and evaluation of biological function. Thus, these conserved signaling proteins might be ecologically relevant and may explain how *Azospirillum* adapts to its specific ecological niche. An interesting question is raised regarding involvement of these proteins in physiological regulation. Future phenotypic and biochemical studies are needed to answer this question.

## Additional files

Additional file 1 **Table S1.** Accession numbers of GGDEF, EAL and hybrid proteins encoded by genes conserved in all analyzed *Azospirillum* genomes. Data extracted from http://blast.ncbi.nlm.nih.gov/Blast.cgi?PAGE=Proteins. (DOCX 24 kb)

Additional file 2 **Table S2.** Accession numbers and classifications of GGDEF, EAL and hybrid proteins predicted based on the conservation of signature motifs that were found in all analyzed genomes. Data extracted from http://blast.ncbi.nlm.nih.gov/Blast.cgi?PAGE=Proteins and http:// and http://smart.embl-heidelberg.de/ following the notation from Römling et al. (2017). (DOCX 109 kb)

Additional file 3 **Table S3** and **Table S4.** Table S3. Repertoire of GGDEF, EAL and GGDEF-EAL predicted proteins, organization, and domain architectures found in the selected *Azospirillum* spp. genomes. Table S4. Accession numbers of GGDEF, EAL and hybrid proteins encoded by genes of select the analyzed *Azospirillum* genomes. Data extracted from http://blast.ncbi.nlm.nih.gov/Blast.cgi?PAGE=Proteins and http:// and http://smart.embl-heidelberg.de/. (DOCX 268 kb)

Additional file 4 **Table S5** and **Table S6.** Table 5. Repertoire of GGDEF and GGDEF-EAL (hybrid) predicted proteins, organization, and domain architectures represented exclusively in the *A. halopraeferens, A. thiophilum*, and *A. oryzae* genomes. Table 6. Accession numbers of GGDEF and hybrid proteins encoded by genes exclusively found in *A. halopraeferens*, *A. thiophilum*, or *A. oryzae* genomes. Data extracted from http://blast.ncbi.nlm.nih.gov/Blast.cgi?PAGE=Proteins and http://smart.embl-heidelberg.de/. (DOCX 162 kb)

Additional file 5 **Table S7.** The alignments, root main square deviations (RMSDs) and sequence conservation percentages of proteins with GGD[E]EF, EAL and hybrid domains encoded by genes found in the *A. brasilense* Sp7 genome. Table 7Sa data including the GGD[E]EF proteins; Table 7Sb, data including the EAL proteins; Table 7Sc and 7Sd, data including the hybrid proteins. Data extracted from http://blast.ncbi.nlm.nih.gov/Blast.cgi?PAGE=Proteins. (DOCX 6071 kb)

## Abbreviations

BLAST: Basic local alignment search tool
c-di-GMP: Cyclic-di-GMP
DGCs: Diguanylate cyclases
MiST2.2: Microbial signal transduction database
PDEs: Phosphodiesterases
PFAM: Protein domain database
PGPB: Plant-growth-promoting bacteria
PROSITE: Protein domain database for functional characterization
RAST: Rapid annotation using subsystems technology
RMSD: Root main square deviations
SMART: Simple modular architecture research tool
VMD: Visual molecular dynamics
