# Supplementary material for: In silico comparative analysis of GGDEF and EAL domain signaling proteins from the Azospirillum genomes
Source: BMC Microbiol. 2018 Mar 9;18:20. doi: 10.1186/s12866-018-1157-0 (PMC5845226; doi:10.1186/s12866-018-1157-0)
Supplement: Supplementary file 1 — Table S1. Accession numbers of GGDEF, EAL and hybrid proteins encoded by genes conserved in all analyzed Azospirillum genomes. Data extracted from http://blast.ncbi.nlm.nih.gov/Blast.cgi?PAGE=Proteins. (DOCX 24 kb) [file 12866_2018_1157_MOESM1_ESM.docx]

**Additional material**

***In silico* comparative analysis of GGDEF and EAL domain signaling proteins from the *Azospirillum* spp genomes.**

Alberto Ramírez Mata ^1¶^, César Millán Pacheco ^2¶^, José Francisco Cruz Pérez^1^, and Beatriz E. Baca^1*^.

^1^ Centro de Investigaciones en Ciencias Microbiológicas, Benemérita Universidad Autónoma de Puebla. Edif. Edif. IC11, Ciudad Universitaria, Col. San Manuel Puebla Pue. CP72570 Puebla México.

^2^ Facultad de Farmacia. Universidad Autónoma del Estado de Morelos, Av. Universidad #1001, Col. Chamilpa, C.P. 62209. Morelos México.

**Additional file 1:**

**Table S1. Accession numbers of GGDEF, EAL and hybrid proteins encoded by genes conserved in all analyzed *Azospirillum* genomes.**

Data extracted from http://blast.ncbi.nlm.nih.gov/Blast.cgi?PAGE=Proteins

**Additional file 1**

**Table S1. Accession numbers of GGDEF, EAL and hybrid proteins encoded by genes conserved in all analyzed *Azospirillum* genomes.**

| **#** | ***A. brasilense* Sp245** | ***A. brasilense* Sp7** | ***A. brasilense* Az39** | ***A. lipoferum* 4B** | ***Azospirillum* B510** | ***A. thiophilum*** | ***A. halopraeferens*** | ***A.***  ***oryzae*** | ***A.***  ***humicireducens*** |
| --- | --- | --- | --- | --- | --- | --- | --- | --- | --- |
| **GGDEF DOMAINS** | | | | | | | | | |
| **1.** | **WP_014197311** | **WP_035671246** | **WP_040133993** | **WP_014248328** | **WP_042442983** | **WP_082108818** | **WP_029010874** | **WP_085089873** | **WP_063634969** |
| **2.** | **WP_014239030** | **WP_035670654** | **WP_038530041** | **WP_014249136** | **WP_012972667** | **WP_060721713** | **WP_029010818** | **WP_085086986** | **WP_063635604** |
| **3.** | **WP_014239318** | **WP_035671267** | **WP_038530483** | **WP_065814210** | **WP_012973137** | **WP_045581755** | **WP_084536514** | **WP_085087985** | **WP_063633816** |
| **4.** | **WP_014240625** | **WP_035674304** | **WP_038527442** | **WP_014247155** | **WP_012973551** | **WP_082108926** | **WP_051341118** | **WP_085088695** | **WP_063634076** |
| **5.** | **WP_014239247** | **WP_035671094** | **WP_038525904** | **WP_014248886** | **WP_012972950** | **WP_045581167** | **WP_029007913** | **WP_085087587** | **WP_063635416** |
| **6.** | **WP_014239228** | **WP_035671042** | **WP_038525874** | **WP_014248701** | **WP_012974989** | **WP_045582432** | **WP_029010314** | **WP_085090164** | **WP_063635255** |
| **7.** | **WP_014240775** | **WP_035674663** | **WP_038527678** | **WP_014189705** | **WP_012973965** | **WP_045582203** | **WP_029007170** | **WP_085089300** | **─** |
| **8.** | **WP_014197448** | **WP_035676633** | **WP_040134391** | **WP_014248101** | **WP_012974481** | **WP_045582086** | **WP_029007018** | **WP_085089772** | **WP_063634866** |
| **9.** | **WP_014197451** | **WP_079285367** | **WP_063922679** | **WP_014248103** | **WP_012974483** | **WP_045582085** | **WP_084536339** | **WP_085089774** | **WP_063634868** |
| **EAL DOMAINS** | | | | | | | | | |
| **10.** | **WP_014239906** | **WP_051140161** | **WP_051657894** | **WP_044550563** | **WP_012974361** | **WP_045580288** | **WP_084536631** | **WP_085089651** | **WP_063634607** |
| **11.** | **WP_014241505** | **WP_059398606** | **WP_038529539** | **WP_014248761** | **WP_012975037** | **WP_082108838** | **WP_035693714** | **WP_085090217** | **WP_063636223** |
| **12.** | **WP_014199814** | **WP_035682417** | **WP_040138014** | **WP_044549890** | **WP_012974608** | **WP_082108813** | **WP_029006916** | **WP_085089859** | **WP_063634954** |
| **HYBRID DOMAINS** | | | | | | | | | |
| **13.** | **WP_014239522** | **WP_051140104** | **WP_038531265** | **WP_014246975** | **WP_052293616** | **WP_045581881** | **WP_084536765** | **WP_085088261** | **WP_063633937** |
| **14.** | **WP_014198689** | **WP_051140628** | **WP_040134184** | **WP_014247398** | **WP_012973859** | **WP_045582112** | **WP_084536394** | **WP_085089560** | **WP_063634303** |
| **15.** | **WP_014199833** | **WP_059399677** | **WP_040138037** | **WP_014249492** | **WP_042445660** | **WP_045583101** | **WP_035694205** | **WP_085086601** | – |
| **16.** | **WP_014198291** | **WP_035678503** | **WP_040135003** | **WP_014248011** | **WP_042442896** | **WP_082108789** | **WP_029006814** | **WP_085089727** | **WP_063634828** |
| **17.** | **WP_014239353** | **WP_059398931** | **WP_038526104** | **WP_014248989** | **WP_012972789** | **WP_045581444** | **WP_029010954** | **WP_085090458** | **WP_063635491** |

Data extracted from http://blast.ncbi.nlm.nih.gov/Blast.cgi?PAGE=Proteins
