# Supplementary material for: In silico comparative analysis of GGDEF and EAL domain signaling proteins from the Azospirillum genomes
Source: BMC Microbiol. 2018 Mar 9;18:20. doi: 10.1186/s12866-018-1157-0 (PMC5845226; doi:10.1186/s12866-018-1157-0)
Supplement: Supplementary file 2 — Table S2. Accession numbers and classifications of GGDEF, EAL and hybrid proteins predicted based on the conservation of signature motifs that were found in all analyzed genomes. Data extracted from http://blast.ncbi.nlm.nih.gov/Blast.cgi?PAGE=Proteins and http:// and http://smart.embl-heidelberg.de/ following the notation from Römling et al. (2017). (DOCX 109 kb) [file 12866_2018_1157_MOESM2_ESM.docx]

**Additional material**

***In silico* comparative analysis of GGDEF and EAL domain signaling proteins from the *Azospirillum* spp genomes.**

Alberto Ramírez Mata ^1¶^, César Millán Pacheco ^2¶^, José Francisco Cruz Pérez^1^, Martha Minjárez Sáez ^1^, and Beatriz E. Baca^1*^.

^1^ Centro de Investigaciones en Ciencias Microbiológicas, Benemérita Universidad Autónoma de Puebla. Edif. Edif. IC11, Ciudad Universitaria, Col. San Manuel Puebla Pue. CP72570 Puebla México.

^2^ Facultad de Farmacia. Universidad Autónoma del Estado de Morelos, Av. Universidad #1001, Col. Chamilpa, C.P. 62209. Morelos México.

**Additional file 2: Additional file 2: Table 2S.** Accession numbers and classifications of GGDEF, EAL and hybrid proteins predicted based on the conservation of signature motifs that were found in all analyzed genomes.

**A.1. The GGDEF, A. 2 EAL, and A3. Hybrid domain proteins found in the *A. brasilense* Sp245 genome**

**B.1. The GGDEF, B. 2 EAL, and B3. Hybrid domain proteins found in the *A. brasilense* Sp7 genome**

**C.1. The GGDEF, C. 2 EAL, and C. Hybrid domain proteins found in the *A. brasilense* Az39 genome**

**D.1. The GGDEF, D2. EAL, and D3. Hybrid domain proteins found in the *A. lipoferum* 4B genome**

**E.1. The GGDEF, E2. EAL, and E3. Hybrid domain proteins found in the *Azospirillum* B510 genome**

**F.1. The GGDEF, F2. EAL, and F3. Hybrid domain proteins found in the *A. thiophilum* genome**

**G.1. The GGDEF, G2. EAL, and G3. Hybrid domain proteins found in the *A. halopraeferens* genome**

**H.1. The GGDEF, A2. EAL, and H3. Hybrid domain proteins found in the *A. oryzae* genome**

**I. 1. The GGDEF, I2. EAL, and I3. Hybrid domain proteins found in the *A. humicireducens* genome**

**Data extracted from** http://blast.ncbi.nlm.nih.gov/Blast.cgi?PAGE=Proteins and http:// and <http://smart.embl-heidelberg.de/> and following the notation from Römling et al. [1]. MBS 1 and MBS2 = metal binding sites in EAL domain; GMP-BS1 and GMP-BS2 = amino acid binding motifs to cyclic-diGMP; Loop = Loop6 from EAL domains. H2O BS, binding site to water; AA, number total of amino acid; xx = whichever amino acid. The domain prediction was performed based on protein sequences derived from the genome sequences of CdgA (Diguanylate cyclase A)[ ] and ChsA (Phosphodiesterase), both of them previously characterized in *A. brasilense* Sp7 strain using the modular architecture research tool (SMART) program. The sensor domains were predicted by SMART are shown as follows. PAS/PAC, represented as PAS fold family; Transmembrane domains, TMD; CACHE 2, Calcium channels and chemotaxis receptor family; REC, Response regulator receiver; HAMP “linker regions” Histidine kinases, Adenyl cyclases, Methyl-accepting chemotaxis proteins and Phosphatases. CHASE, Cyclases, Histidine kinases Associated Sensory Extracellular domain; GAF, cGMP phosphodiesterase, Adenyl cyclase; PBPb, Bacterial extracellular solute-binding proteins; Protoglobine, domain-associated heme.

**Additional file. 2**

**Table 2S.** Accession numbers and classifications of GGDEF, EAL and hybrid predicted proteins according to conservation of signature motifs shown in [1 that were found in all analysed genomes.

**A. 1. GGDEF proteins found in the *A. brasilense* Sp245 genome**

| **LOCUS TAG** | **AA** | **TM DOMAIN** | **SENSING DOMAINS** | **GGDEF DOMAIN** | | |
| --- | --- | --- | --- | --- | --- | --- |
|  |  |  |  | **ACTIVE SITE 2** | **INHIBITORY SITE** | **ACTIVE SITE 1** |
| **WP_014197451** | **540** |  |  | **DxDxFKxxNDxxGHxxGD** | **RxxD** | **GGDEF** |
| **WP_014240775** | **326** |  | **PAS 2 PAS** | **DxDxFKxxNDxxGHxxGD** | **RxxD** | **GGDEF** |
| **WP_014238973** | **560** |  | **PAS** | **DxDxFKxxNDxxGHxxGD** | **RxxD** | **GGEEF** |
| **WP_014239318** | **459** |  | **PAS /GAF** | **DxDxFKxxNDxxGHxxGD** | **RxxD** | **GGDEF** |
| **WP_014241636** | **459** |  | **Protoglobine** | **DxDxFKxxNDxxGHxxGD** | **RxxD** | **GGEEF** |
| **WP_014240083** | **294** |  | **REC** | **DxDxFKxxNDxxGHxxGD** | **RxxD** | **GGEEF** |
| **WP_014239247** | **345** |  | **CZB** | **DxDxFKxxNDxxGHxxGD** | **RxxD** | **GGEEF** |
| **WP_014240625** | **306** |  |  | **DxDxFKxxNDxxGHxxGD** | **KxxD** | **GGEEF** |
| **WP_014239228** | **557** |  | **REC** | **DxDxFKxxNDxxGHxxGD** | **RxxD** | **GGEEF** |
| **WP_014198789** | **517** |  | **2 REC/ HPT** | **DxDxFKxxNDxxGHxxGD** | **RxxD** | **GGEEF** |
| **WP_014239104** | **517** |  | **REC** | **DxDxFKxxNDxxGHxxGD** | **RxxD** | **GGEEF** |
| **WP_014239030** | **465** | **5** |  | **DxDxFKxxNDxxGHxxGD** | **RxxD** | **GGEEF** |
| **WP_014197448** | **298** |  | **2 REC** | **DxDxFKxxNDxxGHxxGD** | **RxxD** | **GGEEF** |
| **WP_014197311** | **338** |  | **REC** | **DxDxFKxxNDxxGHxxGD** | **RxxD** | **GGEEF** |
| **WP_014200048** | **561** |  | **GAF** | **DxDxFKxxNDxxGHxxGD** | **RxxD** | **GGDEF** |
| **WP_014197673** | **578** | **3** | **HAMP** | **DxDxFKxxNDxxGHxxGD** | **RxxD** | **GGDEF** |
| **WP_052584448** | **369** |  | **2 PAS/PAC** | **DxDxFKxxNDxxGHxxGD** | **RxxD** | **GGDEF** |
| **WP_014197071** | **545** | **2** | **PAS/PAC** | **DxDxFKxxNDxxGHxxGD** | **RxxD** | **GGDEF** |
| **WP_052584421** | **415** | **2** | **HAMP** | **DxDxFKxxNDxxGHxxGD** | **RxxD** | **GGDEF** |
| **WP_082188241** | **549** | **2** | **dCACHE_1** | **DxDxFKxxNDxxGHxxGD** | **RxxD** | **GGDEF** |

**A. 2. EAL proteins found in the *A. brasilense* Sp245 genome**

| **LOCUS TAG** | **AA** | **TM DOMAIN** | **SENSING DOMAINS** | **EAL DOMAIN** | | | | | | | |
| --- | --- | --- | --- | --- | --- | --- | --- | --- | --- | --- | --- |
|  |  |  |  | **GMP-BS1** | **MBS1** | **MBS2** | **MBS3** | **LOOP 6** | **H_2_O BS** | **MBS4** | **GMP-BS2** |
| **WP_014241505** | **411** | **2** |  | **Q** | **EVFxR** | **N** | **E** | **DRVSDLDIDV** | **K** | **E** | **Q** |
| **WP_014239906** | **560** |  | **PAS** | **Q** | **EALxR** | **N** | **E** | **DDVGAGSTSF** | **K** | **E** | **Q** |
| **WP_014240028** | **262** |  |  | **Q** | **EALxR** | **N** | **E** | **DDFGSGYSGL** | **K** | **E** | **Q** |
| **WP_014242202** | **556** |  | **PAS** | **Q** | **EALxR** | **N** | **E** | **DDVGSGTTSF** | **K** | **E** | **Q** |
| **WP_014199814** | **278** |  |  | **Q** | **EALxR** | **N** | **E** | **DDFGTGYSSL** | **K** | **E** | **Q** |

| **LOCUS TAG** | **AA** | **TM DOMAIN** | **SENSING DOMAINS** | **GGDEF DOMAIN** | | | **EAL DOMAIN** | | | | | | | |
| --- | --- | --- | --- | --- | --- | --- | --- | --- | --- | --- | --- | --- | --- | --- |
|  |  |  |  | **ACTIVE SITE 2** | **INHIBITORY SITE** | **ACTIVE SITE 1** | **GMP-BS1** | **MBS1** | **MBS2** | **MBS3** | **LOOP 6** | **H_2_O BS** | **MBS4** | **GMP-BS2** |
| [**WP_014239522**](https://www.ncbi.nlm.nih.gov/protein/504005528?report=genbank&log$=prottop&blast_rank=1&RID=RGE8YSH121S) | **1080** | **1** | **2 CACHE/ 2 PAS** | **DxDxFKxxNDxxGHxxGD** | **RxxD** | **GGDEF** | **Q** | **EGLxR** | **N** | **E** | **DDFGTGYSSL** | **K** | **E** | **Q** |
| [**WP_014199833**](https://www.ncbi.nlm.nih.gov/protein/503965839?report=genbank&log$=prottop&blast_rank=2&RID=RGE8YSH121S) | **687** |  | **PAS** | **DxNxFKxxNDxxGHxxGD** | **RxxD** | **GGDEF** | **Q** | **EALxR** | **N** | **E** | **DDFGTGYSSL** | **K** | **E** | **Q** |
| [**WP_014240087**](https://www.ncbi.nlm.nih.gov/protein/504006093?report=genbank&log$=prottop&blast_rank=3&RID=RGE8YSH121S) | **946** | **2** | **CHASE /2 PAS** | **DxSxFKxxNDxxGHxxGD** | **RxxD** | **GGDEF** | **Q** | **EALxR** | **N** | **E** | **DDFGTGYSSL** | **K** | **E** | **Q** |
| [**WP_014199675**](https://www.ncbi.nlm.nih.gov/protein/503965681?report=genbank&log$=prottop&blast_rank=4&RID=RGE8YSH121S) | **794** | **7** | **MHYT /PAS** | **DxDxFVxxRTxxGQxxAN** | **PxxD** | **SGDEF** | **Q** | **EALxR** | **N** | **E** | **DDFGTGYSSL** | **K** | **E** | **Q** |
| [**WP_014241589**](https://www.ncbi.nlm.nih.gov/protein/504007595?report=genbank&log$=prottop&blast_rank=5&RID=RGE8YSH121S) | **794** | **1** | **HAMP/PAS** | **DxDxFKxxTDxxGHxxGD** | **RxxD** | **GTDDF** | **Q** | **EALxR** | **N** | **E** | **DDFGTGHSSL** | **K** | **E** | **Q** |
| [**WP_014241415**](https://www.ncbi.nlm.nih.gov/protein/504007421?report=genbank&log$=prottop&blast_rank=6&RID=RGE8YSH121S) | **580** |  | **2 PAS** | **DxDxFSxxNDxxGHxxGD** | **SxxV** | **GGDEF** | **Q** | **EALxR** | **N** | **E** | **DDFGTGYSSL** | **K** | **E** | **Q** |
| [**WP_014198291**](https://www.ncbi.nlm.nih.gov/protein/503964297?report=genbank&log$=prottop&blast_rank=7&RID=RGE8YSH121S) | **838** |  | **3PAS** | **DxNxFKxxNDxxGSxxGD** | **RxxD** | **SADEF** | **Q** | **EALxR** | **N** | **E** | **DDFGTGYSSL** | **K** | **E** | **Q** |
| [**WP_014198689**](https://www.ncbi.nlm.nih.gov/protein/503964695?report=genbank&log$=prottop&blast_rank=8&RID=RGE8YSH121S) | **692** |  | **2PAS** | **DxDxFSxxNExxGFxxGD** | **PxxT** | **AADEF** | **Q** | **EALxR** | **N** | **E** | **DDFGSGYSSL** | **K** | **E** | **Q** |
| [**WP_014239353**](https://www.ncbi.nlm.nih.gov/protein/504005359?report=genbank&log$=prottop&blast_rank=9&RID=RGE8YSH121S) | **582** |  | **PAS** | **DxDxFVxxRTxxGQxxAN** | **GxxD** | **SDHAF** | **Q** | **EALxR** | **N** | **E** | **DDFGTGYSSL** | **K** | **E** | **Q** |
| [**WP_014239107**](https://www.ncbi.nlm.nih.gov/protein/504005113?report=genbank&log$=prottop&blast_rank=10&RID=RGE8YSH121S) | **624** |  |  | **DxDxFKxxNDxxGFxxGD** | **GxxG** | **GGDDF** | **Q** | **EALxR** | **D** | **E** | **DDFGVGFAGL** | **K** | **E** | **Q** |

**A. 3 Hybrid proteins found in the *A. brasilense* Sp245 genome**

**B. 1. The GGDEF proteins found in the *A. brasilense* Sp7 genome**

| **LOCUS TAG** | **AA** | **TM DOMAIN** | **SENSING DOMAINS** | **GGDEF DOMAIN** | | |
| --- | --- | --- | --- | --- | --- | --- |
|  |  |  |  | **ACTIVE SITE 2** | **INHIBITORY SITE** | **ACTIVE SITE 1** |
| **WP_051140034** | **326** |  | **1 PAS** | **DxDxFKxxNDxxGHxxGD** | **RxxD** | **GGEEF** |
| **WP_035670654** | **375** | **5** |  | **DxDxFKxxNDxxGHxxGD** | **RxxD** | **GGEEF** |
| **WP_035670844** | **517** |  | **REC** | **DxDxFKxxNDxxGHxxGD** | **RxxD** | **GGEEF** |
| **WP_035671042** | **306** |  | **REC** | **DxDxFKxxNDxxGHxxGD** | **RxxD** | **GGEEF** |
| **WP_035671094** | **304** |  |  | **DxDxFKxxNDxxGHxxGD** | **RxxD** | **GGEEF** |
| **WP_035671267** | **564** |  | **1PAS/1GAF** | **DxDxFKxxNDxxGHxxGD** | **RxxD** | **GGDEF** |
| **WP_035672942** | **294** |  | **REC** | **DxDxFKxxNDxxGHxxGD** | **RxxD** | **GGEEF** |
| **WP_035674304** | **345** |  |  | **DxDxFKxxNDxxGHxxGD** | **KxxD** | **GGEEF** |
| **WP_035674663** | **540** |  | **3 PAS** | **DxDxFKxxNDxxGHxxGD** | **RxxD** | **GGEEF** |
| **WP_035675850** | **456** |  | **PROTOGLOBIN** | **DxDxFKxxNDxxGHxxGD** | **RxxD** | **GGEEF** |
| **WP_051140383** | **369** | **2** | **1 PAS** | **DxDxFKxxNDxxGHxxGD** | **RxxD** | **GGEEF** |
| **WP_035671246** | **298** |  | **REC** | **DxDxFKxxNDxxGHxxGD** | **RxxD** | **GGEEF** |
| **WP_035676633** | **465** |  | **2 REC** | **DxDxFKxxNDxxGHxxGD** | **RxxD** | **GGEEF** |
| **WP_059399331** | **561** | **1** | **HAMP** | **DxDxFKxxNDxxGHxxGD** | **RxxD** | **GGDEF** |
| **WP_059399097** | **545** | **2** | **HAMP** | **DxDxFKxxNDxxGHxxGD** | **RxxD** | **GGDEF** |
| **WP_035678542** | **443** | **1** |  | **DxDxFKxxNDxxGHxxGD** | **RxxD** | **GGDEF** |
| **WP_035682812** | **338** |  | **GAF** | **DxDxFKxxNDxxGHxxGD** | **RxxD** | **GGDEF** |
| **WP_059399449** | **579** |  | **2 PAS** | **DxDxFKxxNDxxGHxxGD** | **RxxD** | **GGDEF** |
| **WP_079285367** | **231** |  |  | **DxDxFKxxNDxxGHxxGD** | **RxxD** | **GGDEF** |
| **WP_051140397** | **387** | **7** |  | **DxDxFKxxNDxxGHxxGD** | **RxxD** | **GGEEF** |

**B. 2. The EAL proteins found in the *A. brasilense* Sp7 genome**

| **LOCUS TAG** | **AA** | **TM DOMAIN** | **SENSING DOMAINS** | **EAL DOMAIN** | | | | | | | |
| --- | --- | --- | --- | --- | --- | --- | --- | --- | --- | --- | --- |
|  |  |  |  | **GMP-BS1** | **MBS1** | **MBS2** | **MBS3** | **LOOP 6** | **H_2_O BS** | **MBS4** | **GMP-BS2** |
| **WP_051140161** | **571** |  | **PAS** | **Q** | **EALxR** | **N** | **E** | **DDVGAGSTSFQS** | **K** | **E** | **Q** |
| **CAJ18244/*chsA*** | **586** |  | **PAS** | **Q** | **EALxR** | **N** | **E** | **DDVGAGSTSFQS** | **K** | **E** | **Q** |
| **WP_059398606** | **403** | **2** |  | **Q** | **EVFxR** | **N** | **E** | **DRVSDLDIDVDAL** | **K** | **E** | **Q** |
| **WP_035682417** | **278** |  |  | **Q** | **EALxR** | **N** | **E** | **DDFGTGYSSLSLL** | **K** | **E** | **Q** |
| **WP_03562792** | **260** |  |  | **Q** | **EALxR** | **N** | **E** | **DDFGSGYSGLNLL** | **K** | **E** | **Q** |

**B. 3. The Hybrid proteins found in the *A. brasilense* Sp7 genome**

| **LOCUS TAG** | **AA** | **TM DOMAIN** | **SENSING DOMAINS** | **GGDEF DOMAIN** | | | **EAL DOMAIN** | | | | | | | |
| --- | --- | --- | --- | --- | --- | --- | --- | --- | --- | --- | --- | --- | --- | --- |
|  |  |  |  | **ACTIVE SITE 2** | **INHIBITORY SITE** | **ACTIVE SITE 1** | **GMP-BS1** | **MBS1** | **MBS2** | **MBS3** | **LOOP 6** | **H_2_O BS** | **MBS4** | **GMP-BS2** |
| **WP_059398931** | **582** |  | **PAS** | **DxDxFVxxRTxxGQxxAN** | **GxxD** | **SDHAF** | **Q** | **EALxR** | **N** | **E** | **DDFGTGYSSL** | **K** | **E** | **Q** |
| **WP_079285130** | **794** | **2** | **HAMP/PAS** | **DxSxFKxxTDxxGHxxGD** | **RxxD** | **GTDDF** | **Q** | **EALxR** | **N** | **E** | **DDFGTGHSSL** | **K** | **E** | **Q** |
| **WP_051140628** | **692** |  | **PAS** | **DxDxFSxxNExxGFxxGD** | **PxxT** | **AADEF** | **Q** | **EALxR** | **N** | **E** | **DDFGSGYSSL** | **K** | **E** | **Q** |
| **WP_059399067** | **631** |  |  | **DxDxFKxxNDxxGFxxGD** | **RxxD** | **GGDDF** | **Q** | **EALxR** | **N** | **E** | **DDFGVGFAGL** | **K** | **E** | **Q** |
| **WP_035678503** | **838** |  | **3 PAS** | **DxDxFKxxNDxxGSxxGD** | **RxxD** | **SADEF** | **Q** | **EALxR** | **N** | **E** | **DDFGTGYSSL** | **K** | **E** | **Q** |
| **WP_059399655** | **794** | **6** | **MHYT/PAS** | **DxDxFKxxNDxxGHxxGD** | **PxxD** | **SGDEF** | **Q** | **EALxR** | **N** | **E** | **DDFGTGYSSL** | **K** | **E** | **Q** |
| **WP_051140186** | **928** | **2** | **CHASE/ PAS** | **DxDxFKxxNDxxGHxxGD** | **RxxD** | **GGDEF** | **Q** | **EALxR** | **N** | **E** | **DDFGTGYSSL** | **K** | **E** | **Q** |
| **WP_051140104** | **1080** | **1** | **2 CACHE/ 2 PAS** | **DxDxFKxxNDxxGHxxGD** | **RxxD** | **GGDEF** | **Q** | **EGLxR** | **N** | **E** | **DDFGTGYSSL** | **K** | **E** | **Q** |
| **WP_059399677** | **687** |  | **2 PAS** | **DxNxFKxxNDxxGHxxGD** | **RxxD** | **GGDEF** | **Q** | **EALxR** | **N** | **E** | **DDFGTGYSSL** | **K** | **E** | **Q** |

**C. 1 GGDEF proteins found in the *A. brasilense* Az39 genome**

| **LOCUS TAG** | **AA** | **TM DOMAIN** | **SENSING DOMAINS** | **GGDEF DOMAIN** | | |
| --- | --- | --- | --- | --- | --- | --- |
|  |  |  |  | **ACTIVE SITE 2** | **INHIBITORY SITE** | **ACTIVE SITE 1** |
| **WP_038528453** | **294** |  | **REC** | **DxDxFKxxNDxxGHxxGD** | **RxxD** | **GGEEF** |
| **WP_038530483** | **532** |  | **GAF/PAS** | **DxDxFKxxNDxxGHxxGD** | **RxxD** | **GGDEF** |
| **WP_038530041** | **375** | **5** |  | **DxDxFKxxNDxxGHxxGD** | **RxxD** | **GGEEF** |
| **WP_051658431** | **552** |  | **2 PAS** | **DxDxFKxxNDxxGHxxGD** | **RxxD** | **GGDEF** |
| **WP_040134391** | **465** |  | **2 REC** | **DxDxFKxxNDxxGHxxGD** | **RxxD** | **GGEEF** |
| **WP_040138238** | **338** |  | **GAF** | **DxDxFKxxNDxxGHxxGD** | **RxxD** | **GGDEF** |
| **WP_038527442** | **345** |  |  | **DxDxFKxxNDxxGHxxGD** | **KxxD** | **GGEEF** |
| **WP_051658417** | **545** | **2** | **HAMP** | **DxDxFKxxNDxxGHxxGD** | **RxxD** | **GGDEF** |
| **WP_040133993** | **298** |  | **REC** | **DxDxFKxxNDxxGHxxGD** | **RxxD** | **GGEEF** |
| **WP_051658298** | **561** | **2** | **HAMP** | **DxDxFKxxNDxxGHxxGD** | **RxxD** | **GGDEF** |
| **WP_051658027** | **326** |  | **PAS** | **DxDxFKxxNDxxGHxxGD** | **RxxD** | **GGEEF** |
| **WP_051658204** | **369** | **2** | **PAS** | **DxDxFKxxNDxxGHxxGD** | **RxxD** | **GGDEF** |
| **WP_063922679** | **269** |  |  | **DxDxFKxxNDxxGHxxGD** | **RxxD** | **GGDEF** |
| **WP_081863206** | **544** | **2** | **PERIPLASMIC SENSOR** | **DxDxFKxxNDxxGHxxGD** | **RxxD** | **GGDEF** |
| **WP_051658186** | **387** | **7** |  | **DxDxFKxxNDxxGHxxGD** | **RxxD** | **GGEEF** |
| **WP_038527678** | **540** |  | **2 PAS** | **DxDxFKxxNDxxGHxxGD** | **RxxD** | **GGEEF** |
| **WP_038525874** | **306** |  | **REC** | **DxDxFKxxNDxxGHxxGD** | **RxxD** | **GGEEF** |
| **WP_038525904** | **304** |  |  | **DxDxFKxxNDxxGHxxGD** | **RxxD** | **GGEEF** |
| **WP_038529941** | **517** |  | **REC** | **DxDxFKxxNDxxGHxxGD** | **RxxD** | **GGEEF** |
| **WP_038529622** | **459** |  | **GLOBIN LIKE** | **DxDxFKxxNDxxGHxxGD** | **RxxD** | **GGEEF** |

**C. 2 EAL proteins found in the *A. brasilense* Az39 genome**

| **LOCUS TAG** | **AA** | **TM DOMAIN** | **SENSING DOMAINS** | **EAL DOMAIN** | | | | | | | |
| --- | --- | --- | --- | --- | --- | --- | --- | --- | --- | --- | --- |
|  |  |  |  | **GMP-BS1** | **MBS1** | **MBS2** | **MBS3** | **LOOP 6** | **H_2_O BS** | **MBS4** | **GMP-BS2** |
| **WP_038529539** | **400** | **2** |  | **Q** | **EVFxR** | **N** | **E** | **DRVSDLSIDV** | **K** | **E** | **Q** |
| **WP_051657894** | **571** |  | **PAS** | **Q** | **EALxR** | **N** | **E** | **DDVGAGSTSF** | **K** | **E** | **Q** |
| **WP_051658176** | **556** |  | **PAS** | **Q** | **EALxR** | **N** | **E** | **DDVGSGTTSF** | **K** | **E** | **Q** |
| **WP_040138014** | **273** |  |  | **Q** | **EALxR** | **N** | **E** | **DDFGTGYSSL** | **K** | **E** | **Q** |
| **WP_038528539** | **265** |  |  | **Q** | **EALxR** | **N** | **E** | **DDFGSGYSGL** | **K** | **E** | **Q** |

**C. 3. Hybrid proteins found in the *A. brasilense* Az39 genome**

| **LOCUS TAG** | **AA** | **TM DOMAIN** | **SENSING DOMAINS** | **GGDEF DOMAIN** | | | **EAL DOMAIN** | | | | | | | |
| --- | --- | --- | --- | --- | --- | --- | --- | --- | --- | --- | --- | --- | --- | --- |
|  |  |  |  | **ACTIVE SITE 2** | **INHIBITORY SITE** | **ACTIVE SITE 1** | **GMP-BS1** | **MBS1** | **MBS2** | **MBS3** | **LOOP 6** | **H_2_O BS** | **MBS4** | **GMP-BS2** |
| **WP_038531496** | **613** |  |  | **DxNxFKxxNDxxGFxxGD** | **GxxG** | **GGDDF** | **Q** | **EALxR** | **N** | **E** | **DDFGVGFAGL** | **K** | **E** | **Q** |
| **WP_040135003** | **838** |  | **3 PAS** | **DxDxFKxxNDxxGSxxGD** | **RxxD** | **SADEF** | **Q** | **EALxR** | **N** | **E** | **DDFGTGYSSL** | **K** | **E** | **Q** |
| **WP_038526104** | **582** |  | **PAS** | **DxDxFVxxRTxxGQxxAN** | **GxxD** | **SDHAF** | **Q** | **EALxR** | **N** | **E** | **DDFGTGYSSL** | **K** | **E** | **Q** |
| **WP_040134184** | **692** |  | **2 PAS** | **DxDxFSxxNExxGFxxGD** | **PxxT** | **AADEF** | **Q** | **EALxR** | **N** | **E** | **DDFGSGYSSL** | **K** | **E** | **Q** |
| **WP_081862986** | **794** | **1** | **PAS/HAMP** | **DxSxFKxxTDxxGHxxGD** | **RxxD** | **GTDDF** | **Q** | **EALxR** | **N** | **E** | **DDFGTGHSSL** | **K** | **E** | **Q** |
| **WP_040138308** | **794** | **6** | **PAS/MHYT** | **DxDxFKxxNDxxGHxxGD** | **PxxD** | **SGDEF** | **Q** | **EALxR** | **N** | **E** | **DDFGTGYSSL** | **K** | **E** | **Q** |
| **WP_063922630** | **915** | **2** | **PAS/CHASE** | **DxDxFKxxNDxxGHxxGD** | **RxxD** | **GGDEF** | **Q** | **EALxR** | **N** | **E** | **DDFGTGYSSL** | **K** | **E** | **Q** |
| **WP_040138037** | **687** |  | **2 PAS** | **DxNxFKxxNDxxGHxxGD** | **RxxD** | **GGDEF** | **Q** | **EALxR** | **N** | **E** | **DDFGTGYSSL** | **K** | **E** | **Q** |
| **WP_038531265** | **1076** | **1** | **2 PAS/2CACHE** | **DxDxFKxxNDxxGHxxGD** | **RxxD** | **GGDEF** | **Q** | **EGLxR** | **N** | **E** | **DDFGTGYSSL** | **K** | **E** | **Q** |
| **WP_038529454** | **580** |  | **PAS** | **DxDxFKxxNDxxGHxxGD** | **GxxV** | **GGDEF** | **Q** | **EALxR** | **D** | **E** | **DDFGTGYSSL** | **K** | **E** | **Q** |

**D. 1. The GGDEF domain proteins found in *A. lipoferum* 4B genome**

| **LOCUS TAG** | **AA** | **TM DOMAIN** | **SENSING DOMAINS** | **GGDEF DOMAIN** | | |
| --- | --- | --- | --- | --- | --- | --- |
|  |  |  |  | **ACTIVE SITE 2** | **INHIBITORY SITE** | **ACTIVE SITE 1** |
| **WP_014249136** | **391** | **5** |  | **DxDxFKxxNDxxGHxxGD** | **RxxD** | **GGEEF** |
| **WP_014246507** | **308** |  | **REC** | **DxDxFKxxNDxxGHxxGD** | **PxxG** | **GGEEF** |
| **WP_014247155** | **342** |  |  | **DxDxFKxxNDxxGHxxGD** | **KxxD** | **GGEEF** |
| **WP_014248701** | **305** |  | **REC** | **DxDxFKxxNDxxGHxxGD** | **RxxD** | **GGEEF** |
| **WP_014188334** | **396** | **7** |  | **DxDxFKxxNDxxGHxxGD** | **RxxD** | **GGEEF** |
| **WP_014249410** | **575** |  | **REC/PAS** | **DxDxFKxxNDxxGHxxGD** | **RxxD** | **GGEEF** |
| **WP_014249756** | **477** | **1** | **PBPb** | **DxDxFKxxNDxxGHxxGD** | **RxxD** | **GGEEF** |
| **WP_014250202** | **538** |  | **3 PAS** | **DxDxFKxxNDxxGHxxGD** | **KxxD** | **GGEEF** |
| **WP_014246736** | **353** |  | **REC** | **DxDxFKxxNDxxGDxxGD** | **RxxD** | **GGAEF** |
| **WP_014189705** | **562** |  | **3 PAS** | **DxDxFKxxNDxxGHxxGD** | **RxxD** | **GGEEF** |
| **WP_014248328** | **304** |  | **REC** | **DxDxFKxxNDxxGHxxGD** | **RxxD** | **GGEEF** |
| **WP_014247337** | **468** |  | **REC/PAS** | **DxDxFKxxNDxxGHxxGD** | **RxxD** | **GGEEF** |
| **WP_014248103** | **238** |  |  | **DxDxFKxxNDxxGHxxGD** | **RxxD** | **GGDEF** |
| **WP_014248886** | **302** |  |  | **DxDxFKxxNDxxGHxxGD** | **RxxD** | **GGEEF** |
| **WP_065814210** | **584** |  | **PAS/GAF** | **DxDxFKxxNDxxGHxxGD** | **RxxD** | **GGDEF** |
| **WP_014249593** | **258** |  |  | **DxDxFKxxNDxxGHxxGD** | **RxxD** | **GGEEF** |
| **WP_014248101** | **464** |  | **2 REC** | **DxDxFKxxNDxxGHxxGD** | **RxxD** | **GGEEF** |
| **WP_014188845** | **372** | **6** |  | **DxDxFKxxNDxxGHxxGD** | **RxxD** | **GGEEF** |
| **WP_014189160** | **640** | **2** | **PDB/PAS** | **DxDxFKxxNDxxGHxxGD** | **RxxD** | **GGEEF** |

**D. 2. The EAL domain proteins found in *A. lipoferum* 4B genome**

| **LOCUS TAG** | **AA** | **TM DOMAIN** | **SENSING DOMAINS** | **EAL DOMAIN** | | | | | | | |
| --- | --- | --- | --- | --- | --- | --- | --- | --- | --- | --- | --- |
|  |  |  |  | **GMP-BS1** | **MBS1** | **MBS2** | **MBS3** | **LOOP 6** | **H_2_O BS** | **MBS4** | **GMP-BS2** |
| **WP_014248761** | **435** | **2** |  | **Q** | **EALxR** | **N** | **E** | **DQVTDLAIDL** | **K** | **E** | **Q** |
| **WP_044549771** | **253** |  |  | **Q** | **EALxR** | **N** | **E** | **DDFGSGYSGL** | **K** | **E** | **Q** |
| **WP_044550563** | **562** |  | **PAS** | **Q** | **EALxR** | **N** | **E** | **DDVGAGSTSF** | **K** | **E** | **Q** |
| **WP_014249491** | **541** |  |  | **Q** | **EALxR** | **N** | **E** | **DDFGAGAASF** | **K** | **E** | **Q** |
| **WP_044549890** | **294** |  |  | **Q** | **EALxR** | **N** | **E** | **DDFGTGWSSL** | **K** | **E** | **Q** |

| **LOCUS TAG** | **AA** | **TM DOMAIN** | **SENSING DOMAINS** | **GGDEF DOMAIN** | | | **EAL DOMAIN** | | | | | | | |
| --- | --- | --- | --- | --- | --- | --- | --- | --- | --- | --- | --- | --- | --- | --- |
|  |  |  |  | **ACTIVE SITE 2** | **INHIBITORY SITE** | **ACTIVE SITE 1** | **GMP-BS1** | **MBS1** | **MBS2** | **MBS3** | **LOOP 6** | **H_2_O BS** | **MBS4** | **GMP-BS2** |
| **WP_014248850** | **622** |  |  | **DFDNFKPFNDNFGFRQGD** | **RLKA** | **GGDDF** | **Q** | **EALxR** | **N** | **E** | **DDFGVGHSGL** | **K** | **E** | **Q** |
| **WP_014188667** | **639** |  | **GAF** | **DxDxFSxxNExxGFxxGD** | **RxxA** | **SPARF** | **Q** | **EALxR** | **N** | **E** | **DDFGTGQSAL** | **K** | **E** | **Q** |
| **WP_014247398** | **741** |  | **2 PAS** | **DxDxFSxxNExxGFxGD** | **RxxE** | **AADEF** | **Q** | **EALxR** | **N** | **E** | **DDFGSGYSSL** | **K** | **E** | **Q** |
| **WP_014188795** | **759** |  | **REC** | **DxDxFQxxNDxxGHxxGD** | **RxxS** | **ASDMF** | **Q** | **EALxR** | **N** | **E** | **DDFGTGYSSL** | **K** | **E** | **Q** |
| **WP_014248011** | **712** |  | **3 PAS** | **DxDxFKxxNDxxGTxxGD** | **RxxD** | **SADEF** | **Q** | **EALxR** | **N** | **E** | **DDFGTGYSSL** | **K** | **E** | **Q** |
| **WP_014248989** | **581** |  | **PAS** | **DxDxFAxxRSxxGQxxAN** | **RxxA** | **SDHAF** | **Q** | **EALxR** | **N** | **E** | **DDFGTGYSSL** | **K** | **E** | **Q** |
| **WP_014249547** | **1004** | **2** | **2 PAS** | **DxDxFKxxNDxxGHxxGD** | **RxxD** | **SGDEF** | **Q** | **EALxR** | **N** | **E** | **DDFGTGFSSL** | **K** | **E** | **Q** |
| **WP_014246612** | **1006** | **2** | **PAS** | **DxDxFKxxNDxxGHxxGD** | **RxxG** | **GGDEF** | **Q** | **EALxR** | **N** | **E** | **DDFGTGYSSL** | **K** | **E** | **Q** |
| **WP_014246659** | **753** |  | **PAS/GAF** | **DxDxFKxxNDxxGHxxGD** | **RxxF** | **GGDEF** | **Q** | **EALxR** | **N** | **E** | **DDFGTGYSSL** | **K** | **E** | **Q** |
| **WP_014246690** | **907** | **1** | **PAS** | **GxDxFKxxNDxxGHxxGD** | **RxxD** | **GGDEF** | **Q** | **EALxR** | **D** | **E** | **DDFGTGYSNL** | **K** | **E** | **Q** |
| **WP_014248963** | **789** | **1** | **PAS/HAMP** | **DxSxFKxxTDxxGHxxGD** | **RxxD** | **GTDDF** | **Q** | **EALxR** | **N** | **E** | **DDFGTGHSSL** | **K** | **E** | **Q** |
| **WP_014189550** | **696** | **2** | **HAMP** | **DxDxFKxxNDxxGHxxGD** | **RxxD** | **GGDEF** | **Q** | **EALxR** | **N** | **E** | **DDFGTGYSSL** | **K** | **E** | **Q** |
| **WP_014248694** | **711** | **1** | **HAMP** | **DxDxFKxxNDxxGHxxGD** | **RxxD** | **GGDEF** | **Q** | **EALxR** | **N** | **E** | **DDFGTGYSSL** | **K** | **E** | **Q** |
| **WP_014250090** | **934** | **2** | **PAS/CHASE** | **DxDxFKxxNDxxGHxxGD** | **RxxD** | **GGDEF** | **Q** | **EALxR** | **N** | **E** | **DDFGTGYSSL** | **K** | **E** | **Q** |
| **WP_014246975** | **1085** | **1** | **2 PAS/2CACHE** | **DxDxFKxxNDxxGHxxGD** | **RxxD** | **GGDEF** | **Q** | **EALxR** | **N** | **E** | **DDFGTGYSSL** | **K** | **E** | **Q** |
| **WP_14249492** | **685** |  | **2 PAS** | **DxNxFKxxNDxxGHxxGD** | **RxxD** | **GGDEF** | **Q** | **EALxR** | **N** | **E** | **DDFGTGYSSL** | **K** | **E** | **Q** |

**D. 3. The hybrid proteins found in *A. lipoferum* 4B genome**

| **LOCUS TAG** | **AA** | **TM DOMAIN** | **SENSING DOMAINS** | **GGDEF DOMAIN** | | |
| --- | --- | --- | --- | --- | --- | --- |
|  |  |  |  | **ACTIVE SITE 2** | **INHIBITORY SITE** | **ACTIVE SITE 1** |
| **WP_042445615** | **565** | **2** |  | **DxDxFKxxNDxxGHxxGD** | **RxxD** | **GGDEF** |
| **WP_012974194** | **338** |  | **GAF** | **DxDxFKxxNDxxGHxxGD** | **RxxD** | **GGDEF** |
| **WP_012974483** | **238** |  |  | **DxDxFKxxNDxxGHxxGD** | **RxxD** | **GGDEF** |
| **WP_012972667** | **461** | **5** |  | **DxDxFKxxNDxxGHxxGD** | **RxxD** | **GGEEF** |
| **WP_012973137** | **595** |  | **PAS/GAF** | **DxDxFKxxNDxxGHxxGD** | **RxxD** | **GGEEF** |
| **WP_012972950** | **302** |  |  | **DxDxFKxxNDxxGHxxGD** | **RxxD** | **GGDEF** |
| **WP_012974295** | **388** | **6** |  | **DxDxFKxxNDxxGHxxGD** | **RxxD** | **GGEEF** |
| **WP_012973551** | **342** |  |  | **DxDxFKxxNDxxGHxxGD** | **KxxD** | **GGEEF** |
| **WP_012977574** | **262** |  |  | **DxDxFKxxNDxxGHxxGD** | **RxxD** | **GGEEF** |
| **WP_012974989** | **310** |  | **REC** | **DxDxFKxxNDxxGHxxGD** | **RxxD** | **GGEEF** |
| **WP_012972725** | **385** | **7** |  | **DxDxFKxxNDxxGHxxGD** | **RxxD** | **GGEEF** |
| **WP_012975963** | **402** | **7** |  | **DxDxFKxxNDxxGHxxGD** | **RxxD** | **GGEEF** |
| **WP_012973038** | **350** |  | **REC** | **DxDxFExxNDxxGNxxGD** | **RxxD** | **GGDEF** |
| **WP_042442983** | **304** |  | **REC** | **DxDxFKxxNDxxGHxxGD** | **RxxD** | **GGEEF** |
| **WP_012977364** | **545** | **2** | **REC/ PAS** | **DxDxFKxxNDxxGHxxGD** | **RxxD** | **GGEEF** |
| **WP_012976244** | **379** | **6** |  | **DxDxFKxxNDxxGHxxGD** | **RxxD** | **GGEEF** |
| **WP_012976938** | **627** | **2** | **PROTOGLOBIN** | **DxDxFKxxNDxxGHxxGD** | **RxxD** | **GGEEF** |
| **WP_012974481** | **231** |  | **2 REC** | **DxDxFKxxNDxxGHxxGD** | **RxxD** | **GGDEF** |
| **WP_012973965** | **516** |  | **2 PAS** | **DxDxFKxxNDxxGHxxGD** | **RxxD** | **GGEEF** |

**E. 1. The GGDEF proteins found in *Azospirillum* B510 genome.**

**E. 2. The EAL proteins found in *Azospirillum* B510 genome**

| **LOCUS TAG** | **AA** | **TM DOMAIN** | **SENSING DOMAINS** | **EAL DOMAIN** | | | | | | | |
| --- | --- | --- | --- | --- | --- | --- | --- | --- | --- | --- | --- |
|  |  |  |  | **GMP-BS1** | **MBS1** | **MBS2** | **MBS3** | **LOOP 6** | **H_2_O BS** | **MBS4** | **GMP-BS2** |
| **WP_012975037** | **462** | **2** |  | **Q** | **EVFxR** | **N** | **E** | **DQVTDLAIDI** | **K** | **E** | **Q** |
| **WP_012974608** | **315** |  |  | **Q** | **EALxR** | **N** | **E** | **DDFGTGWSSL** | **K** | **E** | **Q** |
| **WP_012974146** | **263** |  |  | **Q** | **EALxR** | **N** | **E** | **DDFGSGYSGL** | **K** | **E** | **Q** |
| **WP_012974361** | **585** |  | **PAS** | **Q** | **EALxR** | **N** | **E** | **DDVGAGSTSF** | **K** | **E** | **Q** |
| **WP_012977214** | **619** |  | **PAS** | **Q** | **EALxR** | **N** | **E** | **DDFGAGAASF** | **K** | **E** | **Q** |

| **LOCUS TAG** | **AA** | **TM DOMAIN** | **SENSING DOMAINS** | **GGDEF DOMAIN** | | | **EAL DOMAIN** | | | | | | | |
| --- | --- | --- | --- | --- | --- | --- | --- | --- | --- | --- | --- | --- | --- | --- |
|  |  |  |  | **ACTIVE SITE 2** | **INHIBITORY SITE** | **ACTIVE SITE 1** | **GMP-BS1** | **MBS1** | **MBS2** | **MBS3** | **LOOP 6** | **H_2_O BS** | **MBS4** | **GMP-BS2** |
| **WP_012972789** | **581** |  | **PAS** | **DxDxFAxxRSxxGQxxAN** | **TxxD** | **SDHAF** | **Q** | **EALxR** | **N** | **E** | **DDFGTGYSSL** | **K** | **E** | **Q** |
| **WP_012972811** | **791** | **1** | **HAMP/PAS** | **DxSxFKxxTDxxGHxxGD** | **RxxD** | **GIDDF** | **Q** | **EALxR** | **N** | **E** | **DDFGTGHSSL** | **K** | **E** | **Q** |
| **WP_012972922** | **750** |  | **PAS/GAF** | **DxDxFKxxNDxxGHxxGD** | **IxxD** | **GGDEF** | **Q** | **EALxR** | **N** | **E** | **DDFGTGYSSL** | **K** | **E** | **Q** |
| **WP_012972934** | **1021** | **2** | **PAS** | **DxDxFKxxNDxxGHxxGD** | **GxxV** | **GGDEF** | **Q** | **EALxR** | **N** | **E** | **DDFGTGYSSL** | **K** | **E** | **Q** |
| **WP_012972982** | **622** |  | **CBS** | **DxDxFKxxNDxxGFxxGD** | **DxxA** | **GGDDF** | **Q** | **EALxR** | **N** | **E** | **DDFGVGHSGL** | **K** | **E** | **Q** |
| **WP_052293616** | **1106** | **3** | **2 CACHE/2 PAS** | **DxDxFKxxNDxxGHxxGD** | **RxxD** | **GGDEF** | **Q** | **EALxR** | **N** | **E** | **DDFGTGYSSL** | **K** | **E** | **Q** |
| **WP_012973859** | **819** |  | **2 PAS** | **DxDxFSxxNExxGFxxGD** | **PxxT** | **AADEF** | **Q** | **EALxR** | **N** | **E** | **DDFGSGYSSL** | **K** | **E** | **Q** |
| **WP_042442896** | **840** |  | **2 PAS** | **DxDxFKxxNDxxGTxxGD** | **RxxD** | **SADEF** | **Q** | **EALxR** | **N** | **E** | **DDFGTGYSSL** | **K** | **E** | **Q** |
| **WP_012974978** | **746** | **2** |  | **DxDxFKxxNDxxGHxxGD** | **RxxD** | **GGDDF** | **Q** | **EALxR** | **N** | **E** | **DDFGTGYSSL** | **K** | **E** | **Q** |
| **WP_012975124** | **879** | **2** | **SBC/PAS** | **GxDxFKxxNDxxGHxxGD** | **RxxD** | **GGDEF** | **Q** | **EALxR** | **N** | **E** | **DDFGTGYSNL** | **K** | **E** | **Q** |
| **WP_012975524** | **624** |  | **PAS** | **DxDxFKxxNDxxGHxxGD** | **GxxA** | **GGDEF** | **Q** | **EALxR** | **N** | **E** | **DDFGTGYSSL** | **K** | **E** | **Q** |
| **WP_012976217** | **614** |  | **GAF** | **AxQxFAxxTRxxGQxxAD** | **PxxT** | **SPARF** | **Q** | **EALxR** | **N** | **E** | **DDFGTGQSAL** | **K** | **E** | **Q** |
| **WP_042444714** | **764** |  | **REC** | **DxDxFQxxNDxxGHxxGD** | **SxxQ** | **ASDMF** | **Q** | **EALxR** | **N** | **E** | **DDFGTGYSSL** | **K** | **E** | **Q** |
| **WP_012976461** | **875** | **2** | **HAMP/PAS** | **DxDxFKxxNDxxGHxxGD** | **ExxE** | **GGDEF** | **Q** | **EALxR** | **N** | **E** | **DDFGTGYSSL** | **K** | **E** | **Q** |
| **WP_042445660** | **701** |  | **2 PAS** | **DxNxFKxxNDxxGHxxGD** | **RxxD** | **GGDEF** | **Q** | **EALxR** | **N** | **E** | **DDFGTGYSSL** | **K** | **E** | **Q** |
| **WP_012977851** | **688** | **2** | **HAMP** | **DxDxFKxxNDxxGHxxGD** | **RxxD** | **GGDEF** | **Q** | **EALxR** | **N** | **E** | **DDFGTGYSSL** | **K** | **E** | **Q** |
| **WP_012978323** | **819** |  | **CHASE/PAS** | **DxDxFKxxNDxxGHxxGD** | **RxxD** | **GGDEF** | **Q** | **EALxR** | **N** | **E** | **DDFGTGYSSL** | **K** | **E** | **Q** |

**E. 3. The Hybrid protein found in *Azospirillum* B510 genome.**

| **LOCUS TAG** | **AA** | **TM DOMAIN** | **SENSING DOMAINS** | **GGDEF DOMAIN** | | |
| --- | --- | --- | --- | --- | --- | --- |
|  |  |  |  | **ACTIVE SITE 2** | **INHIBITORY SITE** | **ACTIVE SITE 1** |
| **WP_045582086** | **464** |  | **2 REC** | **DxDxFKxxNDxxGHxxGD** | **RxxD** | **GGEEF** |
| **WP_045586291** | **559** | **1** | **CACHE/HAMP** | **DxDxFKxxNDxxGHxxGD** | **RxxD** | **GGDEF** |
| **WP_045583377** | **486** |  | **PAS/REC** | **DxDxFKxxNDxxGHxxGD** | **RxxD** | **GGEEF** |
| **WP_082109442** | **230** |  |  | **DxDxFKxxNDxxGHxxGD** | **RxxD** | **GGEEF** |
| **WP_082108992** | **555** |  | **3 PAS** | **DxDxFKxxNDxxGHxxGD** | **RxxD** | **GGEEF** |
| **WP_045582203** | **553** |  | **3 PAS** | **DxDxFKxxNDxxGHxxGD** | **RxxD** | **GGEEF** |
| **WP_082108818** | **304** |  | **REC** | **DxDxFKxxNDxxGHxxGD** | **RxxD** | **GGEEF** |
| **WP_045581755** | **584** |  | **PAS/GAF** | **DxDxFKxxNDxxGHxxGD** | **RPGD** | **GGDEF** |
| **WP_045584724** | **403** | **7** |  | **DxDxFKxxNDxxGHxxGD** | **RxxD** | **GGEEF** |
| **WP_045581167** | **302** |  |  | **DxDxFKxxNDxxGHxxGD** | **RxxD** | **GGEEF** |
| **WP_082109427** | **482** | **1** | **PBpb** | **DxDxFKxxNDxxGHxxGD** | **RxxD** | **GGEEF** |
| **WP_045582085** | **238** |  |  | **DxDxFKxxNDxxGHxxGD** | **RxxD** | **GGDEF** |
| **WP_082108926** | **341** |  |  | **DxDxFKxxNDxxGHxxGD** | **KxxD** | **GGEEF** |
| **WP_082108776** | **500** | **1** | **GAF** | **DxDxFKxxNDxxGHxxGD** | **RxxD** | **GGEEF** |
| **WP_045582432** | **307** |  | **REC** | **DxDxFKxxNDxxGHxxGD** | **RxxD** | **GGEEF** |
| **WP_045581088** | **368** |  | **REC** | **DxDxFRxxTDxxGLxxGD** | **RxxD** | **GGEEF** |
| **WP_060721713** | **404** | **5** |  | **DxDxFKxxNDxxGHxxGD** | **RxxD** | **GGEEF** |

**F. 1. The GGDEF proteins found in *A. thiophilum* genome**

**F. 2. The EAL proteins found in *A. thiophilum* genome**

| **LOCUS TAG** | **AA** | **TM DOMAIN** | **SENSING DOMAINS** | **EAL DOMAIN** | | | | | | | |
| --- | --- | --- | --- | --- | --- | --- | --- | --- | --- | --- | --- |
|  |  |  |  | **GMP-BS1** | **MBS1** | **MBS2** | **MBS3** | **LOOP 6** | **H_2_O BS** | **MBS4** | **GMP-BS2** |
| **WP_082108838** | **431** | **2** |  | **Q** | **EVFxR** | **N** | **E** | **DDFGTGYSSL** | **K** | **E** | **Q** |
| **WP_082108813** | **271** |  |  | **Q** | **EALxR** | **N** | **E** | **DDVGAGTTSF** | **K** | **E** | **Q** |
| **WP_082108769** | **232** |  |  | **Q** | **EALxR** | **N** | **E** | **DDVGSGSTSF** | **K** | **E** | **Q** |
| **WP_045580288** | **562** |  | **PAS** | **Q** | **EALxR** | **N** | **E** | **DDVGAGSTSF** | **K** | **E** | **Q** |

**F. 3. The Hybrid proteins found in *A. thiophilum* genome.**

| **LOCUS TAG** | **AA** | **TM DOMAIN** | **SENSING DOMAINS** | **GGDEF DOMAIN** | | | **EAL DOMAIN** | | | | | | | |
| --- | --- | --- | --- | --- | --- | --- | --- | --- | --- | --- | --- | --- | --- | --- |
|  |  |  |  | **ACTIVE SITE 2** | **INHIBITORY SITE** | **ACTIVE SITE 1** | **GMP-BS1** | **MBS1** | **MBS2** | **MBS3** | **LOOP 6** | **H_2_O BS** | **MBS4** | **GMP-BS2** |
| **WP_045584990** | **947** | **2** | **PAS/CHASE** | **DxDxFKxxNDxxGHxxGD** | **RxxD** | **GGDEF** | **Q** | **EALxR** | **N** | **E** | **DDFGTGYSSL** | **K** | **E** | **Q** |
| **WP_045583101** | **701** |  | **2 PAS** | **DxNxFKxxNDxxGHxxGD** | **RxxD** | **GGDEF** | **Q** | **EALxR** | **N** | **E** | **DDFGTGYSSL** | **K** | **E** | **Q** |
| **WP_045581881** | **1085** | **2** | **2 PAS/ 2 CACHE2** | **DxDxFKxxNDxxGHxxGD** | **RxxD** | **GGDEF** | **Q** | **EALxR** | **N** | **E** | **DDFGTGYSSL** | **K** | **E** | **Q** |
| **WP_045581669** | **751** |  | **PAS/GAF** | **DxDxFKxxNDxxGHxxGD** | **IxxD** | **GGDEF** | **Q** | **EALxR** | **N** | **E** | **DDFGTGYSSL** | **K** | **E** | **Q** |
| **WP_082108976** | **876** |  | **PAS/PBpb** | **GxDxFKxxNDxxGHxxGD** | **RxxD** | **GGDEF** | **Q** | **EALxR** | **N** | **E** | **DDFGTGYSNL** | **K** | **E** | **Q** |
| **WP_045581219** | **776** | **1** | **PAS/HAMP** | **DxSxFKxxTDxxGHxxGD** | **RxxD** | **GSDDF** | **Q** | **EALxR** | **N** | **E** | **DDFGTGHSSL** | **K** | **E** | **Q** |
| **WP_045583898** | **698** | **2** | **HAMP** | **DxDxFKxxNDxxGHxxGD** | **RxxD** | **GGDEF** | **Q** | **EALxR** | **N** | **E** | **DDFGTGYSSL** | **K** | **E** | **Q** |
| **WP_082108789** | **854** |  | **3 PAS** | **DxDxFKxxNDxxGSxxGD** | **RxxD** | **SADEF** | **Q** | **EALxR** | **N** | **E** | **DDFGTGYSSL** | **K** | **E** | **Q** |
| **WP_082109156** | **925** | **2** | **PAS/HAMP** | **DxDxFKxxNDxxGHxxGD** | **GxxC** | **GGDEF** | **Q** | **EALxR** | **N** | **E** | **DDFGTGYSSL** | **K** | **E** | **Q** |
| **WP_045582559** | **1051** | **2** | **PAS** | **DxDxFKxxNDxxGHxxGD** | **TxxV** | **GGDEF** | **Q** | **EALxR** | **N** | **E** | **DDFGTGYSSL** | **K** | **E** | **Q** |
| **WP_045581444** | **581** |  | **PAS** | **DxDxFAxxRSxxGQxxAN** | **SxxT** | **SDHAF** | **Q** | **EALxR** | **N** | **E** | **DDFGTGYSSL** | **K** | **E** | **Q** |
| **WP_045582112** | **751** |  | **PAS** | **DxDxFSxxNExxGFxxGD** | **MxxR** | **AADEF** | **Q** | **EALxR** | **N** | **E** | **DDFGSGYSSL** | **K** | **E** | **Q** |
| **WP_045581135** | **624** |  |  | **DxDxFKxxNDxxGFxxGD** | **TxxA** | **GGDDF** |  | **EALxR** | **N** | **E** | **DDFGVGHSGL** | **K** | **E** | **Q** |

**G. 1. The GGDEF proteins found in *A. halopraeferens* genome**

| **LOCUS TAG** | **AA** | **TM DOMAIN** | **SENSING DOMAINS** | **GGDEF DOMAIN** | | |
| --- | --- | --- | --- | --- | --- | --- |
|  |  |  |  | **ACTIVE SITE 2** | **INHIBITORY SITE** | **ACTIVE SITE 1** |
| **WP_029007018** | **470** |  | **2 REC** | **DxDxFKxxNDxxGHxxGD** | **RxxD** | **GGEEF** |
| **WP_029007170** | **441** |  | **2 PAS** | **DxDxFKxxNDxxGHxxGD** | **RxxD** | **GGEEF** |
| **WP_051341157** | **596** |  | **HAMP /PAS** | **DxDxFKxxNDxxGHxxGD** | **RxxD** | **GGEEF** |
| **WP_029010874** | **304** |  | **1 REC** | **DxDxFKxxNDxxGHxxGD** | **RxxD** | **GGEEF** |
| **WP_084536916** | **369** |  | **1 PAS** | **DxDxFKxxNDxxGHxxGD** | **RxxD** | **GGEEF** |
| **WP_051340577** | **379** | **5** |  | **DxDxFKxxNDxxGHxxGD** | **RxxD** | **GGEEF** |
| **WP_051340868** | **428** | **2** | **HAMP /PAS** | **DxDxFKxxNDxxGHxxGD** | **RxxD** | **GGDEF** |
| **WP_084536527** | **415** | **6** |  | **DxDxFKxxNDxxGHxxGD** | **RxxD** | **GGEEF** |
| **WP_029007913** | **292** |  |  | **DxDxFKxxNDxxGHxxGD** | **RxxD** | **GGEEF** |
| **WP_051341118** | **326** |  |  | **DxDxFKxxNDxxGHxxGD** | **KxxD** | **GGEEF** |
| **WP_084536339** | **221** |  |  | **DxDxFKxxNDxxGHxxGD** | **RxxD** | **GGDEF** |
| **WP_084536494** | **479** |  | **Protoglobin** | **DxDxFKxxNDxxGHxxGD** | **RxxD** | **GGEEF** |
| **WP_051341331** | **344** |  | **REC** | **DxDxFKxxNDxxGHxxGD** | **RxxD** | **GGEEF** |
| **WP_051340956** | **446** |  | **REC/ PAS** | **DxDxFKxxNDxxGHxxGD** | **RxxD** | **GGDEF** |
| **WP_084536514** | **527** |  | **PAS/ GAF** | **DxDxFKxxNDxxGHxxGD** | **RxxD** | **GGDEF** |
| **WP_029010314** | **302** |  | **REC** | **DxDxFKxxNDxxGHxxGD** | **RxxD** | **GGEEF** |
| **WP_029010818** | **392** | **5** |  | **DxDxFKxxNDxxGHxxGD** | **RxxD** | **GGEEF** |
| **WP_051340880** | **763** | **2** | **2 PAS** | **DxDxFKxxNDxxGHxxGD** | **RxxD** | **GGEEF** |
| **WP_084536553** | **587** |  | **HPT/ 2 REC** | **DxDxFKxxNDxxGHxxGD** | **RxxD** | **GGDEF** |
| **WP_051340793** | **741** | **3** | **2 PAS** | **DxDxFKxxNDxxGHxxGD** | **RxxD** | **GGEEF** |

**G. 2. The EAL proteins found in *A. halopraeferens* genome.**

| **LOCUS TAG** | **AA** | **TM DOMAIN** | **SENSING DOMAINS** | **EAL DOMAIN** | | | | | | | |
| --- | --- | --- | --- | --- | --- | --- | --- | --- | --- | --- | --- |
|  |  |  |  | **GMP-BS1** | **MBS1** | **MBS2** | **MBS3** | **LOOP 6** | **H_2_O BS** | **MBS4** | **GMP-BS2** |
| **WP_029006916** | **267** |  |  | **Q** | **EALxR** | **N** | **E** | **DDFGTGYSSL** | **K** | **E** | **Q** |
| **WP_051341148** | **557** |  | **PAS** | **Q** | **EALxR** | **N** | **E** | **DDVGAGTTSF** | **K** | **E** | **Q** |
| **WP_084536631** | **583** |  | **PAS** | **Q** | **EALxR** | **N** | **E** | **DDVGSGSTSF** | **K** | **E** | **Q** |
| **WP_035693714** | **406** |  | **2** | **Q** | **EVYxR** | **N** | **E** | **DQVRDLDAID** | **K** | **E** | **Q** |

**G. 3. The Hybrid proteins found in *A. halopraeferens* genome**

| **LOCUS TAG** | **AA** | **TM DOMAIN** | **SENSING DOMAINS** | **GGDEF DOMAIN** | | | **EAL DOMAIN** | | | | | | | |
| --- | --- | --- | --- | --- | --- | --- | --- | --- | --- | --- | --- | --- | --- | --- |
|  |  |  |  | **ACTIVE SITE 2** | **INHIBITORY SITE** | **ACTIVE SITE 1** | **GMP-BS1** | **MBS1** | **MBS2** | **MBS3** | **LOOP 6** | **H_2_O BS** | **MBS4** | **GMP-BS2** |
| **WP_051341020** | **1266** | **11** | **HAMP /3 PAS** | **DxDxFKxxNDxxGHxxGD** | **RxxD** | **GGDEF** | **Q** | **EALxR** | **N** | **E** | **DDFGKGYSSL** | **K** | **E** | **Q** |
| **WP_051340616** | **853** |  | **3 CBS** | **DxDxFKxxNDxxGHxxGD** | **GxxD** | **GGDEF** | **Q** | **EALxR** | **N** | **E** | **DDFGTRQSCL** | **K** | **E** | **Q** |
| **WP_051341097** | **952** | **2** | **2 PAS** | **DxDxFKxxNDxxGHxxGD** | **RxxD** | **GGDEF** | **Q** | **EALxR** | **N** | **E** | **DDFGTGYSSL** | **K** | **E** | **Q** |
| **WP_051340993** | **880** |  | **3PAS** | **DxDxFKxxNDxxGHxxGD** | **RxxD** | **GGDEF** | **Q** | **EALxR** | **N** | **E** | **DDFGTGYSSL** | **K** | **E** | **Q** |
| **WP_084536765** | **1056** | **2** | **2CACHE** | **DxDxFKxxNDxxGHxxGD** | **RxxD** | **GGDEF** | **Q** | **EALxR** | **N** | **E** | **DDFGTGYSSL** | **K** | **E** | **Q** |
| **WP_051340400** | **994** | **1** | **HAMP/2PAS** | **DxDxFKxxNDxxGHxxGD** | **RxxD** | **GVDEF** | **Q** | **EALxR** | **N** | **E** | **DDFGTGHSSL** | **K** | **E** | **Q** |
| **WP_029006814** | **835** |  | **3PAS** | **DxDxFKxxNDxxGPxxGD** | **RxxD** | **SADEF** | **Q** | **EALxR** | **N** | **E** | **DDFGTGYSSL** | **K** | **E** | **Q** |
| **WP_035694205** | **685** |  | **2PAS** | **DxNxFKxxNDxxGHxxGD** | **RxxD** | **GADEF** | **Q** | **EALxR** | **N** | **E** | **DDFGTGYSSL** | **K** | **E** | **Q** |
| **WP_051340475** | **741** |  |  | **DxDxFRxxNExxGQxxGD** | **RxxD** | **ASDEF** | **Q** | **EALxR** | **N** | **E** | **DDFGTGFSSL** | **K** | **E** | **Q** |
| **WP_029008624** | **774** |  | **REC** | **DxDxFQxxNDxxGHxxGD** | **PxxA** | **TGDTF** | **Q** | **EALxR** | **N** | **E** | **DDFGTGHSSL** | **K** | **E** | **Q** |
| **WP_051340933** | **982** |  | **PAS** | **DxDxFKxxNDxxGHxxGD** | **Gxx-** | **GGDEF** | **Q** | **EALxR** | **N** | **E** | **DDFGTGYSSL** | **K** | **E** | **Q** |
| **WP_084536394** | **690** |  | **PAS** | **DxDxFSxxNExxGFxxGD** | **PxxT** | **AADEF** | **Q** | **EGLxR** | **N** | **E** | **DDFGSGYSSL** | **K** | **E** | **Q** |
| **WP_029010954** | **581** |  | **PAS** | **DxDxFVxxRTxxGQxxAN** | **GxxD** | **SDHAF** | **Q** | **EALxR** | **N** | **E** | **DDFGTGYSSL** | **K** | **E** | **Q** |
| **WP_051340250** | **911** |  | **PBD/PAS** | **GxDxFVxxNExxGQxxGD** | **DxxD** | **TGDEF** | **Q** | **EALxR** | **N** | **E** | **DDFGTGYSSL** | **K** | **E** | **Q** |

**H. 1. The GGDEF proteins found in *A. oryzae* genome**

| **LOCUS TAG** | **AA** | **TM DOMAIN** | **SENSING DOMAINS** | **GGDEF DOMAIN** | | |
| --- | --- | --- | --- | --- | --- | --- |
|  |  |  |  | **ACTIVE SITE 2** | **INHIBITORY SITE** | **ACTIVE SITE 1** |
| **WP_085087985** | **573** |  | **PAS/GAF** | **DLDNFKAVNDLHGHQQGD** | **RxxD** | **GGDEF** |
| **WP_085085858** | **563** | **1** | **CACHE_3/HAMP** | **DLDGFKPVNDRLGHDAGD** | **RxxD** | **GGDEF** |
| **WP_085089774** | **238** |  |  | **DLDGFKAINDTHGHVAGD** | **RxxD** | **GGDEF** |
| **WP_085083186** | **552** |  | **3 PAS** | **DLDGFKPVNDRLGHDAGD** | **RxxD** | **GGDEF** |
| **WP_085086986** | **395** | **5** |  | **DLDDFKAVNDRYGHEEGD** | **RxxD** | **GGEEF** |
| **WP_085087587** | **302** |  |  | **DIDKFKGINDSYGHDIGD** | **RxxD** | **GGEEF** |
| **WP_085088695** | **342** |  |  | **DIDHFKAFNDTHGHLVGD** | **KxxD** | **GGEEF** |
| **WP_085089300** | **551** |  | **3 PAS** | **DVDHFKRINDTFGHATGD** | **RxxD** | **GGEEF** |
| **WP_085087084** | **384** | **7** |  | **DLDHFKRVNDRFGHATGD** | **RxxD** | **GGEEF** |
| **WP_085083707** | **397** | **7** |  | **DIDRFKSINDRAGHPFGD** | **RxxD** | **GGEEF** |
| **WP_085087775** | **352** |  | **REC** | **DLDGFKAVNDAHGDTVGD** | **RxxD** | **GGAEF** |
| **WP_085086748** | **468** |  | **PAS/REC** | **DIDHFKRINDELGHAAGD** | **RxxD** | **GGEEF** |
| **WP_085085729** | **262** |  |  | **DIDHFKDVNDAFGHAAGD** | **RxxD** | **GGEEF** |
| **WP_085089772** | **464** |  | **2 REC** | **DIDHFKVVNDTYGHAIGD** | **RxxD** | **GGEEF** |
| **WP_085090164** | **305** |  | **REC** | **DVDHFKAYNDQYGHQAGD** | **RxxD** | **GGEEF** |
| **WP_085087302** | **312** |  | **REC** | **DIDHFKTINDTHGHSNGD** | **RxxD** | **GGEEF** |
| **WP_085089062** | **467** |  | **PAS/REC** | **DIDRFKAINDTHGHAVGD** | **RxxD** | **GGEEF** |
| **WP_085089873** | **304** |  | **REC** | **DIDHFKRVNDTHGHATGD** | **RxxD** | **GGEEF** |
| **WP_085084481** | **378** | **5** |  | **DIDHFKQVNDSRGHDAGD** | **RxxD** | **GGEEF** |

**H. 2. The EAL proteins found in *A. oryzae* genome**

| **LOCUS TAG** | **AA** | **TM DOMAIN** | **SENSING DOMAINS** | **EAL DOMAIN** | | | | | | | |
| --- | --- | --- | --- | --- | --- | --- | --- | --- | --- | --- | --- |
|  |  |  |  | **GMP-BS1** | **MBS1** | **MBS2** | **MBS3** | **LOOP 6** | **H_2_O BS** | **MBS4** | **GMP-BS2** |
| **WP_085089859** | **292** |  |  | **Q** | **EALVR** | **N** | **E** | **DDFGSGYSGL** | **K** | **E** | **Q** |
| **WP_085090217** | **454** | **2** |  | **Q** | **EVFxR** | **N** | **E** | **DQVTDLAIDL** | **K** | **E** | **Q** |
| **WP_085089651** | **585** |  | **PAS** | **Q** | **EALAR** | **N** | **E** | **DDFGAGAASF** | **K** | **E** | **Q** |
| **SMF76274** | **253** |  |  | **Q** | **EALTR** | **N** | **E** | **DDVGAGSTSF** | **K** | **E** | **Q** |

| **LOCUS TAG** | **AA** | **TM DOMAIN** | **SENSING DOMAINS** | **GGDEF DOMAIN** | | | **EAL DOMAIN** | | | | | | | |
| --- | --- | --- | --- | --- | --- | --- | --- | --- | --- | --- | --- | --- | --- | --- |
|  |  |  |  | **ACTIVE SITE 2** | **INHIBITORY SITE** | **ACTIVE SITE 1** | **GMP-BS1** | **MBS1** | **MBS2** | **MBS3** | **LOOP 6** | **H_2_O BS** | **MBS4** | **GMP-BS2** |
| **WP_085090348** | **751** |  | **PAS/GAF** | **DxDxFKxxNDxxGHxxGD** | **IxxD** | **GGDEF** | **Q** | **EALxR** | **N** | **E** | **DDFGTGYSSL** | **K** | **E** | **Q** |
| **WP_085090751** | **881** | **1** | **PAS/PBpb** | **GxDxFKxxNDxxGHxxGD** | **RxxD** | **GGDEF** | **Q** | **EALxR** | **N** | **E** | **DDFGTGYSNL** | **K** | **E** | **Q** |
| **WP_085090847** | **935** | **1** | **PAS/CHASE** | **DxDxFKxxNDxxGHxxGD** | **RxxD** | **GGDEF** | **Q** | **EALxR** | **N** | **E** | **DDFGTGYSSL** | **K** | **E** | **Q** |
| **WP_085088261** | **1090** | **2** | **2 PAS/ 2 CACHE_2** | **DxDxFKxxNDxxGHxxGD** | **RxxD** | **GGDEF** | **Q** | **EALxR** | **N** | **E** | **DDFGTGYSSL** | **K** | **E** | **Q** |
| **WP_085086601** | **685** |  | **2 PAS** | **DxNxFKxxNDxxGHxxGD** | **RxxD** | **GGDEF** | **Q** | **EALxR** | **N** | **E** | **DDFGTGYSSL** | **K** | **E** | **Q** |
| **WP_085087548** | **1007** | **1** | **PAS** | **DxDxFKxxNDxxGHxxGD** | **GxxL** | **GGDEF** | **Q** | **EALxR** | **N** | **E** | **DDFGTGYSSL** | **K** | **E** | **Q** |
| **WP_085085664** | **1004** | **7** | **2 PAS** | **DxDxFKxxNDxxGHxxGD** | **RxxD** | **SGDEF** | **Q** | **EALxR** | **N** | **E** | **DDFGTGFSSL** | **K** | **E** | **Q** |
| **WP_085092309** | **692** | **2** | **HAMP** | **DxDxFKxxNDxxGHxxGD** | **RxxD** | **GGDEF** | **Q** | **EALxR** | **N** | **E** | **DDFGTGYSSL** | **K** | **E** | **Q** |
| **WP_085090440** | **789** | **1** | **PAS/HAMP** | **DxSxFKxxTDxxGHxxGD** | **RxxD** | **GTDDF** | **Q** | **EALxR** | **N** | **E** | **DDFGTGHSSL** | **K** | **E** | **Q** |
| **WP_085089727** | **848** |  | **3 PAS** | **DxDxFKxxNDxxGSxxGD** | **RxxD** | **SADEF** | **Q** | **EALxR** | **N** | **E** | **DDFGTGYSSL** | **K** | **E** | **Q** |
| **WP_085091422** | **873** | **2** | **PAS/HAMP** | **DxDxFKxxNDxxGHxxGD** | **VxxG** | **GGDEF** | **Q** | **EALxR** | **N** | **E** | **DDFGTGYSSL** | **K** | **E** | **Q** |
| **WP_085089560** | **700** |  | **2 PAS** | **DxDxFSxxNExxGFxxGD** | **ExxT** | **AADEF** | **Q** | **EALxR** | **N** | **E** | **DDFGSGYSSL** | **K** | **E** | **Q** |
| **WP_085090458** | **581** |  | **PAS** | **DxDxFAxxRSxxGQxxAN** | **AxxD** | **SDHAF** | **Q** | **EALxR** | **N** | **E** | **DDFGTGYSSL** | **K** | **E** | **Q** |
| **WP_085084529** | **645** |  | **GAF** | **AxQxYAxxTRxxGQxxAD** | **GxxS** | **SPARF** | **Q** | **EALxR** | **N** | **E** | **DDFGTGQSAL** | **K** | **E** | **Q** |
| **WP_085087658** | **622** |  |  | **DxDxFKxxNDxxGFxxGD** | **CxxG** | **GGDDF** | **Q** | **EALxR** | **N** | **E** | **DDFGVGHSGL** | **K** | **E** | **Q** |

**H. 3. The Hybrid proteins found in *A. oryzae* genome**

**I. 1. The GGDEF proteins found in *A. humicireducens* genome**

| **LOCUS TAG** | **AA** | **TM DOMAIN** | **SENSING DOMAINS** | **GGDEF DOMAIN** | | |
| --- | --- | --- | --- | --- | --- | --- |
|  |  |  |  | **ACTIVE SITE 2** | **INHIBITORY SITE** | **ACTIVE SITE 1** |
| **WP_063634236** | **466** |  | **PAS/REC** | **DxDxFKxxNDxxGHxxGD** | **RxxD** | **GGEEF** |
| **WP_063634866** | **464** |  | **2 REC** | **DxDxFKxxNDxxGHxxGD** | **RxxD** | **GGEEF** |
| **WP_063634969** | **304** |  | **REC** | **DxDxFKxxNDxxGHxxGD** | **RxxD** | **GGEEF** |
| **WP_063634076** | **342** |  |  | **DxDxFKxxNDxxGHxxGD** | **RxxA** | **GGEEF** |
| **WP_063633816** | **580** |  | **PAS/GAF** | **DxDxFKxxNDxxGHxxGD** | **RxxD** | **GGEEF** |
| **WP_063635604** | **396** | **5** |  | **DxDxFKxxNDxxGHxxGD** | **RxxD** | **GGEEF** |
| **WP_063635255** | **305** |  | **REC** | **DxDxFKxxNDxxGHxxGD** | **RxxD** | **GGEEF** |
| **WP_082860748** | **306** |  |  | **DxDxFKxxNDxxGHxxGD** | **RxxD** | **GGEEF** |
| **WP_063633748** | **357** |  | **REC** | **DxDxFKxxNDxxGDxxGD** | **RxxD** | **GGQEF** |
| **WP_082860663** | **308** |  | **REC** | **DxDxFKxxNDxxGHxxGD** | **PxxG** | **GGEEF** |
| **WP_063635416** | **302** |  |  | **DxDxFKxxNDxxGHxxGD** | **RxxD** | **GGDEF** |
| **WP_063635983** | **555** |  | **2 PAS** | **DxDxFKxxNDxxGHxxGD** | **RxxD** | **GGEEF** |
| **WP_063634868** | **238** |  |  | **DxDxFKxxNDxxGHxxGD** | **RxxD** | **GGDEF** |
| **WP_082860790** | **438** | **7** |  | **DxDxFKxxNDxxGHxxGD** | **RxxD** | **GGEEF** |

**I. 2. The EAL proteins found in *A. humicireducens* genome**

| **LOCUS TAG** | **AA** | **TM DOMAIN** | **SENSING DOMAINS** | **EAL DOMAIN** | | | | | | | |
| --- | --- | --- | --- | --- | --- | --- | --- | --- | --- | --- | --- |
|  |  |  |  | **GMP-BS1** | **MBS1** | **MBS2** | **MBS3** | **LOOP 6** | **H_2_O BS** | **MBS4** | **GMP-BS2** |
| **WP_063636223** | **433** |  |  | **Q** | **EVFxR** | **N** | **E** | **DQVTDLGIDL** | **K** | **E** | **Q** |
| **WP_063634480** | **252** |  |  | **Q** | **EALxR** | **N** | **E** | **DDFGSGYSGL** | **K** | **E** | **Q** |
| **WP_063634954** | **298** |  |  | **Q** | **EALxR** | **N** | **E** | **DDFGTGWSSL** | **K** | **E** | **Q** |
| **WP_063634607** | **582** |  | **PAS** | **Q** | **EALxR** | **N** | **E** | **DDVGAGSTSF** | **K** | **E** | **Q** |

1. **3. The Hybrid proteins found in *A. humicireducens* genome**

| **LOCUS TAG** | **AA** | **TM DOMAIN** | **SENSING DOMAINS** | **GGDEF DOMAIN** | | | **EAL DOMAIN** | | | | | | | |
| --- | --- | --- | --- | --- | --- | --- | --- | --- | --- | --- | --- | --- | --- | --- |
|  |  |  |  | **ACTIVE SITE 2** | **INHIBITORY SITE** | **ACTIVE SITE 1** | **GMP-BS1** | **MBS1** | **MBS2** | **MBS3** | **LOOP 6** | **H_2_O BS** | **MBS4** | **GMP-BS2** |
| **WP_063635905** | **934** | **1** | **PAS/CHASE** | **DxDxFKxxNDxxGHxxGD** | **RxxD** | **GGDEF** | **Q** | **EALxR** | **N** | **E** | **DDFGTGYSSL** | **K** | **E** | **Q** |
| **WP_063633937** | **1085** | **3** | **2 PAS/2 CACHE** | **DxDxFKxxNDxxGHxxGD** | **RxxD** | **GGDEF** | **Q** | **EALxR** | **N** | **E** | **DDFGTGYSSL** | **K** | **E** | **Q** |
| **WP_063635249** | **713** |  |  | **DxDxFKxxNDxxGHxxGD** | **RxxD** | **GGDEF** | **Q** | **EALxR** | **N** | **E** | **DDFGTGYSSL** | **K** | **E** | **Q** |
| **WP_063633711** | **904** | **2** | **PAS/ PBpb** | **GxDxFKxxNDxxGHxxGD** | **RxxD** | **GGDEF** | **Q** | **EALxR** | **N** | **E** | **DDFGTGYSNL** | **K** | **E** | **Q** |
| **WP_063634828** | **712** |  | **2 PAS** | **DxDxFKxxNDxxGTxxGD** | **RxxD** | **SADEF** | **Q** | **EALxR** | **N** | **E** | **DDFGTGYSSL** | **K** | **E** | **Q** |
| **WP_063633674** | **753** |  | **PAS/GAF** | **DxDxFKxxNDxxGHxxGD** | **IxxD** | **GGDEF** | **Q** | **EALxR** | **N** | **E** | **DDFGTGYSSL** | **K** | **E** | **Q** |
| **WP_063633591** | **1006** | **1** | **PAS** | **DxDxFKxxNDxxGHxxGD** | **GxxL** | **GGDEF** | **Q** | **EALIR** | **N** | **E** | **DDFGTGYSSL** | **K** | **E** | **Q** |
| **WP_063635491** | **581** |  | **PAS** | **DxDxFAxxRSxxGQxxAN** | **AxxD** | **SDHAF** | **Q** | **EALxR** | **N** | **E** | **DDFGTGYSSL** | **K** | **E** | **Q** |
| **WP_063634303** | **729** |  | **2PAS** | **DxDxFSxxNExxGFxxGD** | **PxxT** | **AADEF** | **Q** | **EALxR** | **N** | **E** | **DDFGSGYSSL** | **K** | **E** | **Q** |
| **WP_082860811** | **627** |  | **GAF** | **DxAxSQxAExxRVxxQV** | **PxxT** | **SPARF** | **Q** | **EALxR** | **N** | **E** | **DDFGTGQSAL** | **K** | **E** | **Q** |
| **WP_063635384** | **626** |  |  | **DxDxLVxxIRxxSPxxDI** | **IxxD** | **GGDDF** | **Q** | **EALxR** | **N** | **E** | **DDFGVGHSGL** | **K** | **E** | **Q** |

MBS 1 and MBS2 = metal binding sites in EAL domain; GMP-BS1 and GMP-BS2 = amino acid binding motifs to cyclic-diGMP; Loop = Loop6 from EAL domains. H2O BS, binding site to water; AA, number total of amino acid; xx = whichever amino acid. The domain prediction was performed based on protein sequences derived from the genome sequences of CdgA (Diguanylate cyclase A)[ ] and ChsA (Phosphodiesterase), both of them previously characterized in *A. brasilense* Sp7 strain using the modular architecture research tool (SMART) program. The sensor domains were predicted by SMART are shown as follows. PAS/PAC, represented as PAS fold family; Transmembrane domains, TMD; CACHE 2, Calcium channels and chemotaxis receptor family; REC, Response regulator receiver; HAMP “linker regions” Histidine kinases, Adenyl cyclases, Methyl-accepting chemotaxis proteins and Phosphatases. CHASE, Cyclases, Histidine kinases Associated Sensory Extracellular domain; GAF, cGMP phosphodiesterase, Adenyl cyclase; PBPb, Bacterial extracellular solute-binding proteins; Protoglobine, domain-associated heme. Data extracted from http://blast.ncbi.nlm.nih.gov/Blast.cgi?PAGE=Proteins and http:// and <http://smart.embl-heidelberg.de/>

**Reference.**

1. Römling U, Liang ZX, Dow JM. 2017. Progress in understanding of the molecular basis underlying functional diversification of cyclic di-nucleotide turnover proteins. J. Bacteriol. doi:10.1128/JB.00790-16
2. Ramírez-Mata A, López Lara LI, Xiqui-Vázquez ML, Romero Osorio A, Saúl Jijón-Moreno S, Baca BE. The cyclic-di-GMP diguanylate cyclase CdgA has a role in biofilm formation and exopolysaccharide production in Azospirillum brasilense. Research Microbiol. 2016; doi: 10.1016/j.resmic.2015.12.004.
3. Carreño-López R, Sánchez A, Camargo N, Elmerich C, Baca BE. Characterization of chsA, a new gene controlling the chemotactic response, in Azospir*illum brasilense* Sp7. Arch Microbiol. 2009;191:501-507.
4. Letunic I, Doerks T, Bork P. SMART 7: recent updates to the protein domain annotation resource. Nucleic Acids Res. 2012; 40. D302–D305.
