# Supplementary material for: In silico comparative analysis of GGDEF and EAL domain signaling proteins from the Azospirillum genomes
Source: BMC Microbiol. 2018 Mar 9;18:20. doi: 10.1186/s12866-018-1157-0 (PMC5845226; doi:10.1186/s12866-018-1157-0)
Supplement: Supplementary file 3 — Table S3 and Table S4. Table S3. Repertoire of GGDEF, EAL and GGDEF-EAL predicted proteins, organization, and domain architectures found in the selected Azospirillum spp. genomes. Table S4. Accession numbers of GGDEF, EAL and hybrid proteins encoded by genes of select the analyzed Azospirillum genomes. Data extracted from http://blast.ncbi.nlm.nih.gov/Blast.cgi?PAGE=Proteins and http:// and http://smart.embl-heidelberg.de/. (DOCX 268 kb) [file 12866_2018_1157_MOESM3_ESM.docx]

**Additional material**

***In silico* comparative analysis of GGDEF and EAL domain signaling proteins from the *Azospirillum* spp genomes.**

Alberto Ramírez Mata ^1¶^, César Millán Pacheco ^2¶^, José Francisco Cruz Pérez^1^, and Beatriz E. Baca^1*^.

^1^ Centro de Investigaciones en Ciencias Microbiológicas, Benemérita Universidad Autónoma de Puebla. Edif. Edif. IC11, Ciudad Universitaria, Col. San Manuel Puebla Pue. CP72570 Puebla México.

^2^ Facultad de Farmacia. Universidad Autónoma del Estado de Morelos, Av. Universidad #1001, Col. Chamilpa, C.P. 62209. Morelos México.

**Additional file 3**

**Additional file 3: Table 3S and Table 4S.** Table 3S. Repertoire of GGDEF, EAL and GGDEF-EAL predicted proteins, organization, and domain architectures found in the selected *Azospirillum* spp genomes. Table 4S. Accession numbers of GGDEF, EAL and hybrid proteins encoded by genes of select the analyzed *Azospirillum* genomes.

Putative proteins found in the genomes of the *Azospirillum* spp strains. Predicted proteins that are present only in some genomes

from the analyzed strains. Schematic representation of domain organization of containing proteins found in genomes. The domain

prediction was performed based on protein sequences derived from the genome sequences of CdgA (Diguanylate cyclase A) and

ChsA (Phosphodiesterase), both of them previously characterized in *A. brasilense* Sp7 strain using the modular architecture research

tool (SMART) program. The GGDEF domains are shown in rose color, and the EAL domains are shown in blue color. The sensor

domains were predicted by SMART are shown as follows. PAS/PAC, represented as PAS fold family (green); Transmembrane domains,

TMD (grey); CACHE 2, Calcium channels and chemotaxis receptor family (pale blue). REC, Response regulator receiver (blue);

HAMP “linker regions” Histidine kinases, Adenyl cyclases, Methyl-accepting chemotaxis proteins and Phosphatases, (brunet).

CHASE, Cyclases, Histidine kinases Associated Sensory Extracellular domain (orange); GAF, cGMP phosphodiesterase, Adenyl cyclase,

FhlA domain (rose) The FhlA-C domain is active in ATP hydrolysis and activation of transcription. PBPb, Bacterial extracellular

solute-binding proteins, family (brunet); and the rose boxes represent the predicted coiled-coil motifs. The coiled coil is a common

structural motif, formed by approximately 3 ± 5% of all aminoacids in proteins. Typically, it consists of two to five α-helices wrapped around

each ofother into a left-handed helix to form a supercoil. Data extracted from <http://blast.ncbi.nlm.nih.gov/Blast.cgi?PAGE=Proteins> http://

and http:// and <http://smart.embl-heidelberg.de/>. It is indicated a – for Not Found.

**Table S3.** Repertoire of GGDEF, EAL and GGDEF-EAL predicted proteins, organization, and domain architectures

retrieved in select the *Azospirillum* ssp genomes.

| **DOMAIN ARCHITECTURE** | 1. ***brasilense* Sp245** | 1. ***brasilense* Sp7** | 1. ***brasilense* Az39** | 1. ***lipoferum* 4B** | ***A.zospirillum* B510** | ***A. thiophilum*** | ***A. halopraeferens*** | ***A. oryzae*** | ***A. humicireducens*** |
| --- | --- | --- | --- | --- | --- | --- | --- | --- | --- |
| **GGDEF** | | | | | |  |  |  |  |
| **GGDEF**  **REC**  **1. WspR family** | * | * | * | – | – | – | – | – | – |
| **REC**  **GGDEF**  **GAF**  **2.** | * | ***** | * | – | – | – | – | – | – |
| **GGDEF**  **GAF**  **3.** | * | * | * | – | * | – | – | – | – |
| **CACHE**  **GGDEF**  **4.** | * | * | * | – | * | – | – | – | – |
| **PAS**  **GGDEF**  **5.** | * | * | * | – | – | – | * | – | – |
| **GGDEF**  **PAS**  **PAS**  **6.** | * | * | * | – | – | – | – | – | – |
| **PAS**  **GGDEF**  **7.** | * | * | * | – | – | – | – | – | – |
| **CACHE**  **HAMP**  **GGDEF**  **8.** | * | * | * | – | – | – | – | – | – |
| **CACHE**  **GGDEF**  **HAMP**  **9.** | * | * | * | – | – | – | – | – | – |
| **Protoglobine**  **GGDEF**  **10.** | * | * | * | – | – | – | * | – | – |
| **GGDEF**  **11.** | – | * | * | – | – | – | – | – | – |
| **HPT**  **REC**  **REC**  **GGDEF**  **12.** | * | – | – | – | – | – | * | – | – |
| **GGDEF**  **13.** | – | – | – | * | * | * | * | * | – |
| **GGDEF**  **14.** | – | – | – | * | * | – | – | * | – |
| **CACHE**  **GGDEF**  **PAS**  **15.** | – | – | – | * | * | – | – | – | – |
| **GGDEF**  **REC**  **16. WspR family** | – | – | – | * | * | * | * | * | – |
| **GGDEF**  **REC**  **17. WspR family** | – | – | – | * | – | – | – | * | * |
| **GGDEF**  **18.** | – | – | – | * | * | * | – | * | – |
| **GGDEF**  **PAS**  **PAS**  **PAS**  **19.** | – | – | – | * | – | * | – | – | * |
| **GAF**  **REC**  **GGDEF**  **20.** | – | – | – | * | – | – | – | * | * |
| **REC**  **GGDEF**  **PAS**  **21.** | – | – | – | * | * | * | – | * | – |
| **PBPb**  **GGDEF**  **22.**  **GGDEF** | – | – | – | * | – | * | – | – | – |
| **23.** | – | – | – | – | * | – | – | – | * |
| **GGDEF**  **24.** | – | – | – | – | * | – | – | * | * |
| **HAMP**  **GGDEF**  **25.** | – | – | – | – | – | * | – | * | – |
| **EAL** | | | | | |  |  |  |  |
| **PAS**  **EAL**  **26.** | * | **–** | * | * | * | – | * | – | – |
| **EAL**  **27.** | * | * | * | * | * | * | – | * | * |
| **GGDEF-EAL** | | | | | |  |  |  |  |
| **EAL**  **GGDEF**  **PAS**  **28.** | * | **–** | * | – | * | – | – | – | – |
| **GGDEF**  **EAL**  **29.**  **EAL**  **GGDEF**  **PAS**  **CHASE** | * | * | * | * | * | * | – | * | * |
| **30.** | * | * | * | * | * | * | – | * | * |
| **MHYT**  **GGDEF**  **EAL**  **PAS**  **31.** | * | * | * | – | – | – | – | – | – |
| **REC**  **EAL**  **GGDEF**  **32.** | – | – | – | * | * | – | * | – | – |
| **EAL**  **GGDEF**  **HAMP**  **33.** | – | – | – | * | * | * | – | * | * |
| **PAS**  **GGDEF**  **EAL**  **34.**  **PAS**  **GAF**  **GGDEF**  **EAL** | – | – | – | * | * | * | * | * | * |
| **35.** |  |  |  | * | * | * | ─ | * | * |
| **GAF**  **GGDEF**  **EAL**  **36.** | – | – | – | * | * | – | – | * | * |
| **SBP_bac_3**  **PAS**  **GGDEF**  **EAL**  **37.** | – | – | – | * | * | * | – | * | * |
| **CHASE**  CHASE  **GGDEF**  **EAL**  **38.** | – | – | – | * | * | – | – | – | * |
| **PAS**  **PAS**  **GGDEF**  **EAL**  **39.** | – | – | – | * | – | – | – | * | – |
| **hamp**  **PAS**  **GGDEF**  **EAL**  **40.** | – | – | – | – | * | * | – | * | – |
| **PAS**  **GGDEF**  **EAL**  **HAMP**  **41.** | * | * | * | * | * | * | – | * | – |

Putative proteins found in the genomes of the *Azospirillum* spp strains. Predicted proteins that are present only in some genomes

from the analyzed strains. Schematic representation of domain organization of containing proteins found in genomes. The domain

prediction was performed based on protein sequences derived from the genome sequences of CdgA (Diguanylate cyclase A) and

ChsA (Phosphodiesterase), both of them previously characterized in *A. brasilense* Sp7 strain using the modular architecture research

tool (SMART) program. The GGDEF domains are shown in rose color, and the EAL domains are shown in blue color. The sensor

domains were predicted by SMART are shown as follows. PAS/PAC, represented as PAS fold family (green); Transmembrane domains,

TMD (grey); CACHE 2, Calcium channels and chemotaxis receptor family (pale blue). REC, Response regulator receiver (blue);

HAMP “linker regions” Histidine kinases, Adenyl cyclases, Methyl-accepting chemotaxis proteins and Phosphatases, (brunet).

CHASE, Cyclases, Histidine kinases Associated Sensory Extracellular domain (orange); GAF, cGMP phosphodiesterase, Adenyl cyclase,

FhlA domain (rose) The FhlA-C domain is active in ATP hydrolysis and activation of transcription. PBPb, Bacterial extracellular

solute-binding proteins, family (brunet); and the rose boxes represent the predicted coiled-coil motifs. The coiled coil is a common

structural motif, formed by approximately 3 ± 5% of all aminoacids in proteins. Typically, it consists of two to five α-helices wrapped around

each ofother into a left-handed helix to form a supercoil. Data extracted from <http://blast.ncbi.nlm.nih.gov/Blast.cgi?PAGE=Proteins> http://

and http:// and <http://smart.embl-heidelberg.de/>. It is indicated a – for Not Found.

**Table 4S. Accession numbers of GGDEF, EAL and hybrid proteins encoded by genes of select the analyzed *Azospirillum* genomes.**

| **#** | ***A. brasilense* Sp245** | ***A. brasilense* Sp7** | ***A. brasilense* Az39** | ***A. lipoferum* 4B** | ***Azospirillum* B510** | ***A. thiophilum*** | ***A. halopraeferens*** | ***A.***  ***oryzae*** | ***A.***  ***humicireducens*** |
| --- | --- | --- | --- | --- | --- | --- | --- | --- | --- |
| **GGDEF DOMAINS** | | | | | | | | | |
| **1.** | **WP_014240083** | **WP_035672942** | **WP_038528453** | **–** | **–** | **–** | **–** | **–** | **–** |
| **2.** | **WP_014239104** | **WP_035670844** | **WP_038529941** | **–** | **–** | **–** | **–** | **–** | **–** |
| **3.** | **WP_014200048** | **WP_035682812** | **WP_040138238** | **–** | **WP_012974194** | **–** | **–** | **–** | **–** |
| **4.** | **WP_082188241** | **WP_035678542** | **WP_081863206** | **–** | **WP_042445615** | **–** | **–** | **–** | **–** |
| **5.** | **WP_014238973** | **WP_051140034** | **WP_051658027** | **–** | **–** | **–** | **WP_084536916** | **–** | **–** |
| **6.** | **WP_052584448** | **WP_059399449** | **WP_051658431** | **–** | **–** | **–** | **–** | **–** | **–** |
| **7.** | **WP_014197071** | **WP_051140383** | **WP_051658204** | **–** | **–** | **–** | **–** | **–** | **–** |
| **8.** | **WP_052584421** | **WP_059399097** | **WP_051658417** | **–** | **–** | **–** | **–** | **–** | **–** |
| **9.** | **WP_014197673** | **WP_059399331** | **WP_051658298** | **–** | **–** | **–** | **–** | **–** | **–** |
| **10.** | **WP_014241636** | **WP_035675850** | **WP_038529622** | **–** | **–** | **–** | **WP_084536494** | **–** | **–** |
| **11.** | **–** | **WP_051140397** | **WP_051658186** | **–** | **–** | **–** | **–** | **–** | **–** |
| **12.** | **WP_014198789** | **–** | **–** | **–** | **–** | **–** | **WP_084536553** | **–** | **–** |
| **13.** | **–** | **–** | **–** | **WP_014188334** | **WP_012975963** | **WP_045584724** | **WP_084536527** | **WP_085083707** |  |
| **14.** | **–** | **–** | **–** | **WP_014188845** | **WP_012976244** | **–** | **–** | **WP_085084481** | **–** |
| **15.** | **–** | **–** | **–** | **WP_014189160** | **WP_012976938** | **–** | **–** | **–** | **–** |
| **16.** | **–** | **–** | **–** | **WP_014246736** | **WP_012973038** | **WP_045581088** | **WP_051341331** | **WP_085087775** | **WP_063633748** |
| **17.** | **–** | **–** | **–** | **WP_014246507** | **–** | **–** | **–** | **WP_085087302** | **WP_082860663** |
| **18.** | **–** | **–** | **–** | **WP_014249593** | **WP_012977574** | **WP_082109442** | **–** | **WP_085085729** | **–** |
| **19.** | **–** | **–** | **–** | **WP_014250202** | **–** | **WP_082108992** | **–** | **–** | **WP_063635983** |
| **20.** | **–** | **–** | **–** | **WP_014247337** | **–** | **–** | **–** | **WP_085089062** | **WP_063634236** |
| **21.** | **–** | **–** | **–** | **WP_014249410** | **WP_012977364** | **WP_045583377** | **–** | **WP_085086748** |  |
| **22.** | **–** | **–** | **–** | **WP_014249756** | **–** | **WP_082109427** | **–** | **–** | **–** |
| **23.** | **–** | **–** | **–** |  | **WP_012974295** | **–** | **–** | **–** | **WP_082860748** |
| **24.** |  |  |  |  | **WP_012972725** | **–** | **–** | **WP_085087084** | **WP_082860790** |
| **25.** | **–** | **–** | **–** | **–** | **–** | **WP_045586291** | **–** | **WP_085085858** | **–** |
| **EAL DOMAINS** | | | | | | | | | |
| **26.** | **WP_014242202** | **CAJ18244** | **WP_051658176** | **WP_014249491** | **WP_012977214** | **–** | **WP_051341148** | **–** | **–** |
| **27.** | **WP_014240028** | **WP_03562792** | **WP_038528539** | **WP_044549771** | **WP_012974146** | **WP_082108769** | **–** | **SMF76274** | **WP_063634480** |
| **GGDEF-EAL DOMAINS** | | | | | | | | | |
| **28.** | **WP_014241415** | **–** | **WP_038529454** | **–** | **WP_012975524** | **–** | **–** | **–** | **–** |
| **29.** | **WP_014239107** | **WP_059399067** | **WP_038531496** | **WP_014248850** | **WP_012972982** | **WP_045581135** | **–** | **WP_085087658** | **WP_063635384** |
| **30.** | **WP_014240087** | **WP_051140186** | **WP_063922630** | **WP_014250090** | **WP_063828250** | **WP_045584990** | **–** | **WP_085090847** | **WP_063635905** |
| **31.** | **WP_014199675** | **WP_059399655** | **WP_040138308** | **–** | **–** | **–** | **–** | **–** | **–** |
| **32.** | **–** | **–** | **–** | **WP_014188795** | **WP_042444714** | **–** | **WP_029008624** | **–** | **–** |
| **33.** | **–** | **–** | **–** | **WP_014189550** | **WP_012977851** | **WP_045583898** | **–** | **WP_085092309** | **WP_063635249** |
| **34.** | **–** | **–** | **–** | **WP_014246612** | **WP_012972934** | **WP_045582559** | **WP_051340933** | **WP_085087548** | **WP_063633591** |
| **35.** | **–** | **–** | **–** | **WP_014246659** | **WP_012972922** | **WP_045581669** | **–** | **WP_085090348** | **WP_063633674** |
| **36** | **–** | **–** | **–** | **WP_014188667** | **WP_012976217** | **–** | **–** | **WP_085084529** | **WP_082860811** |
| **37.** | **–** | **–** | **–** | **WP_014246690** | **WP_012975124** | **WP_082108976** | **–** | **WP_085090751** | **WP_063633711** |
| **38.** | **–** | **–** | **–** | **WP_014248694** | **WP_012974978** | **–** | **–** | **–** | **WP_063635249** |
| **39.** | **–** | **–** | **–** | **WP_014249547** | **–** | **–** | **–** | **WP_085085664** | **–** |
| **40.** | **–** | **–** | **–** | **–** | **WP_012976461** | **WP_082109156** | **–** | **WP_085091422** | **–** |
| **41.** | **WP_014241589** | **WP_079285130** | **WP_081862986** | **WP_014248963** | **WP_012972811** | **WP_045581219** | **–** | **WP_085090440** | **–** |

Data extracted from <http://blast.ncbi.nlm.nih.gov/Blast.cgi?PAGE>-Proteins

**References**

1. Ramírez-Mata A, López Lara LI, Xiqui-Vázquez ML, Romero Osorio A, Saúl Jijón-Moreno S, Baca BE. The cyclic-di-GMP

diguanylate cyclase CdgA has a role in biofilm formation and exopolysaccharide production in *Azospirillum brasilense*.

Research Microbiol. 2016; doi: 10.1016/j.resmic.2015.12.004.

1. Carreño-López R, Sánchez A, Camargo N, Elmerich C, Baca BE. Characterization of *chsA*, a new gene controlling

the chemotactic response, in *Azospirillum brasilense* Sp7. Arch Microbiol. 2009;191:501-507.

1. Letunic I, Doerks T, Bork P. SMART 7: recent updates to the protein domain annotation resource. Nucleic Acids Res. 2012; 40.

D302–D305.
