# Supplementary material for: In silico comparative analysis of GGDEF and EAL domain signaling proteins from the Azospirillum genomes
Source: BMC Microbiol. 2018 Mar 9;18:20. doi: 10.1186/s12866-018-1157-0 (PMC5845226; doi:10.1186/s12866-018-1157-0)
Supplement: Supplementary file 4 — Table S5 and Table S6. Table 5. Repertoire of GGDEF and GGDEF-EAL (hybrid) predicted proteins, organization, and domain architectures represented exclusively in the A. halopraeferens, A. thiophilum, and A. oryzae genomes. Table 6. Accession numbers of GGDEF and hybrid proteins encoded by genes exclusively found in A. halopraeferens, A. thiophilum, or A. oryzae genomes. Data extracted from http://blast.ncbi.nlm.nih.gov/Blast.cgi?PAGE=Proteins and http://smart.embl-heidelberg.de/. (DOCX 162 kb) [file 12866_2018_1157_MOESM4_ESM.docx]

**Additional material**

***In silico* comparative analysis of GGDEF and EAL domain signaling proteins from the *Azospirillum* spp genomes.**

Alberto Ramírez Mata ^1¶^, César Millán Pacheco ^2¶^, José Francisco Cruz Pérez^1^, and Beatriz E. Baca^1*^.

^1^ Centro de Investigaciones en Ciencias Microbiológicas, Benemérita Universidad Autónoma de Puebla. Edif. Edif. IC11, Ciudad Universitaria, Col. San Manuel Puebla Pue. CP72570 Puebla México.

^2^ Facultad de Farmacia. Universidad Autónoma del Estado de Morelos, Av. Universidad #1001, Col. Chamilpa, C.P. 62209. Morelos México.

**Additional file 4:**

**Table 5S and Table 6S.** Table 5. Repertoire of GGDEF and GGDEF-EAL (hybrid) predicted proteins, organization, and domain architectures represented exclusively in the *A. halopraeferens, A. thiophilum*, and *A. oryzae* genomes. Table 6. Accession numbers of GGDEF and hybrid proteins encoded by genes exclusively found in *A. halopraeferens, A. thiophilum*, or *A. oryzae* genomes. Data were extracted from <http://blast.ncbi.nlm.nih.gov/Blast.cgi?PAGE=Proteins> http://

| **STRUCTURAL DOMAINS** | 1. ***halopraeferens*** |
| --- | --- |
| **GGDEF** |  |
| **GGDEF**  **HAMP**  **PAS**  **1.**  **GGDEF** | * |
| **2.** | * |
| **REC**  **GGDEF**  **PAS**  **3.**  **PDB**  **GGDEF** | * |
| **PAS**  **PAS**  **4.** | * |
| **PAS**  **GGDEF**  **HAMP**  **5.** | * |
| **GGDEF**  **PAS**  **PAS**  **6.** | * |
| **GGDEF-EAL (HYBRID-PROTEINS)**  **PAS**  **GGDEF**  **EAL** |  |
| **PAS**  **PAS**  **HAMP**  **7.** | * |
| **CBS**  **CBS**  **CBS**  **PAS**  **GGDEF**  **EAL**  **8.**  **PAS**  **GGDEF**  **EAL** | * |
| **PAS**  **9.** | * |
| **PAS**  **PAS**  **PAS**  **GGDEF**  **EAL**  **10.**  **GGDEF**  **EAL** | * |
| **11.** | * |
| **PAS**  **GGDEF**  **EAL**  **HAMP**  **PAS**  **12.** | * |
| **Phosphonate-bd**  **PAS**  **GGDEF**  **EAL**  **13.** | * |
| **STRUCTURAL DOMAIN** | ***A. thiophilum*** |
| **GGDEF** |  |
| **CHASE**  **GAF**  **GGDEF**  **14.** |  |
| **STRUCTURAL DOMAIN** | ***A. oryzae*** |
| **GGDEF** |  |
| **GGDEF**  **PAS**  **PAS**  **PAS**  **15.** | * |
| **GGDEF-EAL (HYBRID-PROTEINS)** |  |
| **EAL**  **GGDEF**  **PAS**  **CHASE**  **16.** | * |
| **GGDEF**  **EAL**  **PAS**  **PAS**  **PAS**  **17.** | * |

**Table 5S.** Repertoire of GGDEF and GGDEF-EAL (hybrid) predicted proteins, organization, and domain architectures represented exclusively in the *A. halopraeferens, A. thiophilum*, and *A. oryzae* genomes.

Schematic representation of domain organization of containing proteins found in genomes. The domain prediction was performed based on protein sequences derived from the genome sequences of CdgA (Diguanylate cyclase A) and ChsA (Phosphodiesterase), both of them previously characterized in *A. brasilense* Sp7 strain using the modular architecture research tool (SMART) program. The GGDEF domains are shown in rose color, and the EAL domains are shown in blue color [1, 2]. The sensor domains were predicted by SMART [3] are shown as follows. PAS/PAC, represented as PAS fold family (green); Transmembrane domains, TMD (grey); REC, Response regulator receiver (pale orange); HAMP “linker regions” Histidine kinases, Adenyl cyclases, Methyl-accepting chemotaxis proteins and Phosphatases, (brunet). PBP, Bacterial extracellular solute-binding proteins, family (blue); CHASE, Cyclases, Histidine kinases Associated Sensory Extracellular domain (orange); GAF, cGMP phosphodiesterase, Adenyl cyclase; GAF (violet); CBS, Cystathionine β-synthase (CBS) domains or CBS motifs are conserved structural domains that are present in proteins binding to adenosine derivatives, metal ions, and nucleic acids (orange); Phosphonate-bd domain solute-binding proteins family, which are part of the transport system for alkylphosphonate (purple). Data were extracted from <http://blast.ncbi.nlm.nih.gov/Blast.cgi?PAGE=Proteins> http://

References

1. Ramírez-Mata A, López Lara LI, Xiqui-Vázquez ML, Romero Osorio A, Saúl Jijón-Moreno S, Baca BE. The cyclic-di-GMP diguanylate cyclase CdgA has a role in biofilm formation and exopolysaccharide production in *Azospirillum brasilense*. Research Microbiol. 2016; doi: 10.1016/j.resmic.2015.12.004.
2. Carreño-López R, Sánchez A, Camargo N, Elmerich C, Baca BE. Characterization of *chsA*, a new gene controlling the chemotactic response, in *Azospirillum brasilense* Sp7. Arch Microbiol. 2009;191:501-507.
3. Letunic I, Doerks T, Bork P. SMART 7: recent updates to the protein domain annotation resource. Nucleic Acids Res. 2012; 40. D302–D305.

**Table 6S**. Accession numbers of GGDEF and hybrid proteins encoded by genes exclusively found in *A.* *halopraeferens, A. thiophilum,* or *A. oryzae* genomes.

| **#** | ***A. halopraeferens*** | ***A. thiophilum*** | ***A. oryzae*** |
| --- | --- | --- | --- |
| **GGDEF DOMAINS** | | | |
| **1.** | **WP_051341157** | **─** | **─** |
| **2.** | **WP_051340577** | **─** | **─** |
| **3.** | **WP_051340956** | **─** | **─** |
| **4.** | **WP_051340880** | **─** | **─** |
| **5.** | **WP_051340868** | **─** | **─** |
| **6.** | **WP_051340793** | **─** | **─** |
| **GGDEF-EAL** | | | |
| **7.** | **WP_051341020** | **─** | **─** |
| **8.** | **WP_051340616** | **─** | **─** |
| **9.** | **WP_051341097** | **─** | **─** |
| **10.** | **WP_051340993** | **─** | **─** |
| **11.** | **WP_051340475** | **─** | **─** |
| **12.** | **WP_051340400** | **─** | **─** |
| **13.** | **WP_051340250** | **─** | **─** |
| **GGDEF DOMAINS** | | | |
| **14.** |  | **WP_082108776** |  |
| **15** |  | **─** | **WP_085083186** |
| **GGDEF-EAL** | | | |
| **16.** |  |  | **WP_085084479** |
| **17.** |  |  | **WP_085084613** |

Data were extracted from <http://blast.ncbi.nlm.nih.gov/Blast.cgi?PAGE=Proteins> http://
