# Supplementary material for: In silico comparative analysis of GGDEF and EAL domain signaling proteins from the Azospirillum genomes
Source: BMC Microbiol. 2018 Mar 9;18:20. doi: 10.1186/s12866-018-1157-0 (PMC5845226; doi:10.1186/s12866-018-1157-0)
Supplement: Supplementary file 5 — Table S7. The alignments, root main square deviations (RMSDs) and sequence conservation percentages of proteins with GGD[E]EF, EAL and hybrid domains encoded by genes found in the A. brasilense Sp7 genome. Table 7Sa data including the GGD[E]EF proteins; Table 7Sb, data including the EAL proteins; Table 7Sc and 7Sd, data including the hybrid proteins. Data extracted from http://blast.ncbi.nlm.nih.gov/Blast.cgi?PAGE=Proteins. (DOCX 6071 kb) [file 12866_2018_1157_MOESM5_ESM.docx]

# Additional material:

***In silico* comparative analysis of GGDEF and EAL domain signaling proteins from the *Azospirillum* genomes.**

Alberto Ramírez Mata ^1¶^, César Millán Pacheco ^2¶^, José Francisco Cruz Pérez^1^, Martha Minjárez Sáenz ^1^, and Beatriz E. Baca^1*^.

^1^ Centro de Investigaciones en Ciencias Microbiológicas, Benemérita Universidad Autónoma de Puebla. Edif. Edif. IC11, Ciudad Universitaria, Col. San Manuel Puebla Pue. CP72570 Puebla México.

^2^ Facultad de Farmacia. Universidad Autónoma del Estado de Morelos, Av. Universidad #1001, Col. Chamilpa, C.P. 62209. Morelos México.

**Additional file 5: Table 7S.** The alignments, root main square deviations (RMSD) and sequence conservation percentage of proteins with GGD[E]EF, EAL and hybrid domains encoded by genes found in the *A. brasilense* Sp7 genome. Table 7Sa data including the GGD[E]EF proteins. Table 7Sb data including the EAL proteins. Table 7Sc and 7Sd, data including the hybrid proteins. Data extracted from <http://blast.ncbi.nlm.nih.gov/Blast.cgi?PAGE=Proteins>

**Table 7Sa. The alignments, root main square deviations (RMSD), and** **sequence conservation percentage of the proteins with GGD[E]EF domain, encoded by genes found in the *A. brasilense* Sp7 genome.**


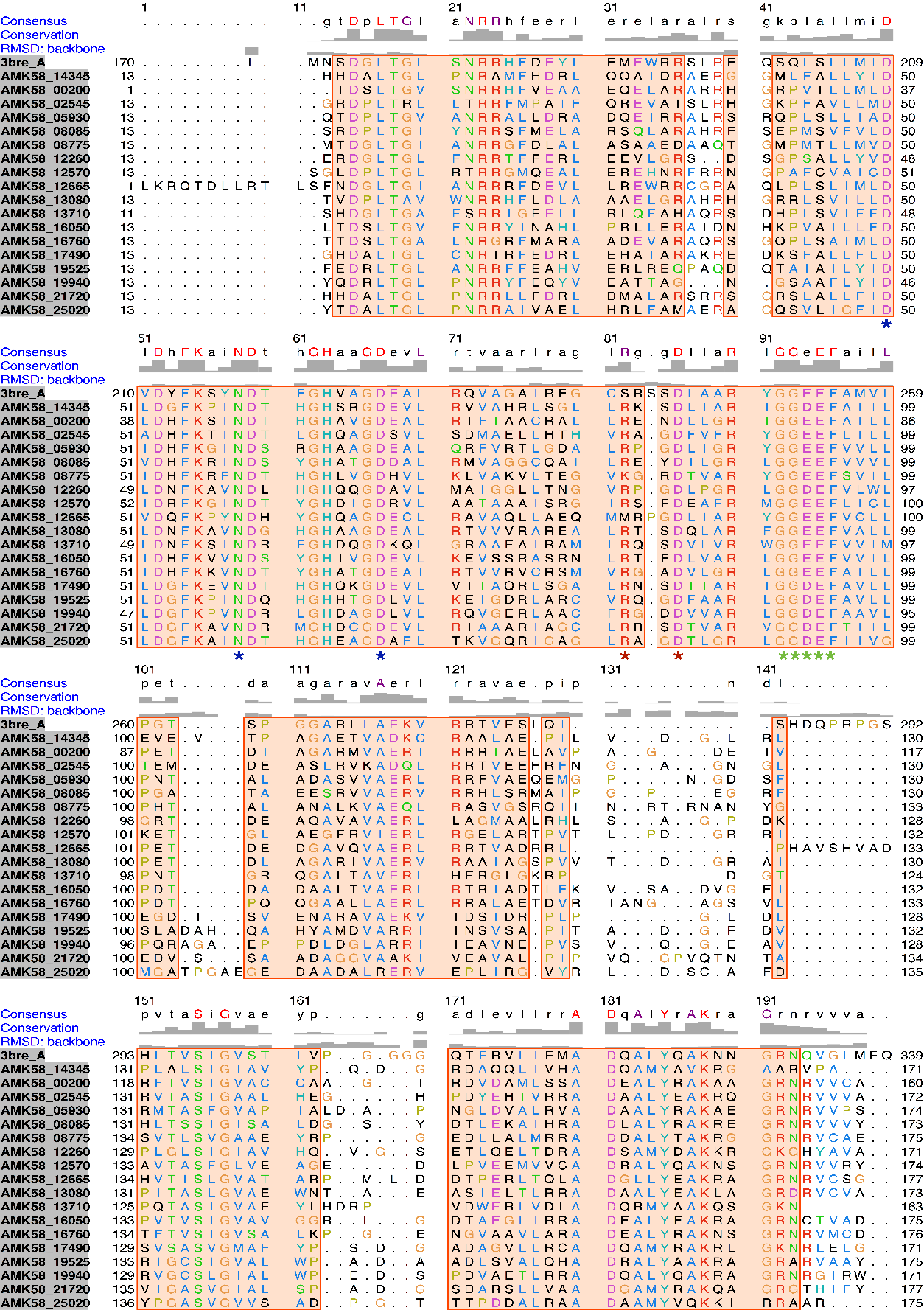

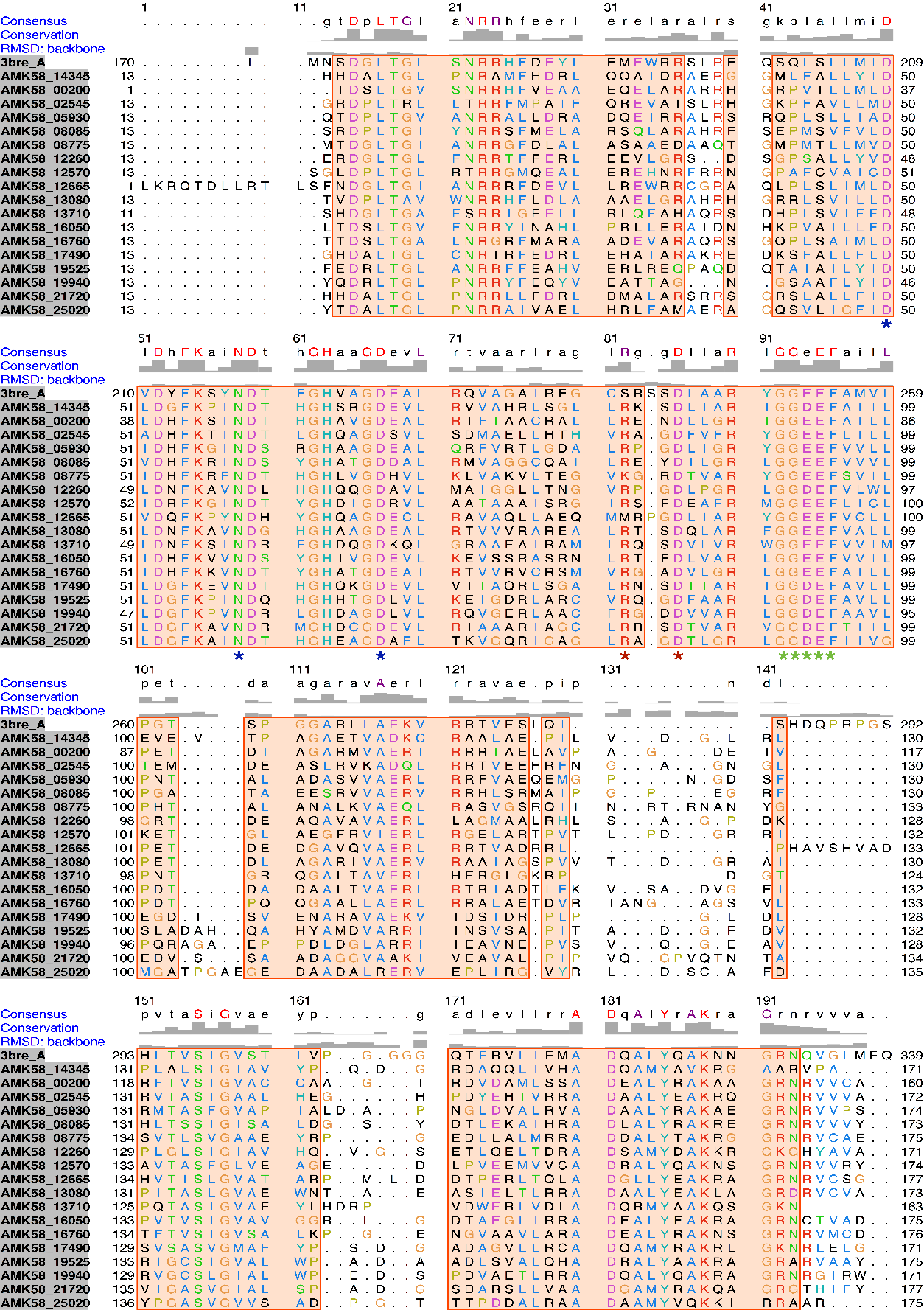


**3bre_A**

**WP_079285130**

**WP_051140034**

**WP_035675850**

**WP_035672942**

**WP_035674663**

**WP_035674304**

**WP_035671267**

**WP_035671094**

**WP_035671042**

**WP_035670844**

**WP_035670654**

**WP_035676633**

**WP_035671246**

**WP_051140383**

**WP_035678542**

**WP_059399331**

**WP_059399449**

**WP_035682812**


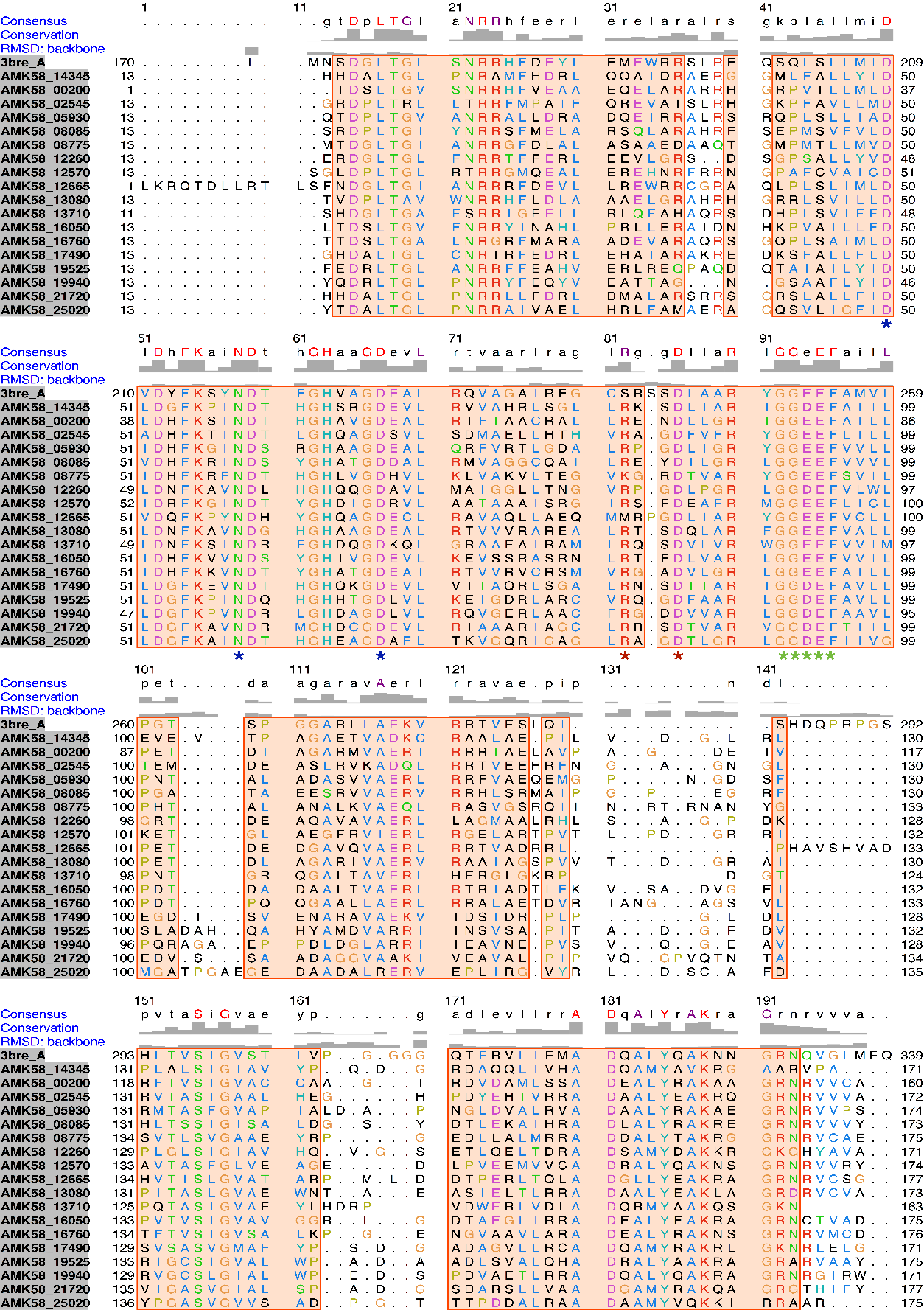


**3bre_A**

**WP_079285130**

**WP_051140034**

**WP_035675850**

**WP_035672942**

**WP_035674663**

**WP_035674304**

**WP_035671267**

**WP_035671094**

**WP_035671042**

**WP_035670844**

**WP_035670654**

**WP_035676633**

**WP_035671246**

**WP_051140383**

**WP_035678542**

**WP_059399331**

**WP_059399449**

**WP_035682812**

**3bre_A**

**WP_079285130**

**WP_051140034**

**WP_035675850**

**WP_035672942**

**WP_035674663**

**WP_035674304**

**WP_035671267**

**WP_035671094**

**WP_035671042**

**WP_035670844**

**WP_035670654**

**WP_035676633**

**WP_035671246**

**WP_051140383**

**WP_035678542**

**WP_059399331**

**WP_059399449**

**WP_035682812**

**3bre_A**

**WP_079285130**

**WP_051140034**

**WP_035675850**

**WP_035672942**

**WP_035674663**

**WP_035674304**

**WP_035671267**

**WP_035671094**

**WP_035671042**

**WP_035670844**

**WP_035670654**

**WP_035676633**

**WP_035671246**

**WP_051140383**

**WP_035678542**

**WP_059399331**

**WP_059399449**

**WP_035682812**


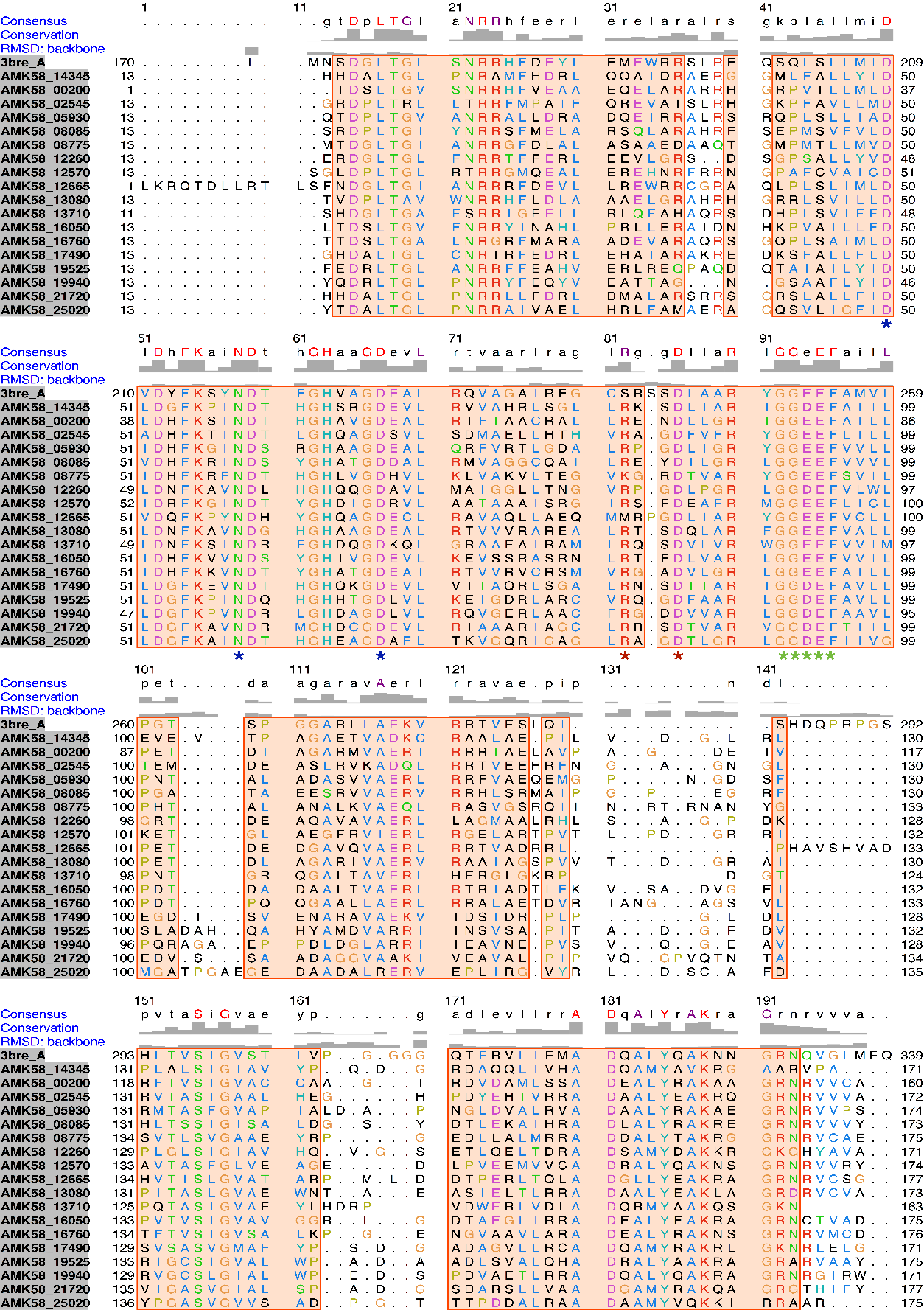

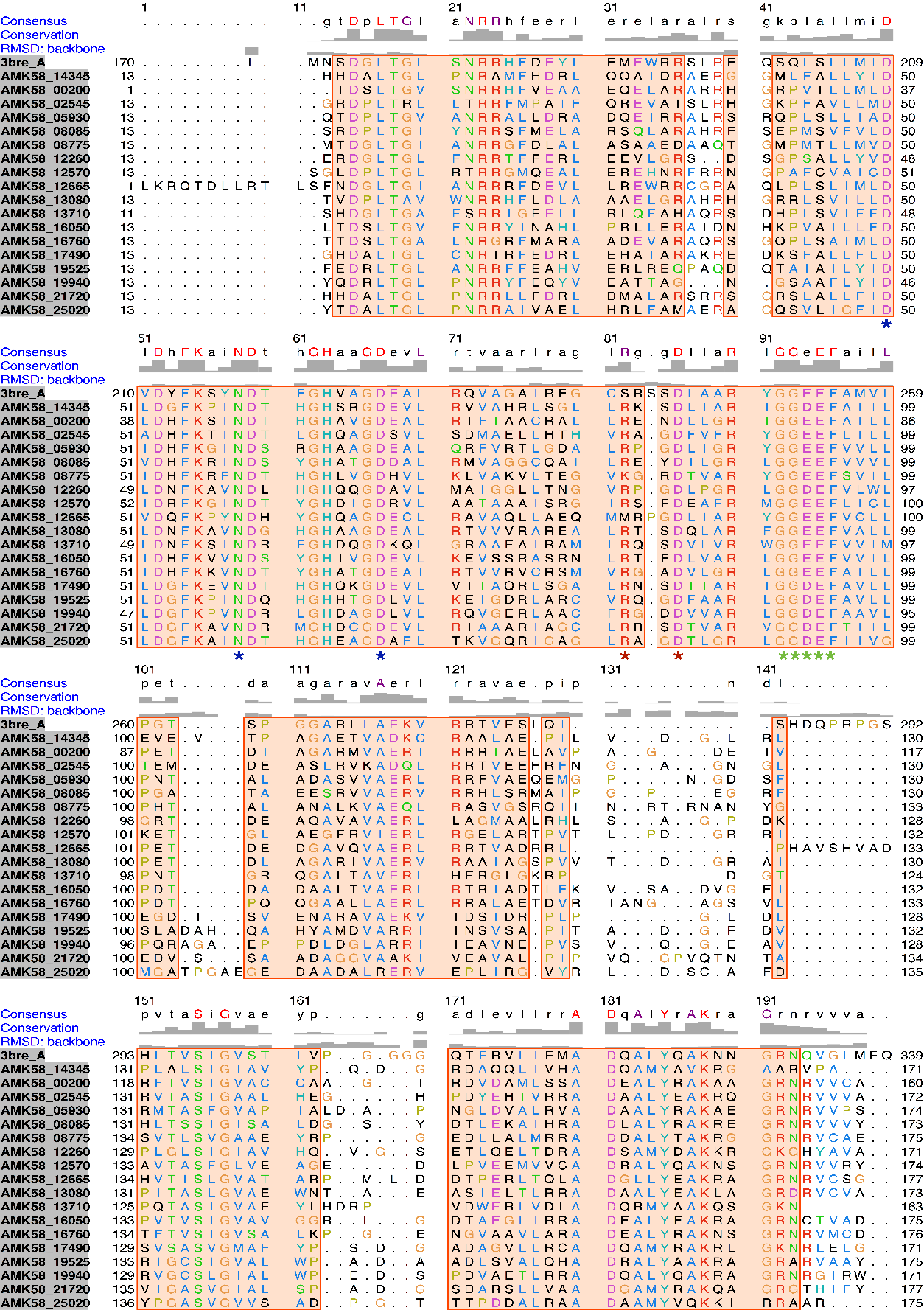


**1: 3bre_A 100.00 36.69 46.88 34.50 38.51 41.62 38.51 35.88 34.10 48.02 40.46 36.42 35.06 41.71 38.82 32.16 35.93 36.00 30.41**

**2** : **WP_079285130** **36.69 100.00 41.18 32.73 33.73 33.13 37.87 39.02 32.34 33.93 37.35 33.33 39.29 31.95 50.89 39.18 40.72 52.63 36.09**

**3: WP_051140034** **46.88 41.18 100.00 42.14 52.50 47.50 41.51 38.22 41.51 44.65 52.50 39.07 45.00 56.25 40.00 38.71 38.41 39.62 36.77**

**4: WP_035675850**  **34.50 32.73 42.14 100.00 39.77 36.26 38.01 35.29 40.12 39.41 39.18 31.06 36.84 36.84 34.73 35.33 37.42 30.99 33.53**

**5: WP_035672942 38.51 33.73 52.50 39.77 100.00 43.35 40.12 34.91 37.21 43.93 46.82 38.89 39.88 47.40 37.50 38.10 32.93 35.47 34.52**

**6: WP_035674663 41.62 33.13 47.50 36.26 43.35 100.00 37.21 34.91 36.63 39.53 41.62 37.89 41.62 44.51 36.31 35.12 33.54 30.23 33.93**

**7: WP_035674304** **38.51 37.87 41.51 38.01 40.12 37.21 100.00 37.87 35.26 42.77 37.79 33.12 43.10 36.57 39.05 36.84 34.13 33.71 30.99**

**8: WP_035671267** **35.88 39.02 38.22 35.29 34.91 34.91 37.87 100.00 30.59 40.24 37.87 33.96 38.46 32.54 37.95 37.95 36.20 37.06 32.53**

**9: WP_035671094 34.10 32.34 41.51 40.12 37.21 36.63 35.26 30.59 100.00 37.21 37.21 33.54 37.57 35.26 33.14 31.95 30.30 33.53 31.36**

**10: WP_035671042 48.02 33.93 44.65 39.41 43.93 39.53 42.77 40.24 37.21 100.00 45.35 37.27 41.62 40.80 36.69 35.88 34.34 33.33 30.59**

**11: WP_035670844 40.46 37.35 52.50 39.18 46.82 41.62 37.79 37.87 37.21 45.35 100.00 38.51 43.93 47.98 39.29 34.52 39.63 39.53 32.74**

**12: WP_035670654 36.42 33.33 39.07 31.06 38.89 37.89 33.12 33.96 33.54 37.27 38.51 100.00 37.27 37.27 36.54 29.49 32.24 35.62 31.45**

**13: WP_035676633** **35.06 39.29 45.00 36.84 39.88 41.62 43.10 38.46 37.57 41.62 43.93 37.27 100.00 40.00 37.28 38.82 36.14 35.63 33.53**

**14: WP_035671246 41.71 31.95 56.25 36.84 47.40 44.51 36.57 32.54 35.26 40.80 47.98 37.27 40.00 100.00 34.32 31.58 34.73 30.86 32.16**

**15: WP_051140383 38.82 50.89 40.00 34.73 37.50 36.31 39.05 37.95 33.14 36.69 39.29 36.54 37.28 34.32 100.00 39.77 40.12 49.71 35.33**

**16: WP_035678542 32.16 39.18 38.71 35.33 38.10 35.12 36.84 37.95 31.95 35.88 34.52 29.49 38.82 31.58 39.77 100.00 50.29 40.46 36.09**

**17: WP_059399331 35.93 40.72 38.41 37.42 32.93 33.54 34.13 36.20 30.30 34.34 39.63 32.24 36.14 34.73 40.12 50.29 100.00 45.56 35.15**

**18: WP_059399449 36.00 52.63 39.62 30.99 35.47 30.23 33.71 37.06 33.53 33.33 39.53 35.62 35.63 30.86 49.71 40.46 45.56 100.00 37.57**

**19: WP_035682812 30.41 36.09 36.77 33.53 34.52 33.93 30.99 32.53 31.36 30.59 32.74 31.45 33.53 32.16 35.33 36.09 35.15 37.57 100.00**

**Table S7b. The alignments, root main square deviations (RMSD), and** **sequence conservation percentage of the hybrid proteins with GGD[E]EF domain, encoded by genes found in the *A. brasilense* Sp7genome.**


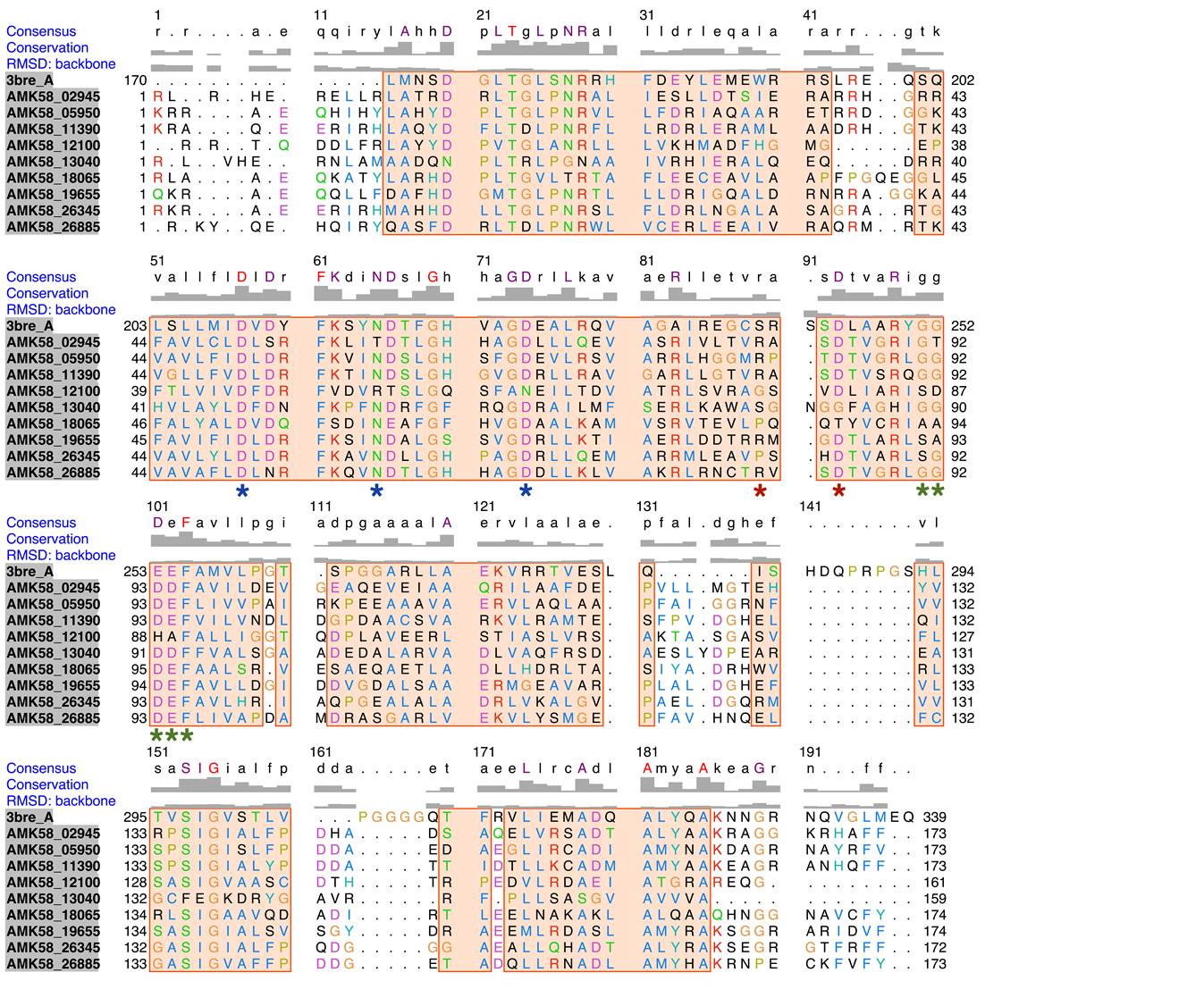


**3bre_A**

**WP_079285130**

**WP_051140186**

**WP_051140104**

**WP_059398931**

**WP_059399067**

**WP_051140628**

**WP_035678503**

**WP_059399655**

**WP_059399677**


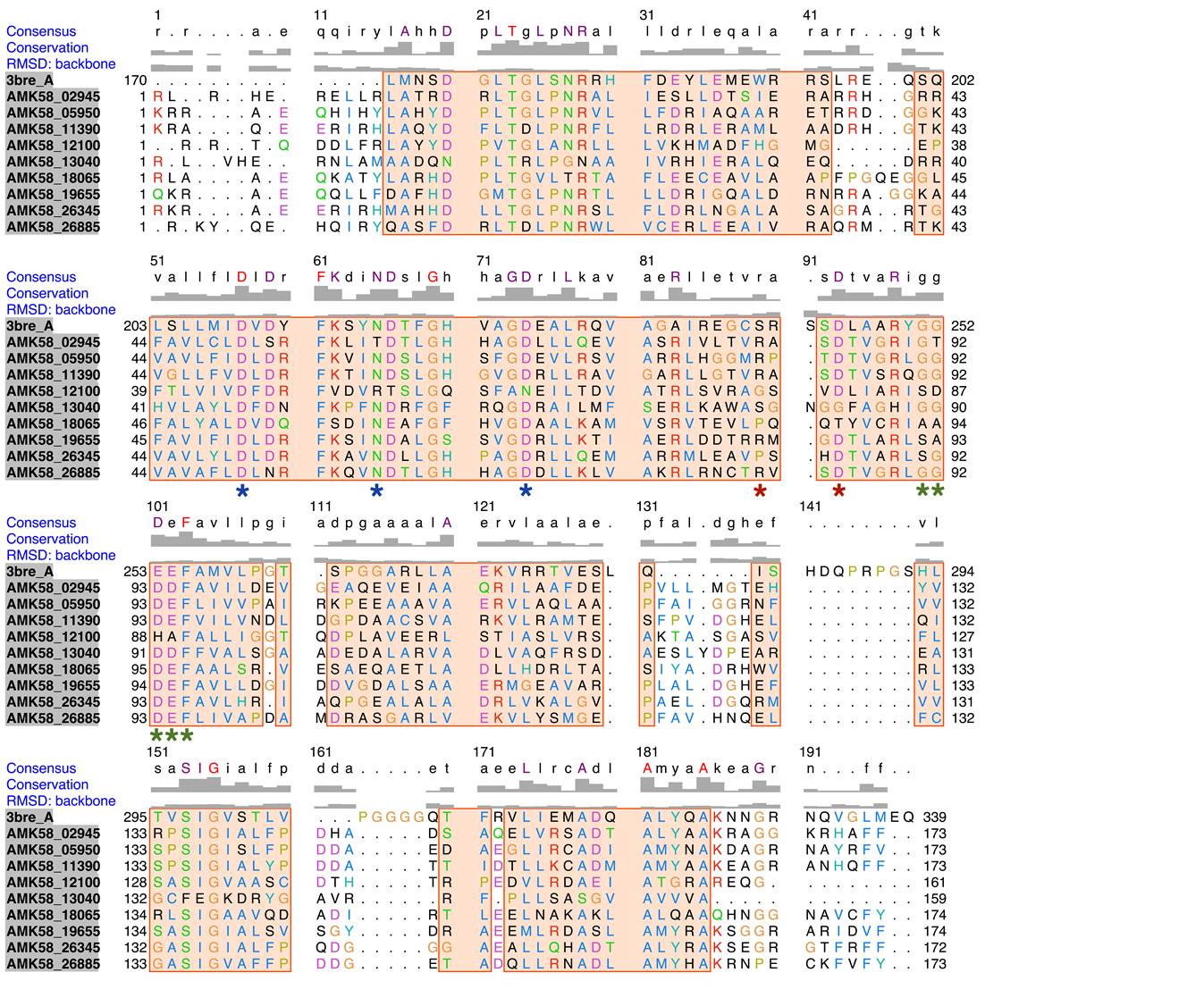

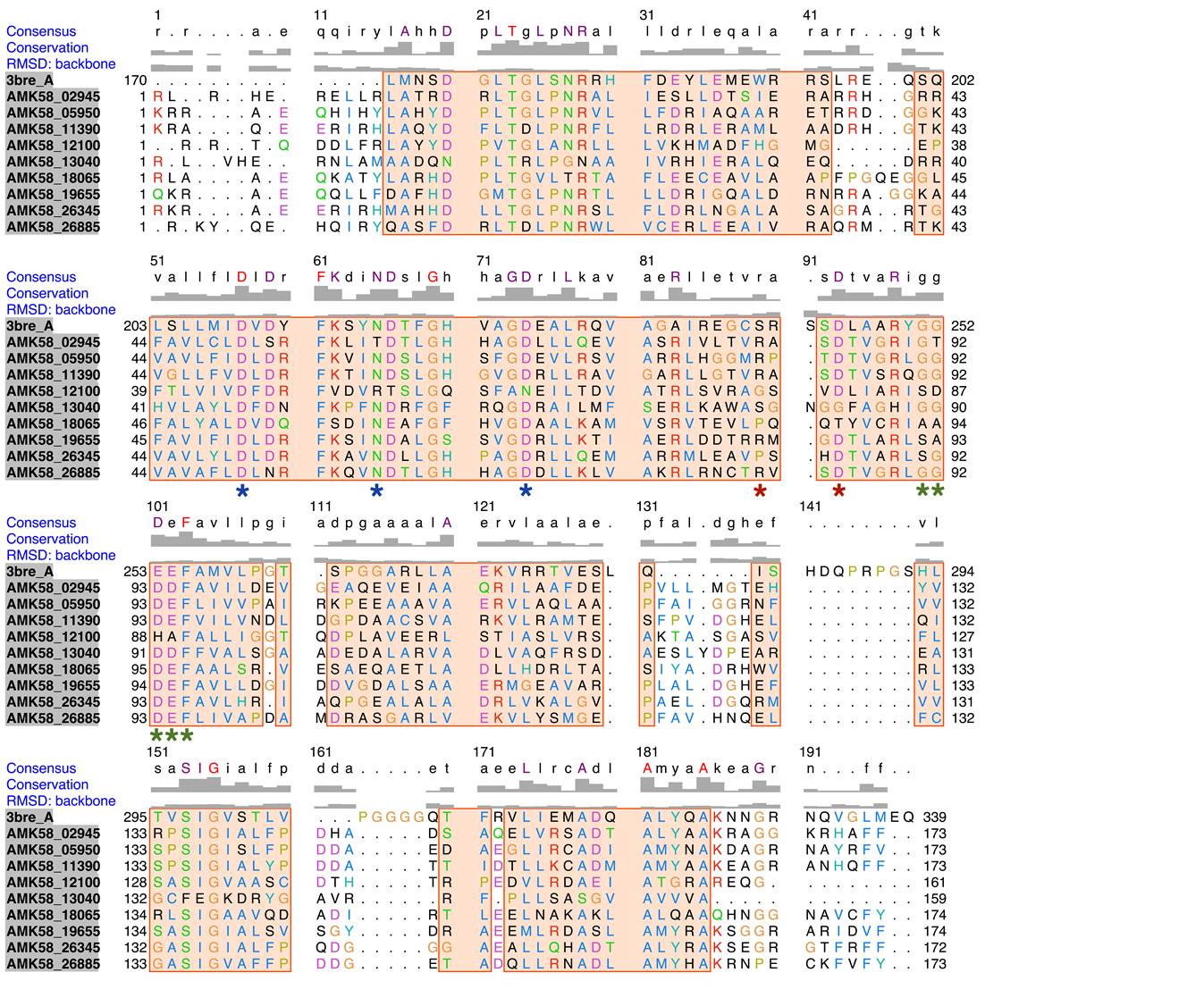


**3bre_A**

**WP_079285130**

**WP_051140186**

**WP_051140104**

**WP_059398931**

**WP_059399067**

**WP_051140628**

**WP_035678503**

**WP_059399655**

**WP_059399677**


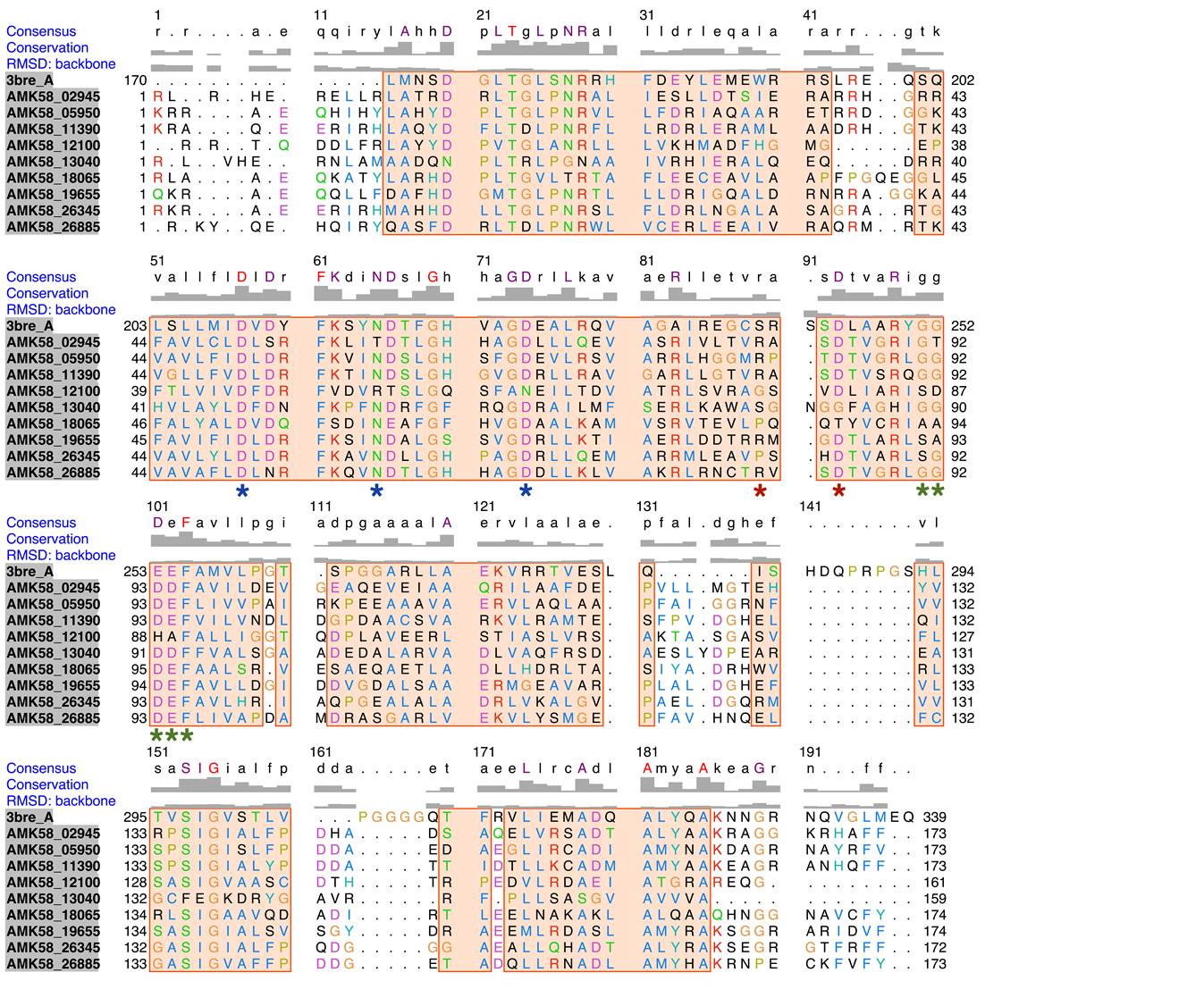


**3bre_A**

**WP_079285130**

**WP_051140186**

**WP_051140104**

**WP_059398931**

**WP_059399067**

**WP_051140628**

**WP_035678503**

**WP_059399655**

**WP_059399677**


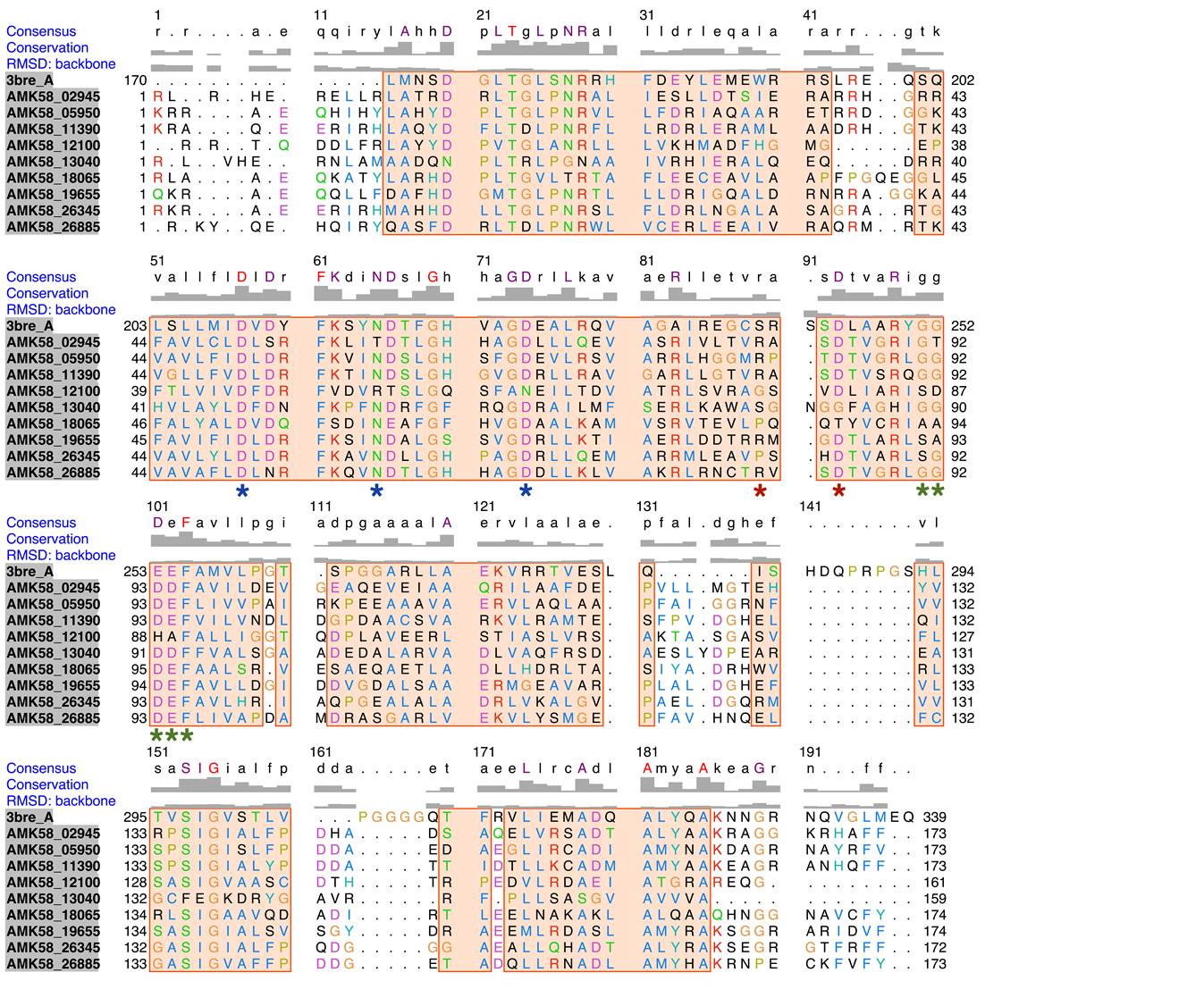


**3bre_A**

**WP_079285130**

**WP_051140186**

**WP_051140104**

**WP_059398931**

**WP_059399067**

**WP_051140628**

**WP_035678503**

**WP_059399655**

**WP_059399677**

1: 3bre_A 100.00 29.07 33.14 31.40 25.00 24.11 26.90 29.07 29.82 31.98

2: WP_079285130 29.07 100.00 42.77 42.77 26.09 24.64 28.49 41.62 44.19 41.04

3: WP_051140186 33.14 42.77 100.00 52.02 29.81 23.91 27.91 47.40 47.67 45.66

4: WP_051140104 31.40 42.77 52.02 100.00 27.95 24.64 28.49 41.62 45.35 47.40

5: WP_059398931 25.00 26.09 29.81 27.95 100.00 19.26 21.88 32.92 25.62 22.36

6: WP_059399067 24.11 24.64 23.91 24.64 19.26 100.00 25.55 25.90 24.09 24.64

7: WP_051140628 26.90 28.49 27.91 28.49 21.88 25.55 100.00 30.23 33.14 24.42

8: WP_035678503 29.07 41.62 47.40 41.62 32.92 25.90 30.23 100.00 48.84 36.99

9: WP_059399655 29.82 44.19 47.67 45.35 25.62 24.09 33.14 48.84 100.00 44.77

10: WP_059399677 31.98 41.04 45.66 47.40 22.36 24.64 24.42 36.99 44.77 100.00

**Table 7Sc. The alignments, root main square deviations (RMSD), and** **sequence conservation percentage of the proteins with EAL domain, encoded by genes found in the *A. brasilense* Sp7genome.**


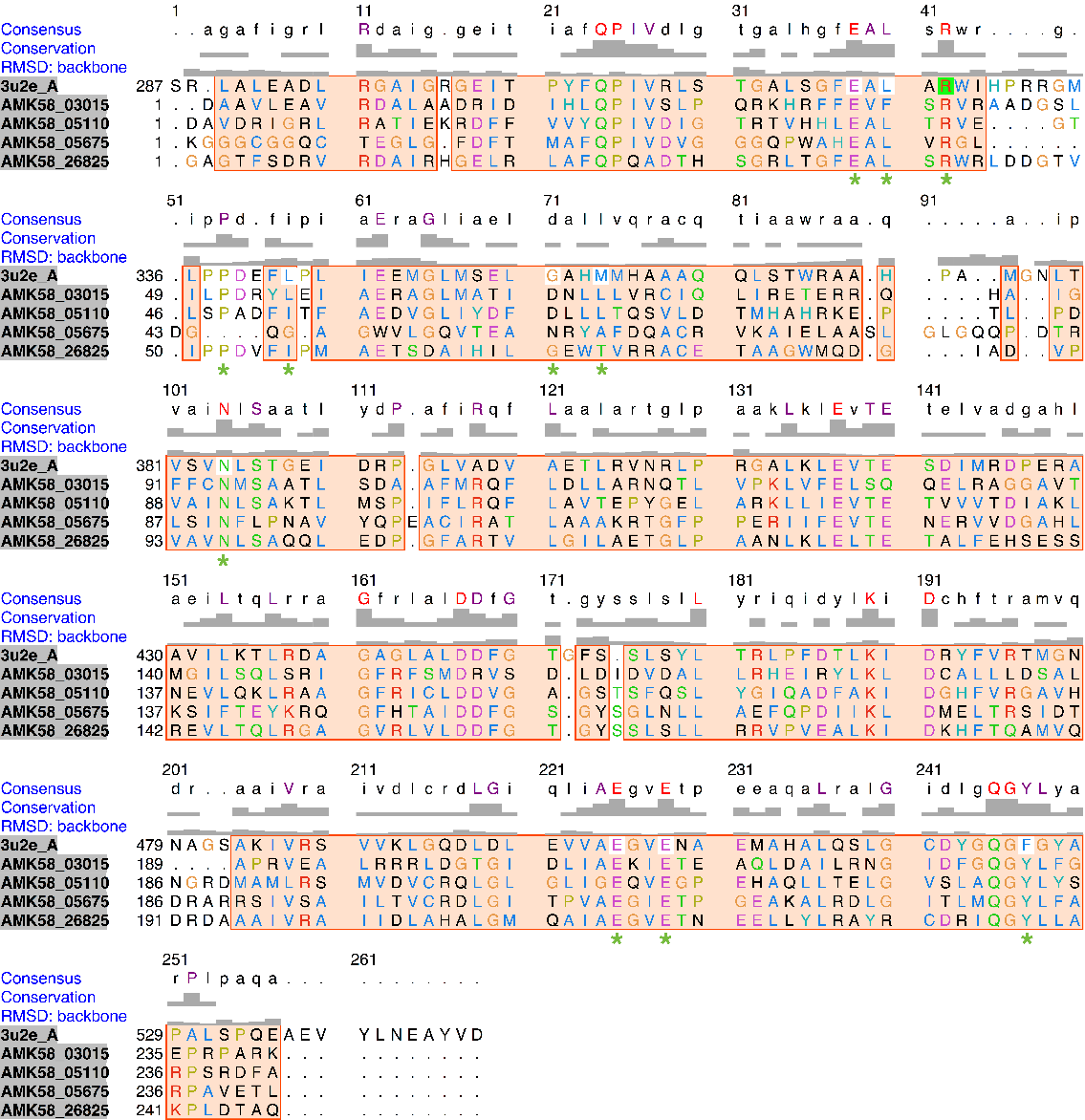

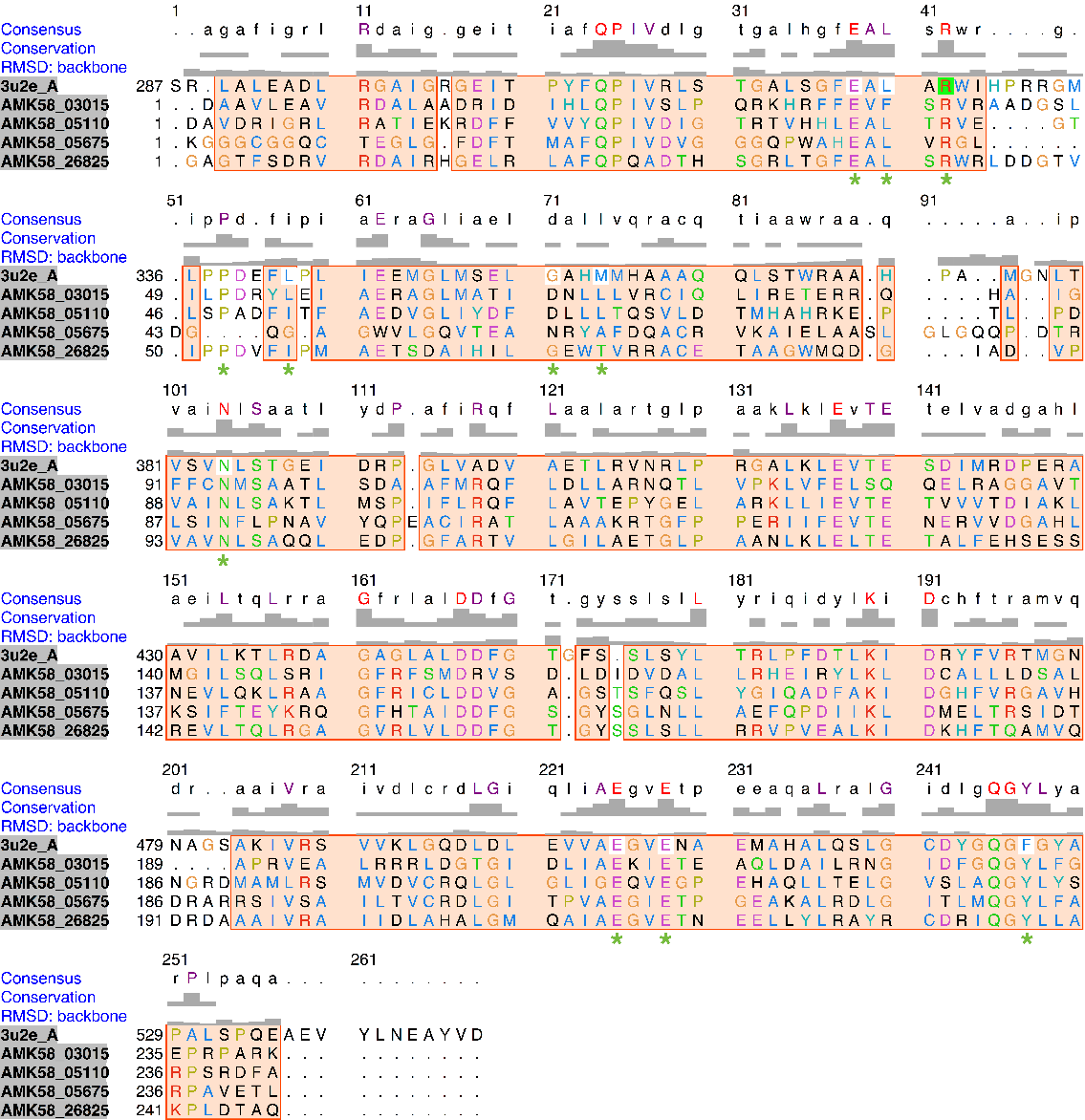


3u2e_A

WP_059398606

WP_051140161

WP_035672792

WP_035682417

3u2e_A

WP_059398606

WP_051140161

WP_035672792

WP_035682417

3u2e_A

WP_059398606

WP_051140161

WP_035672792

WP_035682417

3u2e_A

WP_059398606

WP_051140161

WP_035672792

WP_035682417

3u2e_A

WP_059398606

WP_051140161

WP_035672792

WP_035682417

3u2e_A

WP_059398606

WP_051140161

WP_035672792

WP_035682417


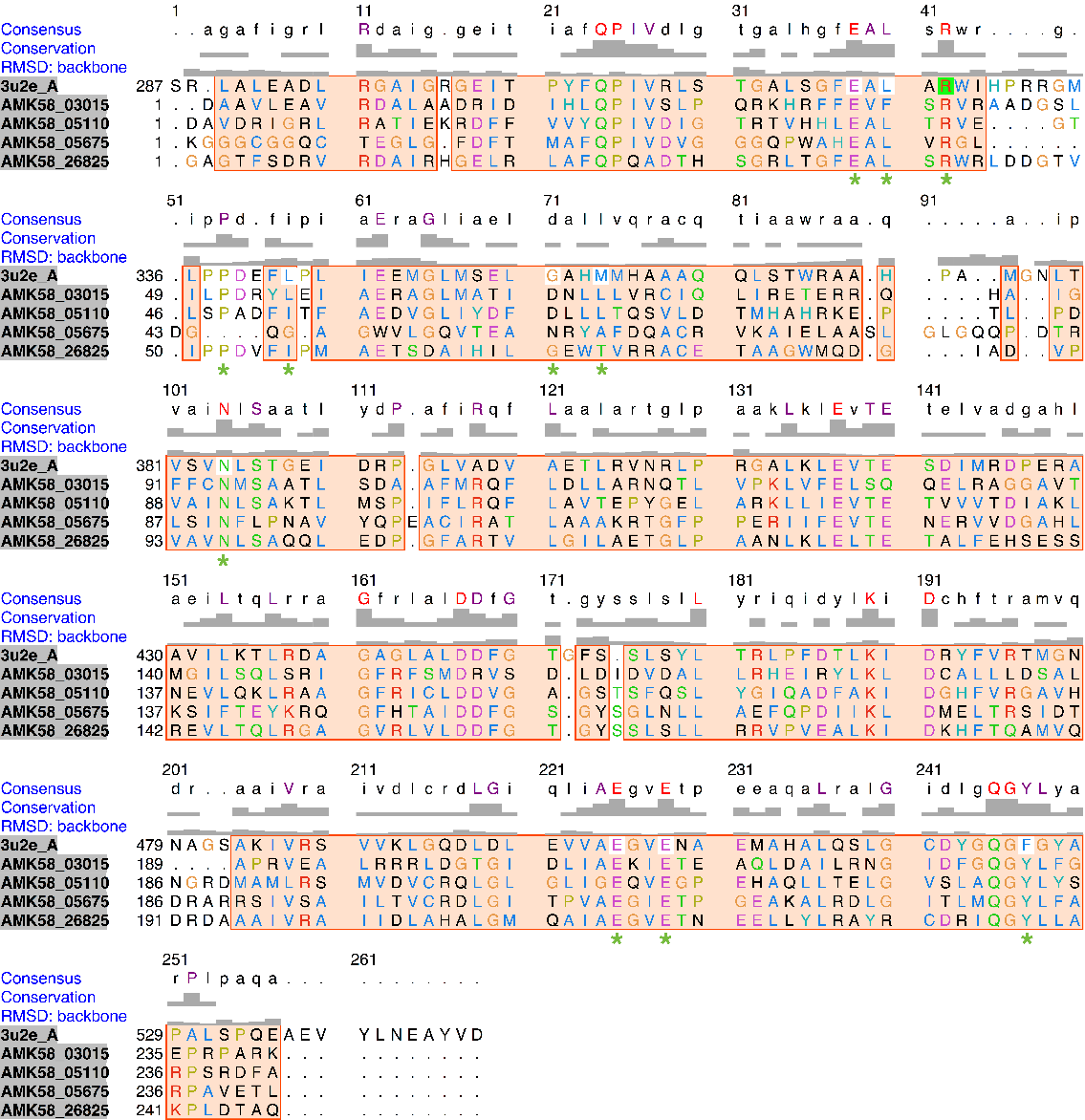

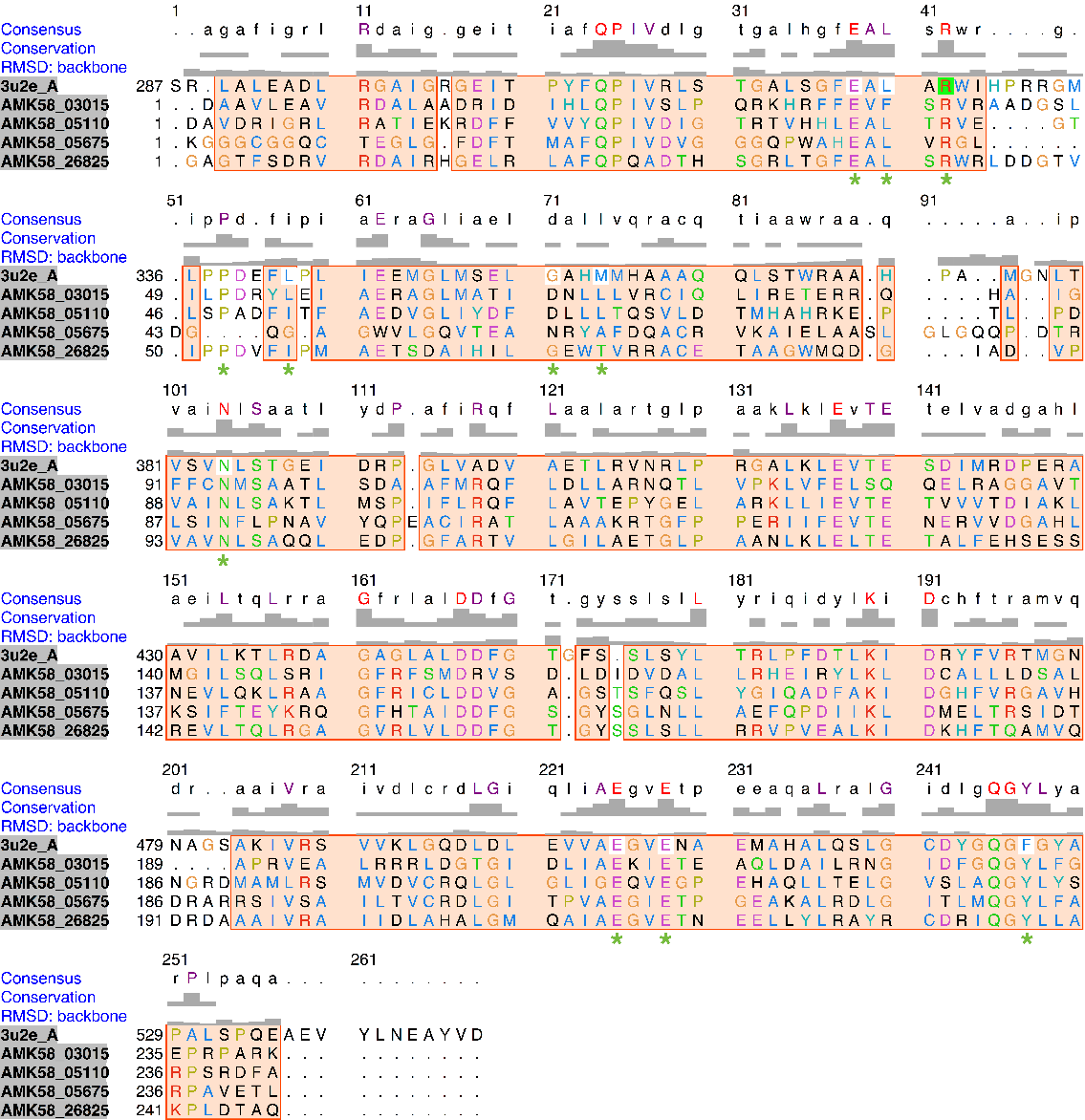

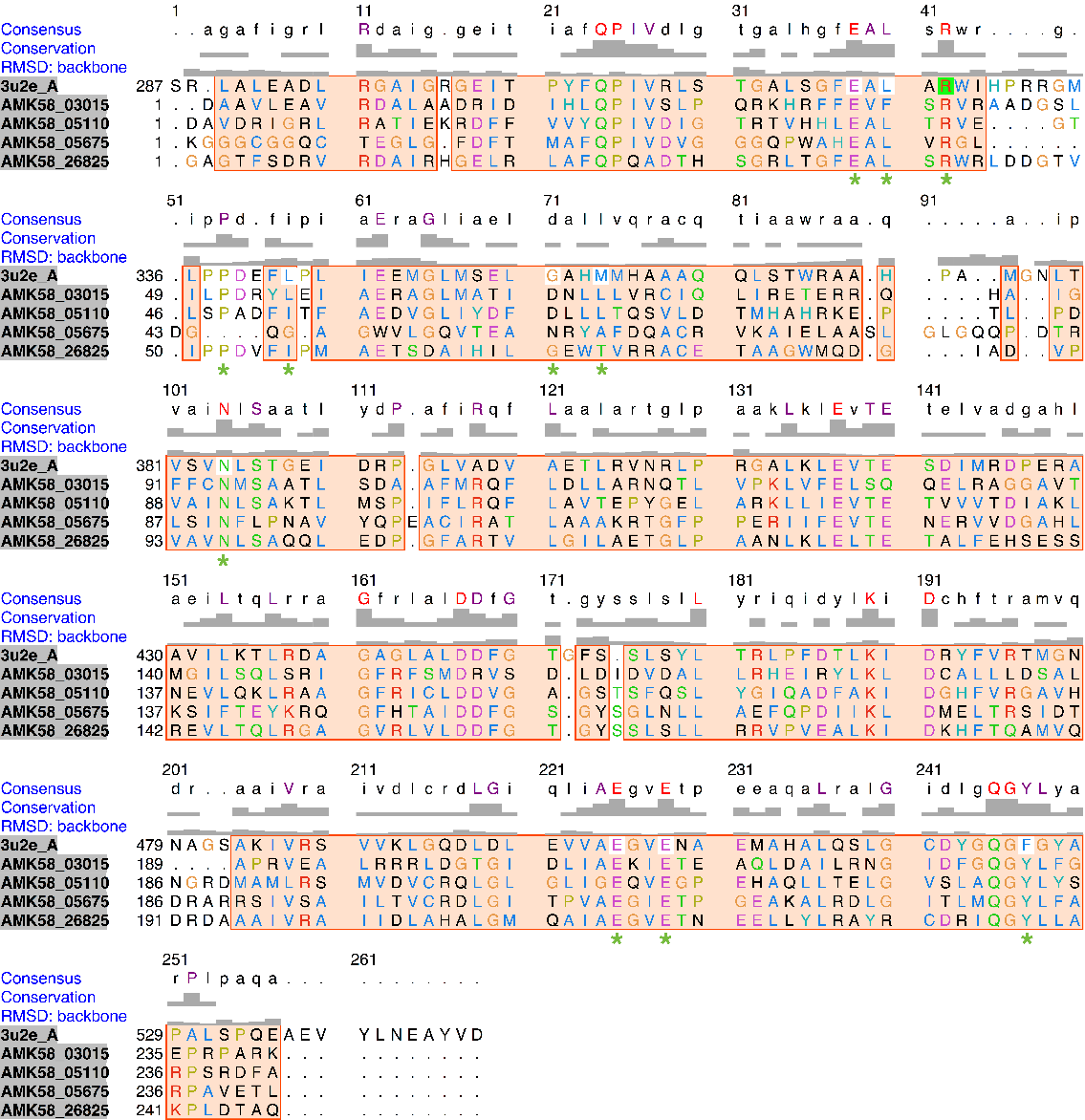

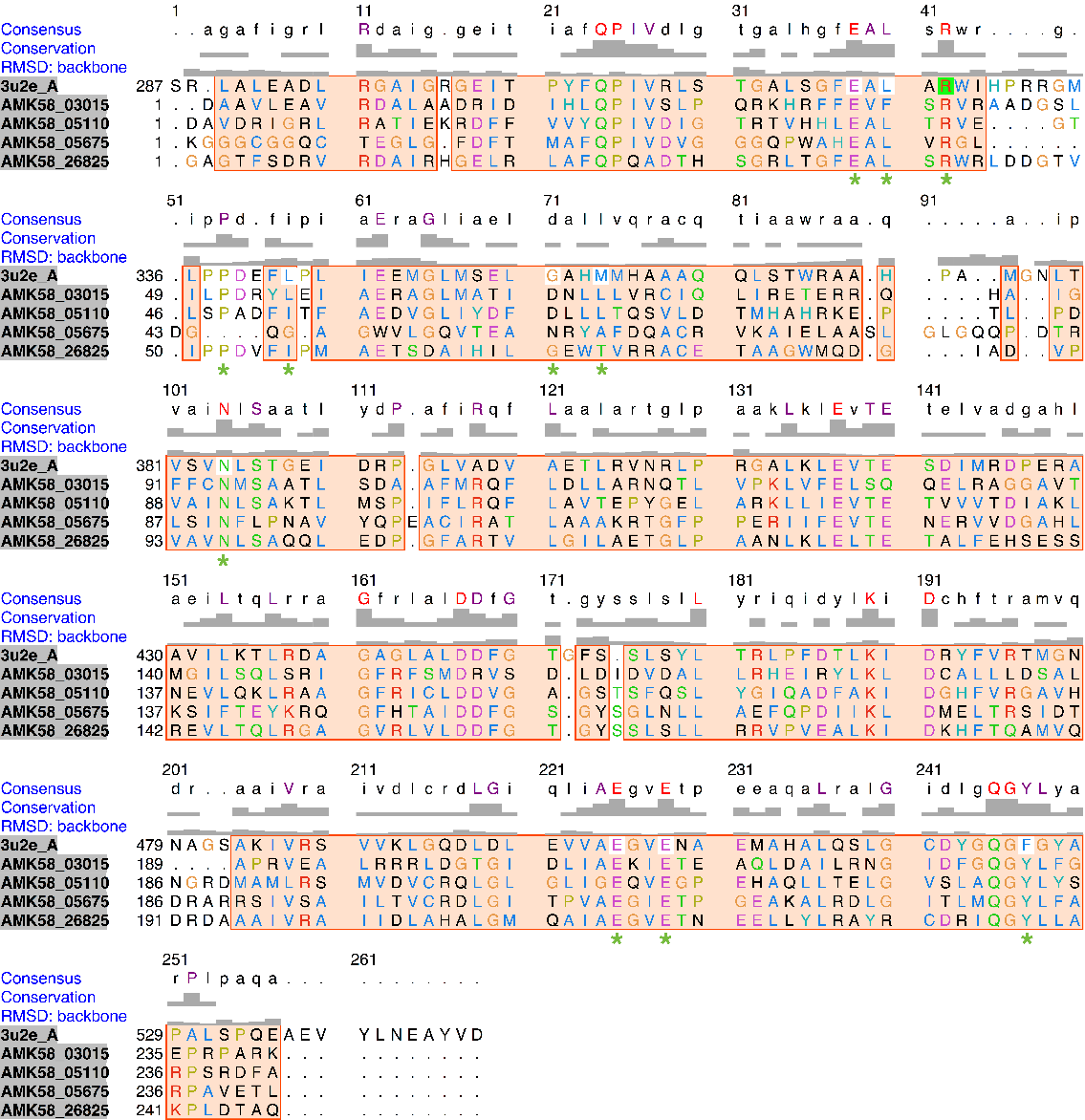

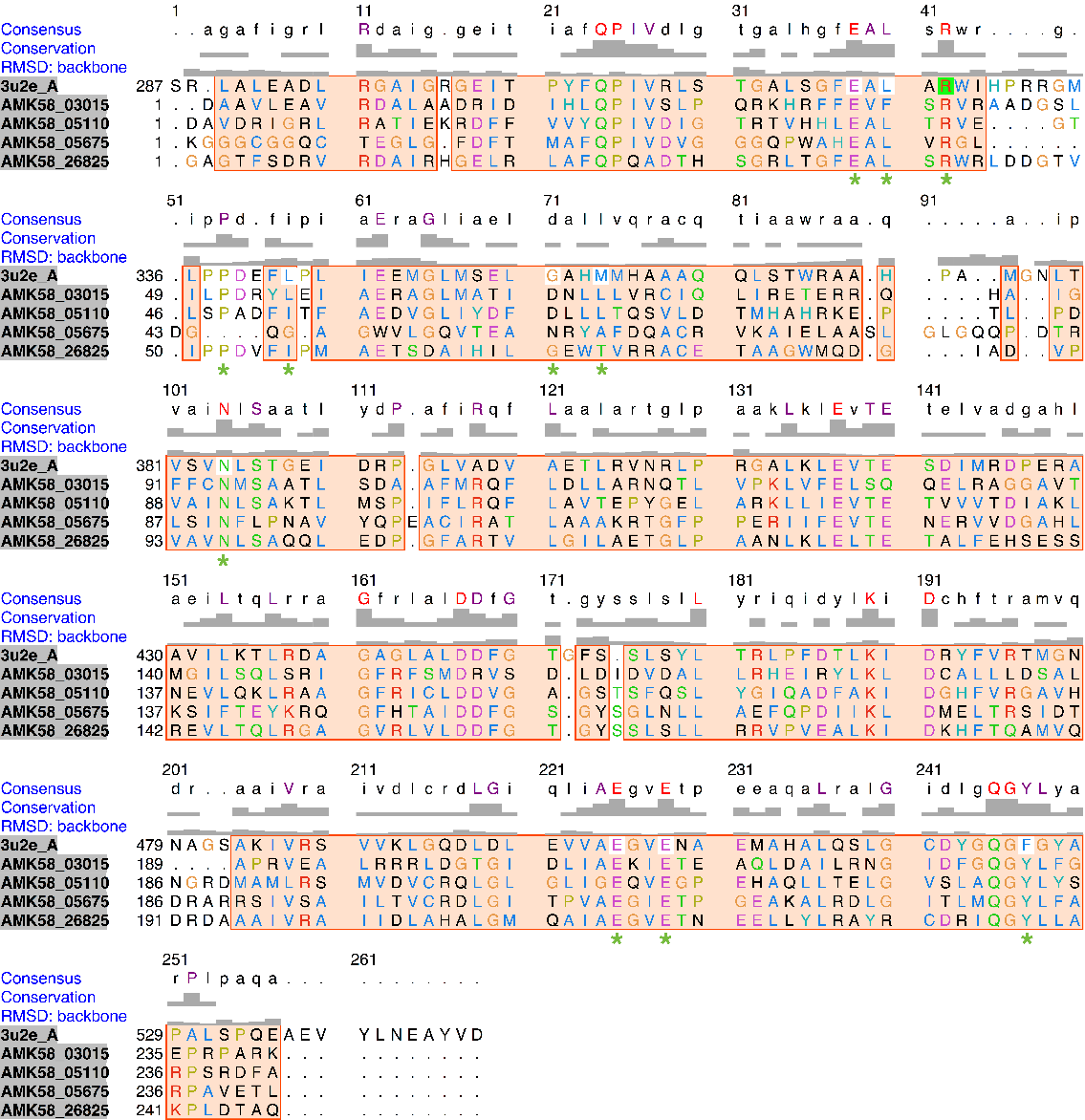


1: 3u2e_A 100.00 19.50 28.10 23.95 36.44

2: WP_059398606 19.50 100.00 22.46 20.43 25.00

3: WP_051140161 28.10 22.46 100.00 25.97 30.42

4: WP_035672792 23.95 20.43 25.97 100.00 27.12

5: WP_035682417 36.44 25.00 30.42 27.12 100.00

**Table 7Sd. The alignments, root main square deviations (RMSD), and** **sequence conservation percentage of the hybrid proteins with EAL domain encoded, by genes found in the *A. brasilense* Sp7 genome.**


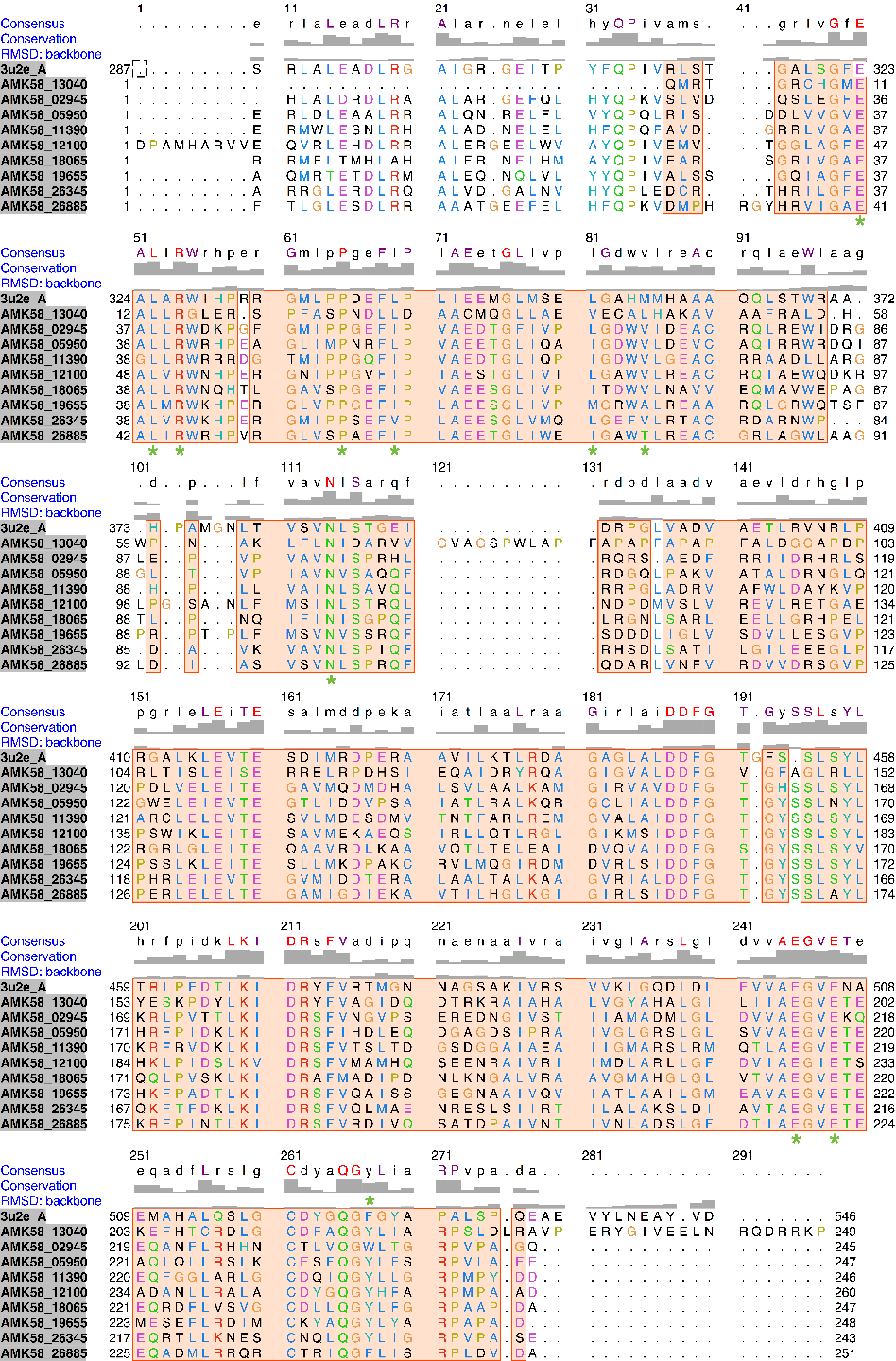


**3u2e_A**

**WP_059399067**

**WP_079285130**

**WP_051140186**

**WP_051140104**

**WP_059398931**

**WP_051140628**

**WP_035678503**

**WP_059399655**

**WP_059399677**


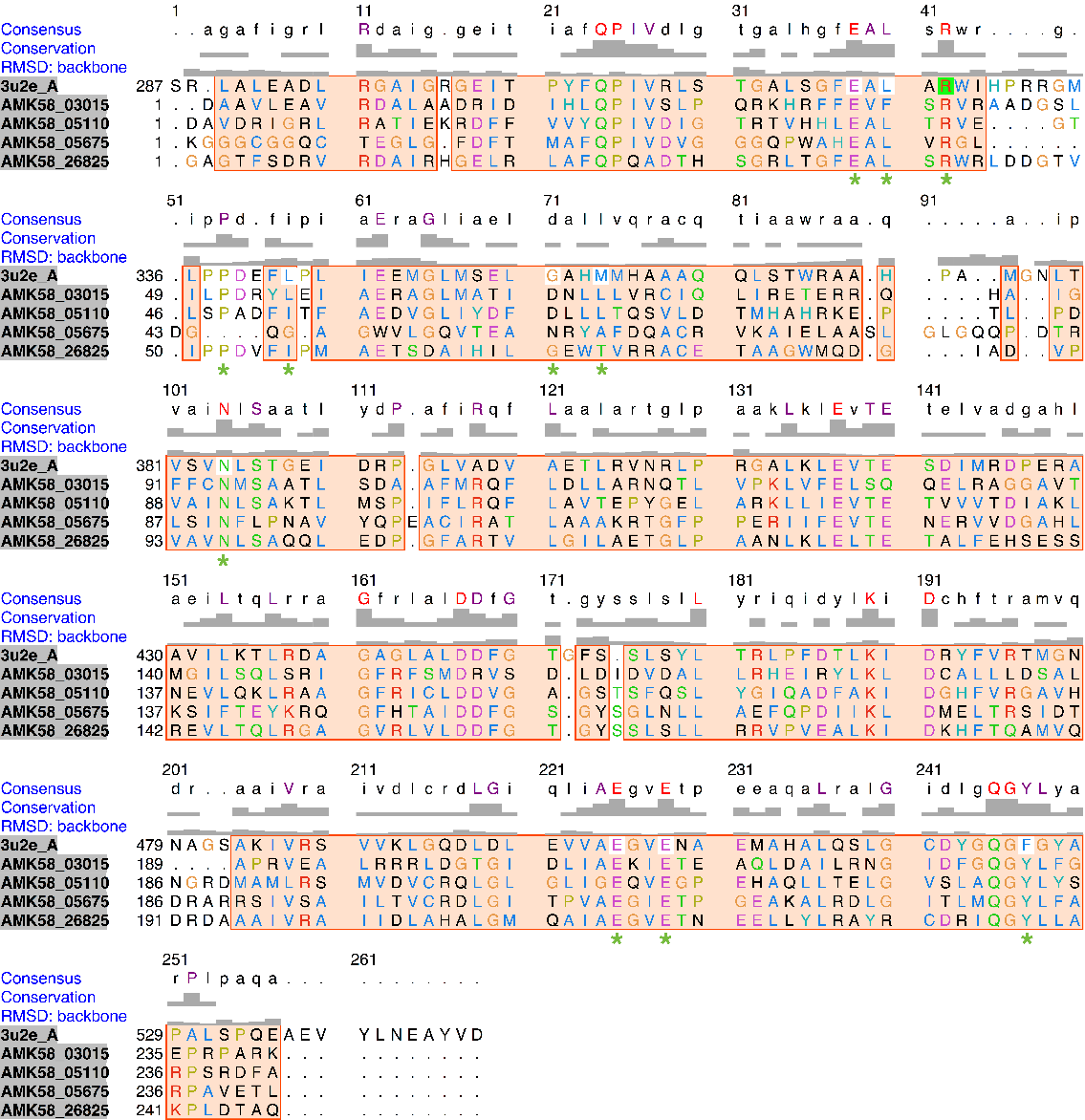


**3u2e_A**

**WP_059399067**

**WP_079285130**

**WP_051140186**

**WP_051140104**

**WP_059398931**

**WP_051140628**

**WP_035678503**

**WP_059399655**

**WP_059399677**


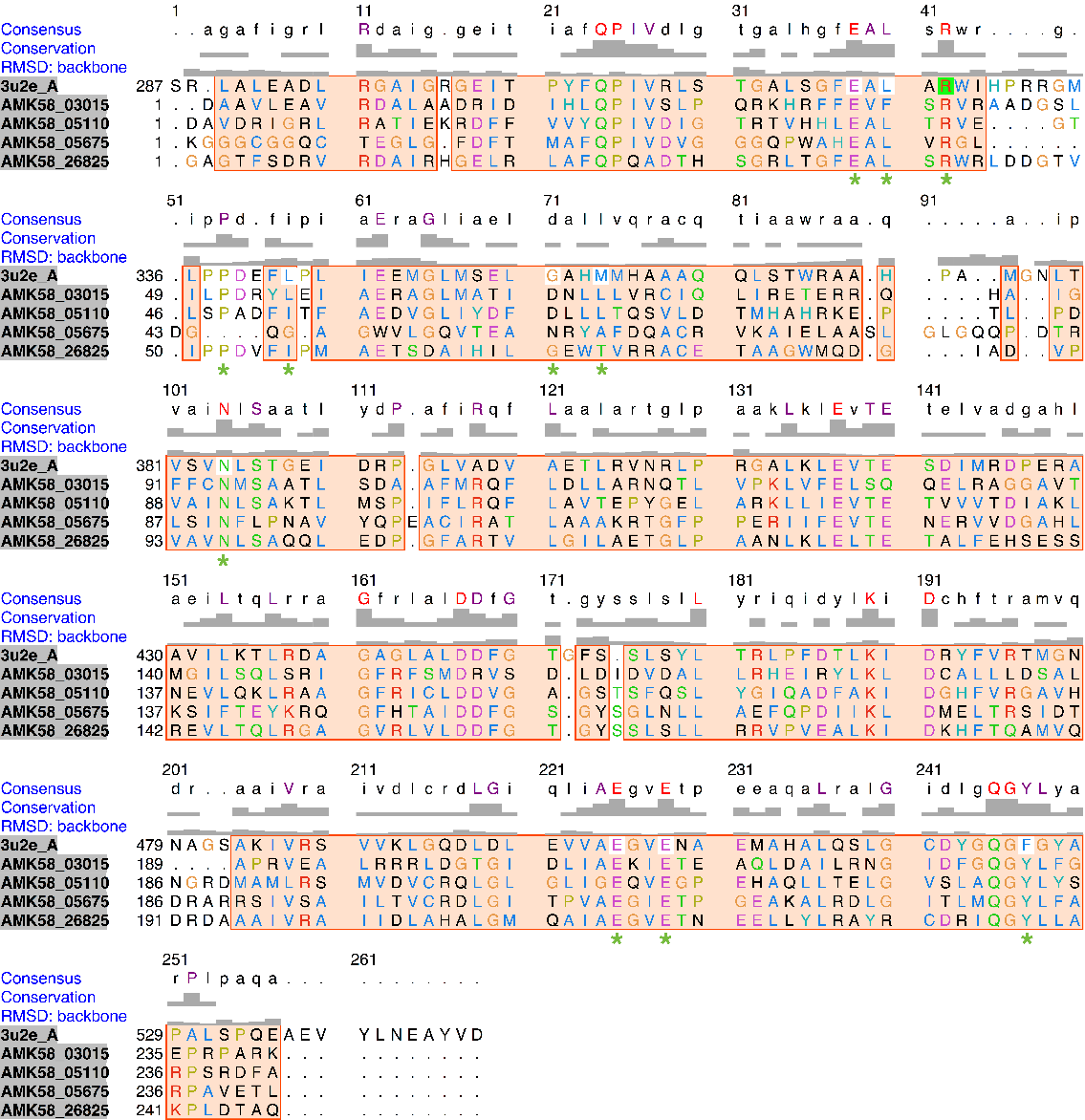

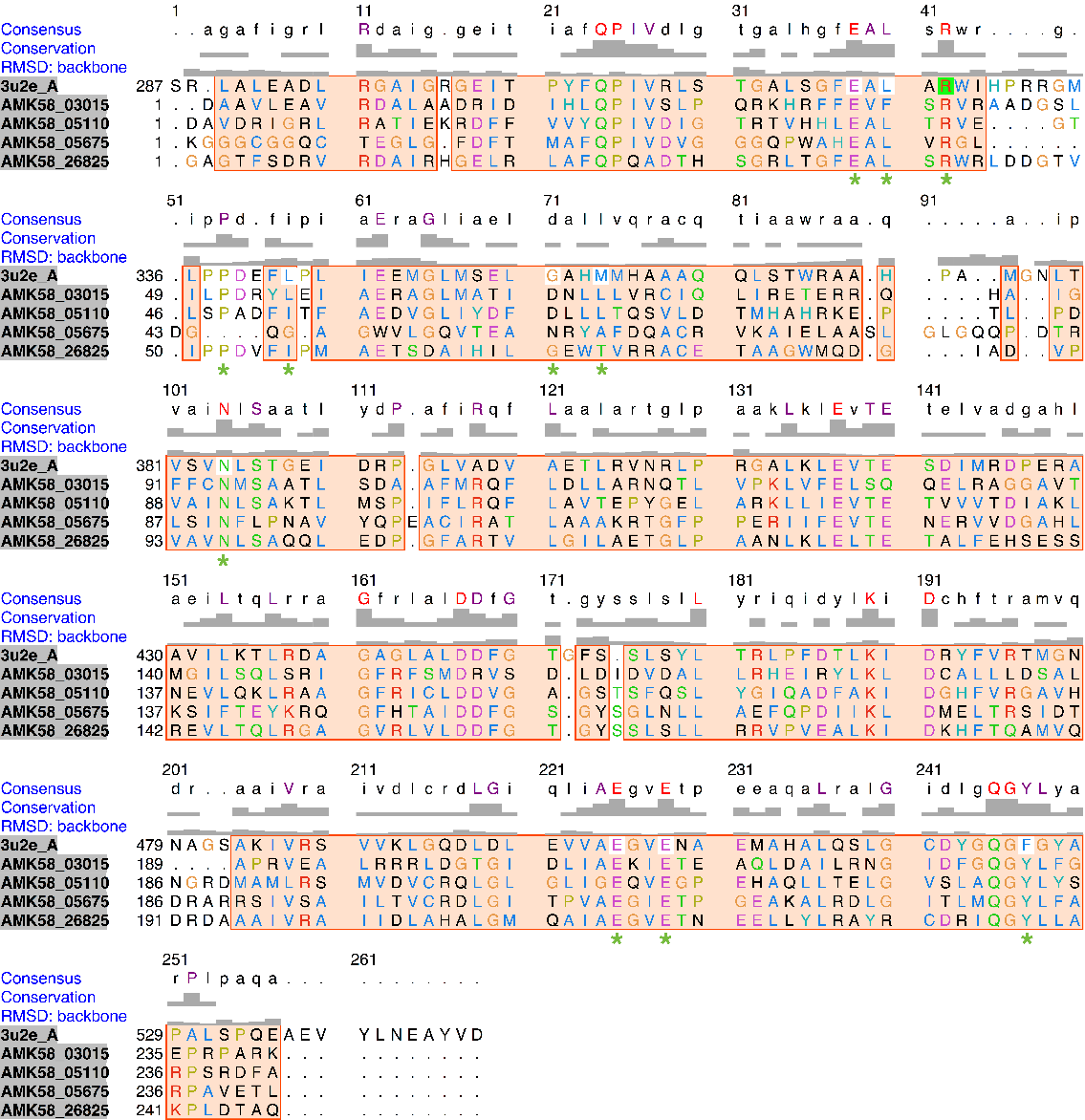


**3u2e_A**

**WP_059399067**

**WP_079285130**

**WP_051140186**

**WP_051140104**

**WP_059398931**

**WP_051140628**

**WP_035678503**

**WP_059399655**

**WP_059399677**

**3u2e_A**

**WP_059399067**

**WP_079285130**

**WP_051140186**

**WP_051140104**

**WP_059398931**

**WP_051140628**

**WP_035678503**

**WP_059399655**

**WP_059399677**

**3u2e_A**

**WP_059399067**

**WP_079285130**

**WP_051140186**

**WP_051140104**

**WP_059398931**

**WP_051140628**

**WP_035678503**

**WP_059399655**

**WP_059399677**

**3u2e_A**

**WP_059399067**

**WP_079285130**

**WP_051140186**

**WP_051140104**

**WP_059398931**

**WP_051140628**

**WP_035678503**

**WP_059399655**

**WP_059399677**


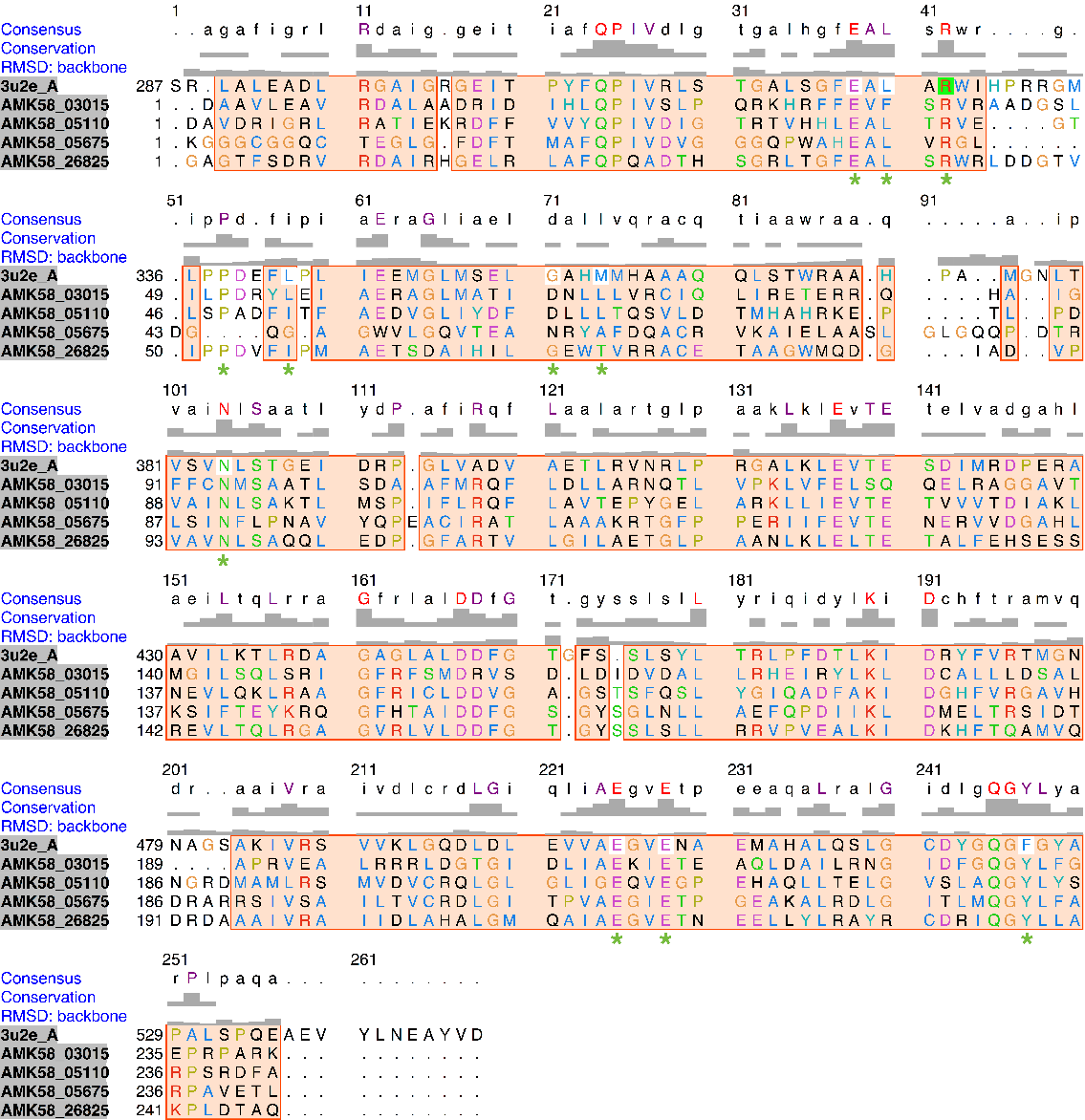

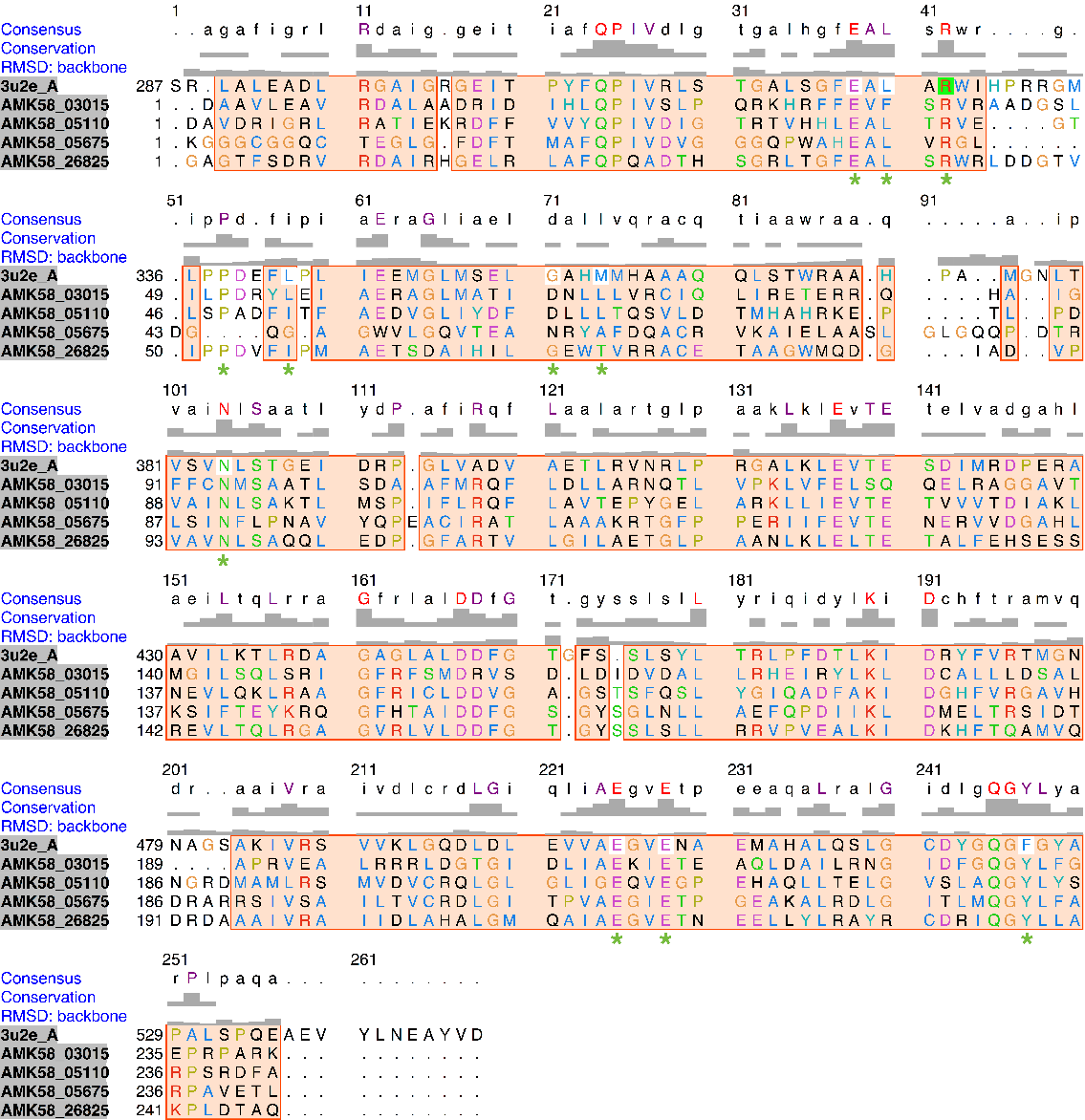

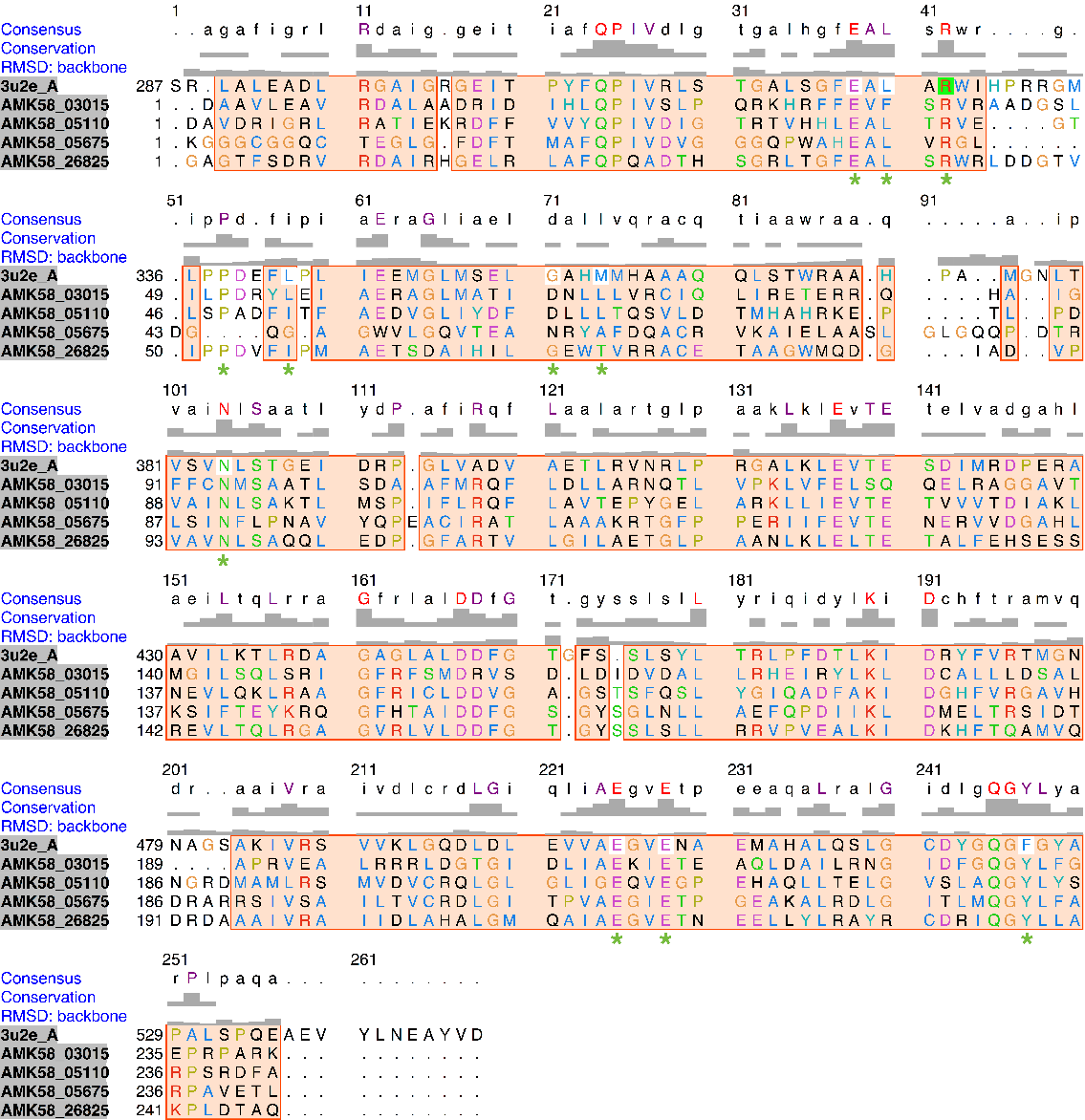


**# # # Percent Identity Matrix - created by Clustal2.1 # #**

**1: 3U2E_A 100.00 29.02 38.93 39.43 36.18 43.20 36.18 42.57 42.56 41.46**

**2: AMH58_13040 29.02 100.00 26.17 28.37 30.37 29.30 32.56 28.97 27.91 29.77**

**3: AMK58_02945 38.93 26.17 100.00 44.08 44.26 45.71 40.41 41.80 46.89 47.76**

**4: AMK58_05950 39.43 28.37 44.08 100.00 46.75 42.91 41.30 41.06 49.38 45.75**

**5: AMK58_11390 36.18 30.37 44.26 46.75 100.00 42.28 43.09 42.68 43.80 45.53**

**6: AMK58_12100 43.20 29.30 45.71 42.91 42.28 100.00 38.06 54.22 44.03 47.98**

**7: AMK58_18065 36.18 32.56 40.41 41.30 43.09 38.06 100.00 41.87 39.51 40.49**

**8: AMK58_19655 42.57 28.97 41.80 41.06 42.68 54.22 41.87 100.00 45.04 48.37**

**9: AMK58_26345 42.56 27.91 46.89 49.38 43.80 44.03 39.51 45.04 100.00 46.09**

**10: AMK58_26885 41.46 29.77 47.76 45.75 45.53 47.98 40.49 48.37 46.09 100.00**

1: 3u2e_A 100.00 29.02 38.93 39.43 36.18 43.20 36.18 42.57 42.56 41.46

2: WP_059399067 29.02 100.00 26.17 28.37 30.37 29.30 32.56 28.97 27.91 29.77

3: WP_079285130 38.93 26.17 100.00 44.08 44.26 45.71 40.41 41.80 46.89 47.76

4: WP_051140186 39.43 28.37 44.08 100.00 46.75 42.91 41.30 41.06 49.38 45.75

5: WP_051140104 36.18 30.37 44.26 46.75 100.00 42.28 43.09 42.68 43.80 45.53

6: WP_059398931 43.20 29.30 45.71 42.91 42.28 100.00 38.06 54.22 44.03 47.98

7: WP_051140628 36.18 32.56 40.41 41.30 43.09 38.06 100.00 41.87 39.51 40.49

8: WP_035678503 42.57 28.97 41.80 41.06 42.68 54.22 41.87 100.00 45.04 48.37

9: WP_059399655 42.56 27.91 46.89 49.38 43.80 44.03 39.51 45.04 100.00 46.09

10: WP_059399677 41.46 29.77 47.76 45.75 45.53 47.98 40.49 48.37 46.09 100.00
